# An increase of lysosomes through EGF-triggered endocytosis attenuated zinc-mediated lysosomal membrane permeabilization and neuronal cell death

**DOI:** 10.1038/s41419-024-07192-6

**Published:** 2024-11-13

**Authors:** Jae-Won Eom, Jin-Yeon Lee, Yeabin Kwon, Yang-Hee Kim

**Affiliations:** Department of Integrative Bioscience and Biotechnology, Sejong University, Seoul, 05006 Republic of Korea

**Keywords:** Cell death in the nervous system, Stroke

## Abstract

In the context of acute brain injuries, where zinc neurotoxicity and oxidative stress are acknowledged contributors to neuronal damage, we investigated the pivotal role of lysosomes as a potential protective mechanism. Our research commenced with an exploration of epidermal growth factor (EGF) and its impact on lysosomal dynamics, particularly its neuroprotective potential against zinc-induced cytotoxicity. Using primary mouse cerebrocortical cultures, we observed the rapid induction of EGFR endocytosis triggered by EGF, resulting in a transient increase in lysosomal vesicles. Furthermore, EGF stimulated lysosomal biogenesis, evident through elevated expression of lysosomal-associated membrane protein 1 (LAMP-1) and the induction and activation of prominent lysosomal proteases, particularly cathepsin B (CTSB). This process of EGFR endocytosis was found to promote lysosomal augmentation, thus conferring protection against zinc-induced lysosomal membrane permeabilization (LMP) and subsequent neuronal death. Notably, the neuroprotective effects and lysosomal enhancement induced by EGF were almost completely reversed by the inhibition of clathrin-mediated and caveolin-mediated endocytosis pathways, along with the disruption of retrograde trafficking. Furthermore, tyrosine kinase inhibition of EGFR nullified EGFR endocytosis, resulting in the abrogation of EGF-induced lysosomal upregulation and neuroprotection. An intriguing aspect of our study is the successful replication of EGF’s neuroprotective effects through the overexpression of LAMP-1, which significantly reduced zinc-induced LMP and cell death, demonstrated in both primary mouse cerebrocortical neuronal cultures and human embryonic kidney (HEK) cells. Our research extended beyond zinc-induced neurotoxicity, as we observed EGF’s protective effects against other oxidative stressors linked to intracellular zinc release, including hydrogen peroxide (H_2_O_2_) and 1-methyl-4-phenylpyridinium ion (MPP^+^). Collectively, our findings unveil the intricate interplay between EGF-triggered EGFR endocytosis, lysosomal upregulation, an increase in the regulatory capacity for zinc homeostasis, and the subsequent alleviation of zinc-induced neurotoxicity. These results present promising avenues for therapeutic interventions to enhance neuroprotection by targeting lysosomal augmentation.

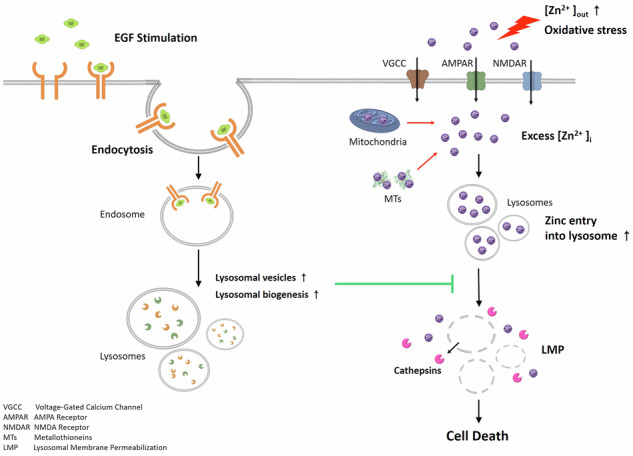

## Introduction

Zinc, an essential metal ion, exerts a crucial regulatory role over numerous proteins within cells and organisms. Proteomic analysis reveals that around 10% of proteins possess zinc-binding motifs, and their functions are under the influence of zinc [1, 2]. In the central nervous system (CNS), zinc is highly concentrated within the synaptic vesicles of glutamatergic neurons, primarily in its free ion form, unbound to proteins [3–5]. During synaptic activity, zinc is co-released with glutamate into the synaptic cleft, potentially influencing the activity of post-synaptic neurons [6, 7].

In cases of acute brain injuries, such as stroke, traumatic brain injury, and epilepsy, synaptic zinc is not promptly reuptake into cells, resulting in its excessive entry into post-synaptic neurons [3, 8–10]. This excessive zinc influx triggers the over-activation of kinases like protein kinase C (PKC), Src, and extracellular signal-regulated kinase (ERK), as well as NADPH oxidase and poly(ADP-ribose) polymerase, ultimately leading to neuronal death [11–14]. Zinc-induced neurotoxicity, combined with calcium-mediated excitotoxicity, oxidative stress, and apoptosis, constitutes a central mechanism in acute brain injuries [8, 13, 15–17].

Lysosomes are cellular organelles responsible for intracellular digestion and degradation. Their function can deteriorate due to factors like aging or various genetic causes [18–20]. When lysosomal function declines, specific proteins, including tau, α-synuclein, and transactive response DNA binding protein of 43 (TDP-43), can accumulate within nerve cells, contributing to the onset of neurodegenerative diseases such as Alzheimer’s disease (AD), Parkinson’s disease (PD), and amyotrophic lateral sclerosis (ALS). Maintaining an acidic pH within lysosomes is crucial, and this process relies on the action of the zinc transporters [21, 22]. Zinc also plays a role in rapidly facilitating the assembly of v-ATPase on lysosomes, promoting acidification. Additionally, zinc induces the activation of the transcription factor EB (TF-EB), which, in turn, leads to lysosomal biosynthesis [23]. Similar to mitochondria, lysosomes play a crucial role in maintaining zinc homeostasis by sequestering excess zinc when cytosolic zinc concentrations rise. However, in pathological conditions, a sudden and significant increase in intracellular zinc can accelerate its movement into lysosomes, overwhelming and damaging them, ultimately leading to lysosomal membrane permeabilization (LMP) [24]. When LMP occurs, lysosomal degradative enzymes like cathepsin B (CTSB) are released into the cytoplasm, triggering additional mechanisms of neuronal damage, including the activation of caspase-3 to execute apoptosis and the formation of inflammasomes to induce neuroinflammation [25–27].

Elevated intracellular zinc levels can originate from external sources or extracellular space, and they can also be a consequence of increased oxidative stress, which releases zinc from zinc-binding proteins. This process results in an increased concentration of labile zinc in the cytoplasm [28]. Although Metallothionein (MT) proteins are typically responsible for binding zinc and maintaining zinc homeostasis in the cytoplasm, heightened oxidative stress, such as elevated hydrogen peroxide (H_2_O_2_) levels, can lead to the release of zinc from MT proteins [29–32]. Consequently, this release triggers LMP and subsequent neuronal cell death [33]. In the context of PD, mitochondrial toxins like 1-methyl-4-phenyl-1,2,3,6-tetrahydropyridine (MPTP) and rotenone are implicated in causing damage to dopaminergic neurons, potentially contributing to the disease’s progression [34–36]. Studies have indicated that the administration of MPP^+^ in in vitro neuronal cell models or MPTP in in vivo mouse animal models results in increased oxidative stress and the induction of LMP [37, 38]. This, in turn, can lead to lysosomal deficiency and expedite the accumulation of α-synuclein proteins. In essence, lysosomes are involved in the mechanisms of neuronal cell death, both in cases of acute brain injury mediated by LMP and in instances of lysosomal dysfunction and the accumulation of protein aggregates in neurodegenerative diseases.

In our study, we aimed to investigate whether the quantitative regulation of lysosomes could effectively control zinc-induced neurotoxicity. Epidermal growth factor (EGF) is a well-known growth factor that stimulates cell proliferation. It is recognized for its ability to bind to its receptor and, upon initiating signal transduction, undergoes rapid endocytosis along with its receptor EGFR, ultimately being transported to lysosomes for degradation [39, 40]. This process involves an increase in vesicles such as endosomes, resulting in a temporary rise in the number of lysosomes. Furthermore, studies have demonstrated that promoting endocytosis can activate the transcriptional activity of TF-EB, a transcription factor responsible for lysosomal biogenesis [41]. Hence, we chose EGF as a stimulant for increasing lysosomal quantity, given its potential to contribute to the upregulation of lysosomes via endocytosis and TF-EB-mediated lysosomal biogenesis.

In the initial phase of our research, we conducted experiments to determine whether the application of EGF to primary mouse cerebrocortical neuronal cultures could indeed lead to an increase in lysosomes. Once we confirmed this increase, our investigation delved further into whether EGF could mitigate zinc toxicity and whether this protective effect was linked to improved regulation of zinc homeostasis achieved through a quantitative augmentation of lysosomes, resulting in a reduction in LMP. Additionally, we explored whether the EGF-induced increase in lysosomes could also contribute to the suppression of oxidative damage, which is responsible for the release of cytosolic zinc. These findings shed light on the critical role of lysosomes in neuronal death, not only in the context of neurodegenerative diseases but also in acute brain injury. Ultimately, these insights may pave the way for the development of therapeutic strategies targeting lysosomal activation for acute brain diseases.

## Results

### EGF treatment enhances lysosomal dynamics in mouse cerebrocortical cultures

Our investigation began with an exploration of the impact of EGF treatment on lysosomal dynamics in mouse cerebrocortical cultures. EGF is widely recognized for its ability to induce EGFR endocytosis and the subsequent increase in endocytic vesicles [39]. We aimed to establish whether this led to a surge in lysosomal vesicle abundance. Before examining the increase in lysosomes induced by EGF, we confirmed that EGFR was sufficiently expressed in mouse cerebrocortical neurons and astrocytes. By verifying high EGFR expression in both mixed cortical cultures (containing both astrocytes and neurons) and near-pure neuronal cultures (predominantly composed of neurons) (Supplementary Figure 1), we established that the response to EGF could be observed in both neurons and astroglial cells.

**Fig. 1:**
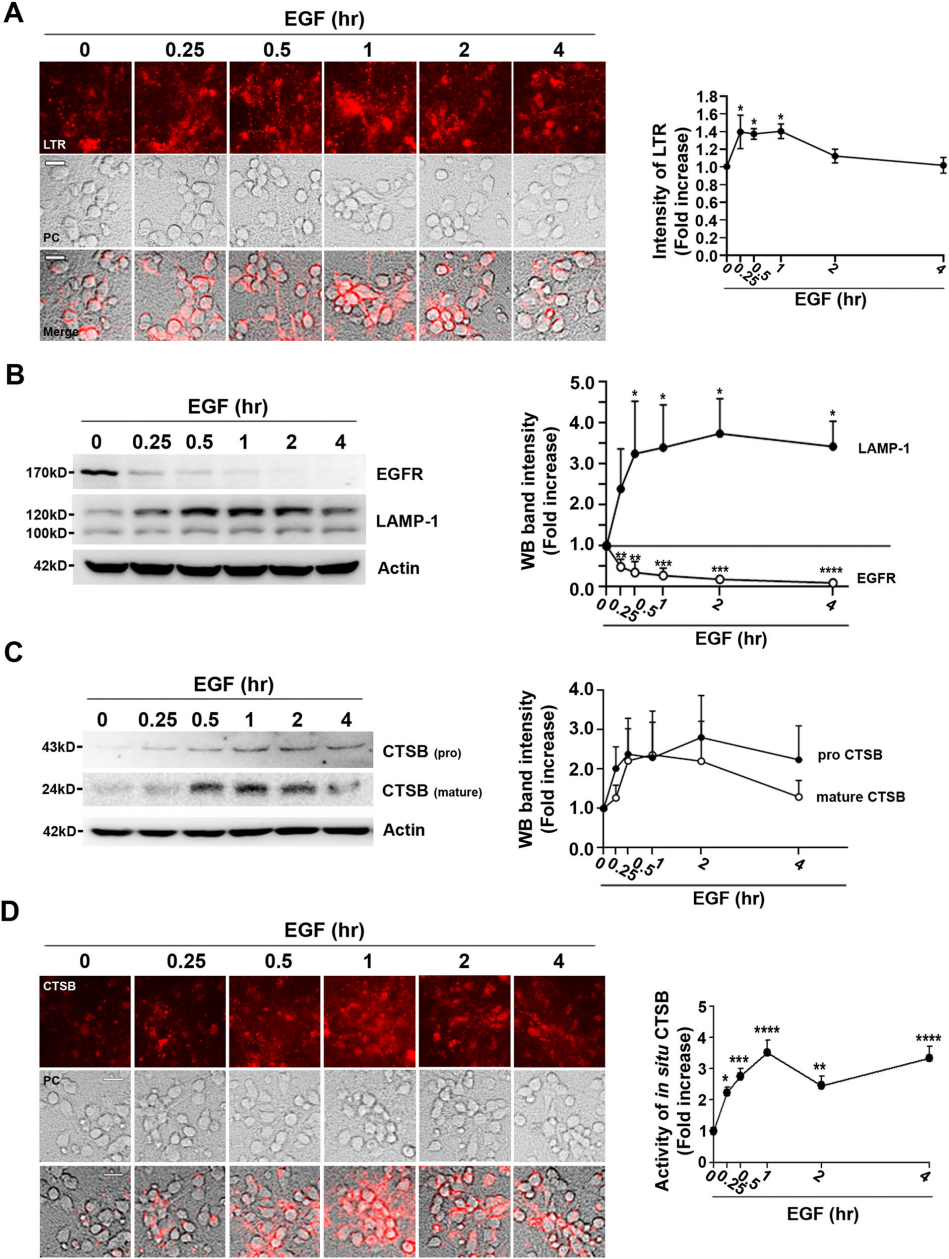
Rapid increase in lysosomes in mouse cerebrocortical cultures upon EGF treatment. **A** Microscopic images (left) and a quantitative graph (right) depict the time-dependent elevation in lysotracker red (LTR) fluorescence following treatment with 100 ng/ml recombinant mouse EGF. Scale bar: 20 µm. The line graph represents LTR intensity measured using the Image J program (mean ± SEM, *n* = 32 different fields taken from ≥4 independent biological replicate experiments). *p < 0.05; compared with 0-h time point using ANOVA with Dunnett’s post-hoc test. **B** Western blot analysis (left) and quantitative graph (right) show changes in the protein levels of EGF receptor (EGFR) and lysosomal associated membrane protein-1 (LAMP-1) over time after EGF treatment. Actin was used as the loading control. The line graph depicts the band density ratio of total LAMP-1 to Actin (closed circle) and EGFR to Actin (open circle) over time (mean ± SEM, *n* = 3 independent biological replicate experiments, *p < 0.05, **p < 0.01, ***p < 0.001, or ****p < 0.0001; compared with the 0-hour time point using ANOVA with Dunnett’s post-hoc test). **C** Western blot analysis (left) and quantitative graph (right) display the protein levels of CTSB over time after EGF treatment. The line graph depicts the band density ratio of pro-form (closed circle) or mature form of CTSB (open circle) to Actin over time (mean ± SEM, *n* = 6 independent biological replicate experiments). **D** Microscopic images (left) and a quantitative graph (right) reveal the time-dependent increase in in situ CTSB activity following treatment with 100 ng/ml EGF. Scale bar: 20 µm. The line graph represents CTSB activity determined using the Image J program (mean ± SEM, *n* = 34 different fields taken from ≥4 independent biological replicate experiments, *p < 0.05 **p < 0.01, ***p < 0.001, or ****p < 0.0001; compared with 0-hour time point using ANOVA with Dunnett’s post-hoc test).

Following EGF treatment, a significant increase in the number of acidic vesicles, as indicated by LysoTracker Red (LTR) fluorescence, was observed as early as 15 min post-treatment (Fig. 1A). However, starting from 2 h after EGF exposure, the count of acidic lysosomes returned to basal levels, suggesting that EGF-induced lysosomal upregulation is a transient phenomenon (Fig. 1A).

We then explored the possibility of EGF triggering lysosomal biogenesis. At 15 min post-treatment, we detected an induction of lysosomal-associated membrane protein 1 (LAMP-1), with more pronounced changes becoming evident at the 30-min mark (Fig. 1B). This effect persisted for up to 4 h after EGF exposure (Fig. 1B). Additionally, the levels of cathepsin B (CTSB), a representative lysosomal protease, exhibited rapid increments in response to EGF treatment, along with their swift activation into their mature forms (Fig. 1C). We also confirmed heightened CTSB activity (Fig. 1D). In summary, our findings suggest that the rapid endocytosis of EGFR triggered by EGF results in a prompt increase in the population of acidic lysosomal vesicles. Moreover, this process induces the synthesis of proteins necessary for lysosomal function, leading to a significant enhancement in both the quantity and functionality of lysosomes.

### EGF treatment attenuates zinc-induced neuronal death in mouse cerebrocortical cultures

**Fig. 2:**
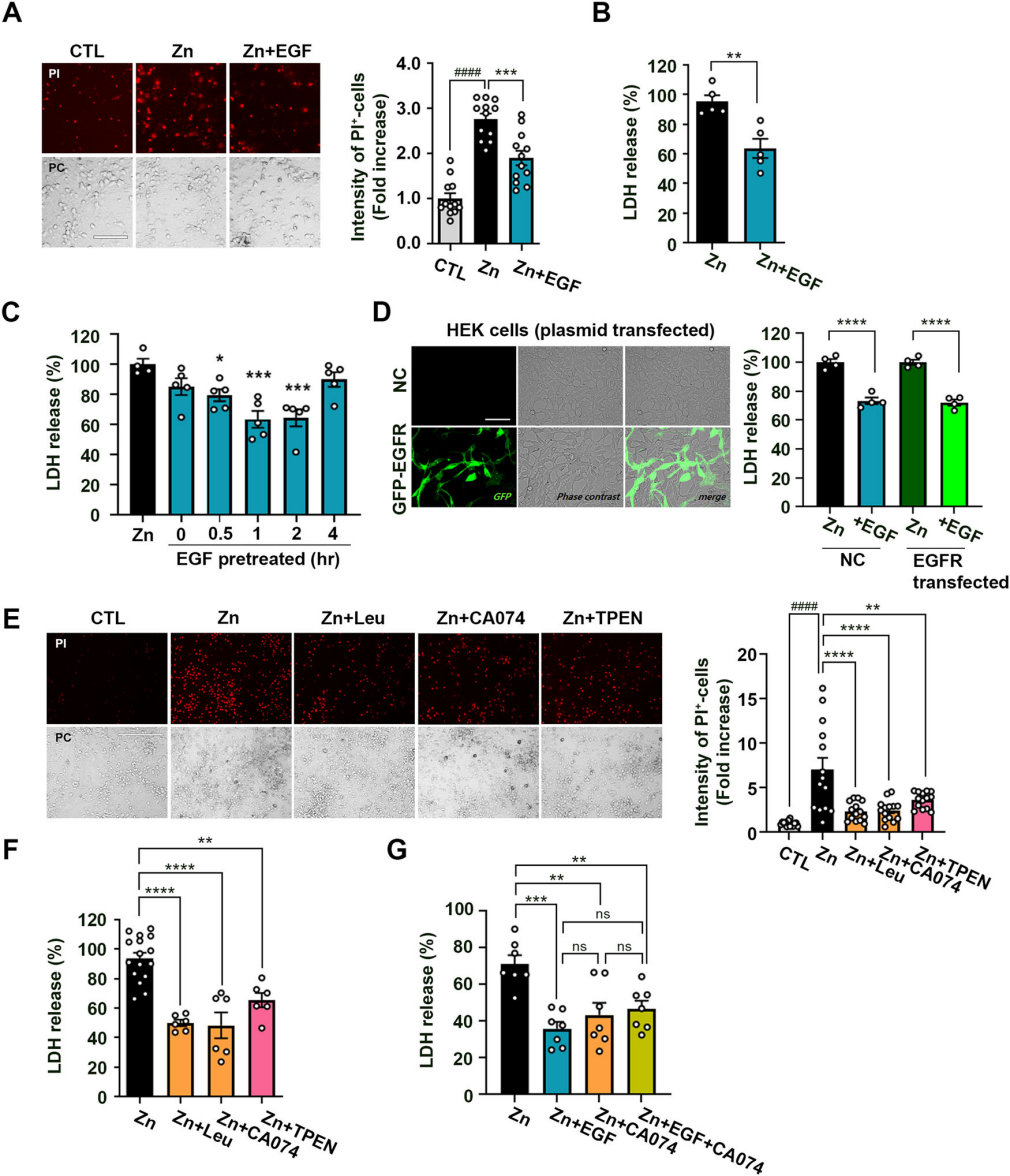
Attenuation of zinc-induced neuronal death by EGF treatment in mouse cerebrocortical cultures. **A** Microscopic images (left) and a quantitative graph (right) depict propidium iodide (PI)-stained damaged neuronal cells at 20 hours after exposure to 40 µM zinc, with or without EGF (100 ng/ml). Scale bar: 100 µm. The bar graph indicates the fluorescence intensity of PI measured using the Image J program (mean ± SEM, *n* = 12 different fields taken from ≥4 independent biological replicate experiments, ^####^*p* < 0.0001 for control (CTL), and ***p < 0.001 for zinc exposure, analyzed using ANOVA with Dunnett’s post-hoc test). **B** Bars represent LDH release at 20 h after exposure to zinc (40 µM) with or without EGF (100 ng/ml) (mean ± SEM, *n* = 5 independent biological replicate experiments, **p < 0.01; two-tailed Student’s *t*-test). **C** Bars represent LDH release at 20 h after exposure to zinc (40 µM) with or without EGF (100 ng/ml) pretreatment for the indicated time points (mean ± SEM, *n* = 4 independent biological replicate experiments, *p < 0.05 or ***p < 0.001 for zinc ex*p*osure, analyzed using ANOVA with Dunnett’s post-hoc test). **D** Microscopic images (left) showing GFP fluorescence following transient transfection with pCMV3-untagged negative control vector (NC) or GFP-EGFR tagged plasmid. Scale bar: 75 µm. The right bar graph represents LDH release at 18 h after exposure to zinc (80 µM) in empty vector (NC) or EGFR overexpressing HEK cells (mean ± SEM, *n* = 4 independent biological replicate experiments, ****p < 0.0001; two-tailed Student’s *t*-test). **E** Microscopic images (left) and a quantitative graph (right) show PI-stained damaged neuronal cells at 20 hours after exposure to 40 µM zinc, with or without leupeptin (Leu; 100 µM), CA074 (20 µM), or TPEN (1 µM). Scale bar: 200 µm. Bars represent the fluorescence intensity of PI (mean ± SEM, *n* = 14 different fields taken from ≥4 independent biological replicate experiments, ^####^*p* < 0.0001 for control (CTL), and **p < 0.01 or ****p < 0.0001 for zinc exposure, analyzed using ANOVA with Dunnett’s *p*ost-hoc test). **F** Bars indicate LDH release at 20 h after exposure to zinc (40 µM) with or without leupeptin (Leu; 100 µM), CA074 (20 µM), or TPEN (1 µM) (mean ± SEM, *n* = 6 or 16 taken from ≥4 independent biological replicate experiments, **p < 0.01 or ****p < 0.0001 for zinc exposure, analyzed using ANOVA with Dunnett’s post-hoc test). **G** Bars represent LDH release at 20 h after exposure to zinc (40 µM) with or without EGF (100 ng/ml), CA074 (20 µM), or EGF plus CA074 (mean ± SEM, *n* = 7 independent biological replicate experiments, **p < 0.01 or ***p < 0.001 for zinc exposure, analyzed using ANOVA with Dunnett’s post-hoc test). No significant difference (ns) was observed among treatment with EGF, CA074, or EGF plus CA074.

After establishing the role of EGF in enhancing functional lysosomes, we aimed to determine whether EGF could mitigate zinc-induced neuronal death in mouse cerebrocortical cultures. Exposure to zinc resulted in an increase in propidium iodide (PI)-stained damaged cells and LDH release into media, both of which were alleviated by EGF treatment (Fig. 2A, B).

To determine whether the neuroprotective effect of EGF was due to lysosomal upregulation or to EGFR downregulation and recycling, we varied the pre-treatment times of EGF and observed the effect on zinc-induced neurotoxicity. The protective effect began at 30 minutes, increased gradually, peaked at 1–2 h, and was completely absent with pre-treatment longer than 4 hours (Fig. 2C). This correlates with the observation that LAMP-1 expression began to decrease slightly at 4 hours (Fig. 1B), and the increase in mature cathepsin B had almost returned to baseline at 4 h (Fig. 1C). Additionally, the LTR signal began to decline after 1 h and returned to control levels within 4 h after EGF treatment (Fig. 1A). Meanwhile, EGFR expression sharply decreased 15 min after EGF treatment and did not recover by 4 h (Fig. 1B). Therefore, the neuroprotective effect of EGF appears to be due to lysosome upregulation itself rather than receptor-mediated signaling. To confirm this, we overexpressed EGFR in HEK293 cells and checked whether zinc toxicity was further reduced. However, the EGF-induced reduction in zinc toxicity did not increase with EGFR overexpression (Fig. 2D), indicating that the protective effect of EGF is independent of receptor expression levels. Taken together, these results suggest that the neuroprotective effect of EGF is related to the quantitative increase in lysosomes rather than the EGF-EGFR mediated signaling pathway.

Exposure to lethal dose of zinc within the culture media resulted in an elevation of cytosolic labile zinc levels, with subsequent infiltration into intracellular organelles, notably lysosomes [24]. The rapid and excessive increase in free zinc concentration within lysosomes led to the rupture of the lysosomal membrane, a phenomenon known as lysosomal membrane permeabilization (LMP). This process, in turn, resulted in the release of lysosomal proteases, including CTSB, triggering further detrimental effects such as caspase-3 activation and inflammasome formation [42, 43]. To confirm the involvement of LMP in zinc-induced neuronal death, we employed pretreatment with leupeptin (a cysteine protease inhibitor) or CA-074 methyl ester (CA074, a cathepsin B inhibitor). Remarkably, leupeptin, CA074 as well as tetrakis(2-pyridylmethyl)ethylenediamine (TPEN), a zinc chelator, substantially alleviated zinc-induced neuronal death (Fig. 2E, F). Furthermore, when EGF was co-administered with a CTSB inhibitor, it failed to provide additional neuroprotective effects, indicating a common protective mechanism mediated by EGF and lysosomal proteases (Fig. 2G). Thus, EGF significantly reduced zinc-induced neuronal death by inhibiting LMP and subsequent protease-mediated actions within the cytosol.

### EGF treatment prevents zinc-induced lysosomal membrane permeabilization (LMP) in mouse cerebrocortical cultures

**Fig. 3:**
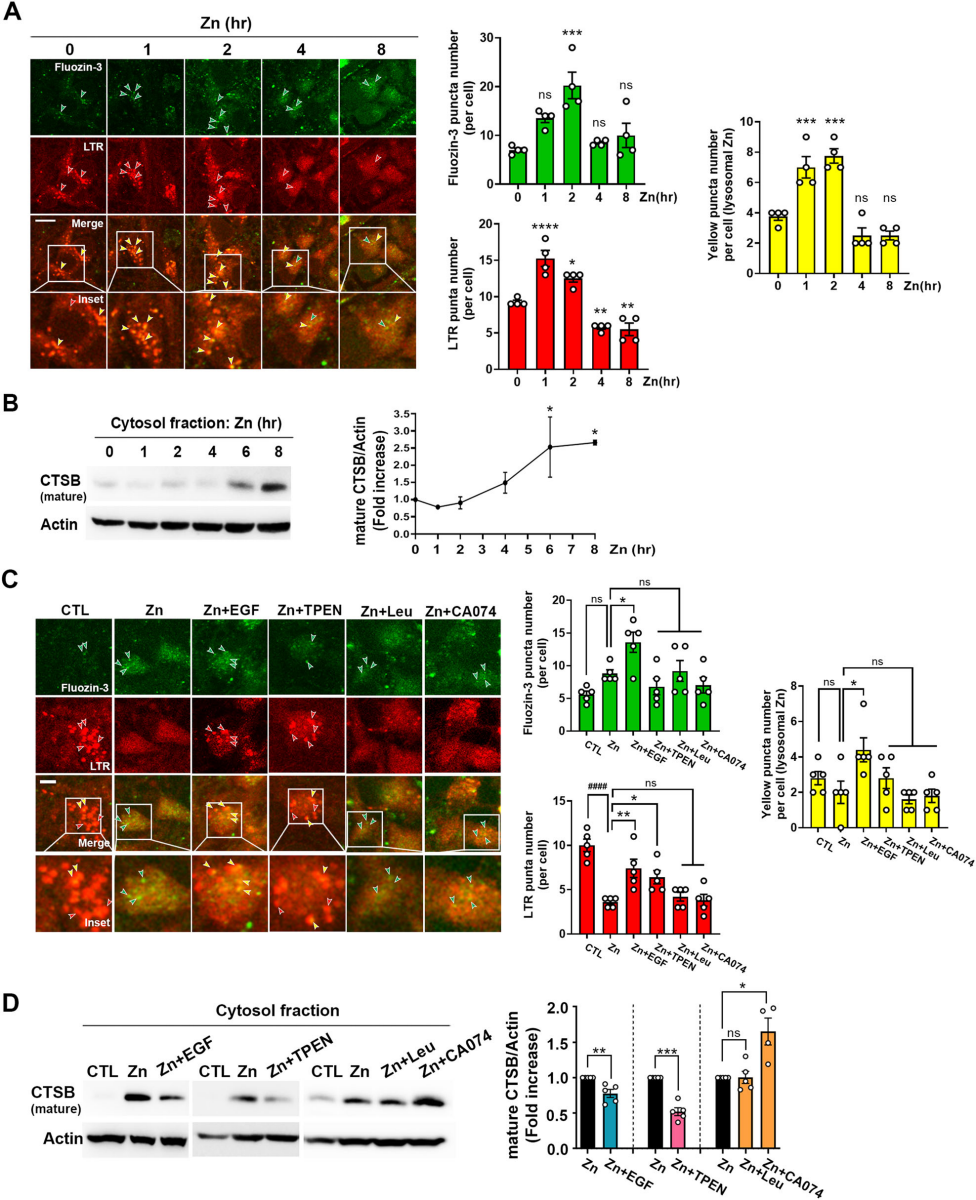
Reduction of zinc-induced lysosomal membrane permeabilization (LMP) and subsequent release of cathepsin B by EGF treatment. **A** Confocal microscopic images (left) and quantitative graphs (right) illustrate Fluozin-3 and LTR-stained neuronal cells over time following zinc treatment. Scale bar: 10 µm. The dotted box is presented at the bottom as enlarged images. Green, red or yellow arrowheads indicate zinc-containing vesicles, intact lysosomes, or zinc-containing lysosomes, respectively. The bars represent puncta number per cell (mean ± SEM, *n* = 4 different fields taken from independent biological replicate experiments, *p < 0.05, **p < 0.01, ***p < 0.001, ****p < 0.0001, or not significant (ns); compared with 0 h, analyzed using ANOVA with Dunnett’s post-hoc test). **B** Western blot analysis demonstrating the release of cathepsin B (CTSB) from lysosomes to the cytosol. Cytosolic proteins were extracted at the indicated time points after exposure to 40 µM zinc. The line graph presents the band intensity of mature CTSB to Actin over time (mean ± SEM, *n* = 4 independent biological replicate experiments, *p < 0.05; compared with 0-h, analyzed using ANOVA with Dunnett’s post-hoc test). **C** Confocal microscopic images (left) and quantitative graphs (right) depict Fluozin-3 and LTR-labeled neuronal cells at 4 h after 40 µM zinc treatment, with or without EGF (100 ng/ml), TPEN (1 µM), leupeptin (Leu; 100 µM), or CA074 (20 µM). Scale bar: 5 µm. The dotted box is presented at the bottom as enlarged images. Green, red or yellow arrowheads indicate zinc-containing vesicles, intact lysosomes, or zinc-containing lysosomes, respectively. The bars represent puncta number per cell (mean ± SEM, *n* = 5 different fields taken from ≥4 independent biological replicate experiments, ^####^*p* < 0.0001 for control (CTL), and *p < 0.05 or **p < 0.01 for zinc exposure, analyzed using ANOVA with Dunnett’s post-hoc test). **D** Western blot analysis showing cathepsin B (CTSB) release from lysosomes to the cytosol. Cytosolic proteins were obtained at 6 hours after exposure to 40 µM zinc, with or without EGF (100 ng/ml), TPEN (1 µM), leupeptin (Leu; 100 µM), or CA074 (20 µM). The bar graph represents the band density ratio of mature CTSB to Actin (mean ± SEM, *n* = 5 independent biological replicate experiments, **p < 0.01 or ***p < 0.001, analyzed using two-tailed Student’s *t*-test, and *p < 0.05 or not significant (ns), analyzed using ANOVA with Dunnett’s post-hoc test).

To observe LMP occurrence after zinc exposure and determine whether EGF could counteract it, we performed double staining with Fluozin-3 and LysoTracker Red (LTR). Our investigation revealed dynamic changes in cellular zinc distribution and lysosomal integrity. Initially, there was an increase in zinc-containing vesicles within the first 2 h, followed by a subsequent decline, coinciding with an increase in cytoplasmic zinc concentration (Fig. 3A, Fluozin-3 green color). Similarly, LTR-stained lysosomal vesicles showed a peak increase at 1–2 h post zinc exposure, gradually decreasing below control levels from 4 h onwards (Fig. 3A, LTR red color). Merged yellow puncta, representing zinc-containing lysosomes, emphasized this pattern. Notably, the increased presence of zinc-containing lysosomes at 1 or 2 h after zinc exposure was followed by their diminishment at 4 or 8 h, signifying the onset of LMP (Fig. 3A, merged yellow color).

The confirmation of zinc-induced LMP was achieved through western blot analysis of cathepsin B (CTSB) release into the cytosol. A significant increase in released CTSB levels was observed from 6 h after zinc treatment (Fig. 3B).

To evaluate the potential of EGF in mitigating zinc-induced LMP, we examined the effect of EGF on zinc-treated cultures. At 4 h after zinc treatment, there was only an increase in diffused cytosolic zinc, and intact lysosomes were notably diminished (Fig. 3C), consistent with Fig. 1A. However, EGF administration preserved intact lysosomes (indicated by red arrowheads) and zinc-containing lysosomes (depicted by yellow arrowheads) compared to cultures treated solely with zinc (Fig. 3C). This protective effect was accompanied by a reduction in the levels of CTSB release, as shown through western blot analysis (Fig. 3D left), emphasizing the mitigatory impact of EGF on zinc-induced LMP. Furthermore, we confirmed that LMP and CTSB release are induced by increased intracellular zinc, as TPEN, an intracellular zinc chelator, did not induce LMP or CTSB release (Fig. 3C, D). Considering the neuroprotective effects of leupeptin and CA074 against zinc-induced cytotoxicity (Fig. 2E–G), we examined their influence on zinc-induced LMP in mouse cerebrocortical cultures. However, the presence of lysosomal protease inhibitors, leupeptin or CA074, did not maintain the integrity of LTR-positive dots, resembling the conditions seen in cultures exposed solely to zinc (Fig. 3C). Additionally, leupeptin or CA074 was ineffective in reducing zinc-induced CTSB release into the cytosol (Fig. 3D). On the contrary, CA-074 seemed to augment zinc-induced CTSB release into cytosol, a result of heightened stability by inhibiting CTSB activity. These results suggest that while CTSB inhibitors may not directly impede LMP during zinc neurotoxicity, they may exert their protective effect against zinc-induced neurotoxicity by suppressing CTSB activity released from lysosomes to the cytosol following LMP.

### The protective effects of EGF against zinc-induced LMP and neuronal cell death are mediated by endocytosis and retrograde trafficking processes

Having established EGF’s capacity to enhance functional lysosomes and reduce LMP-associated neuronal death, we aimed to elucidate the significance of endocytic processes in facilitating EGF’s protective effects. The internalization of EGFR involves both clathrin-mediated endocytosis (CME) and non-clathrin endocytosis (NCE, specifically caveolin-mediated endocytosis), along with subsequent degradation processes [41, 44]. Our initial focus was on evaluating the potential of inhibiting CME and NCE pathways as strategies to counteract EGF’s protective influence on zinc-induced LMP and neuronal death.

**Fig. 4:**
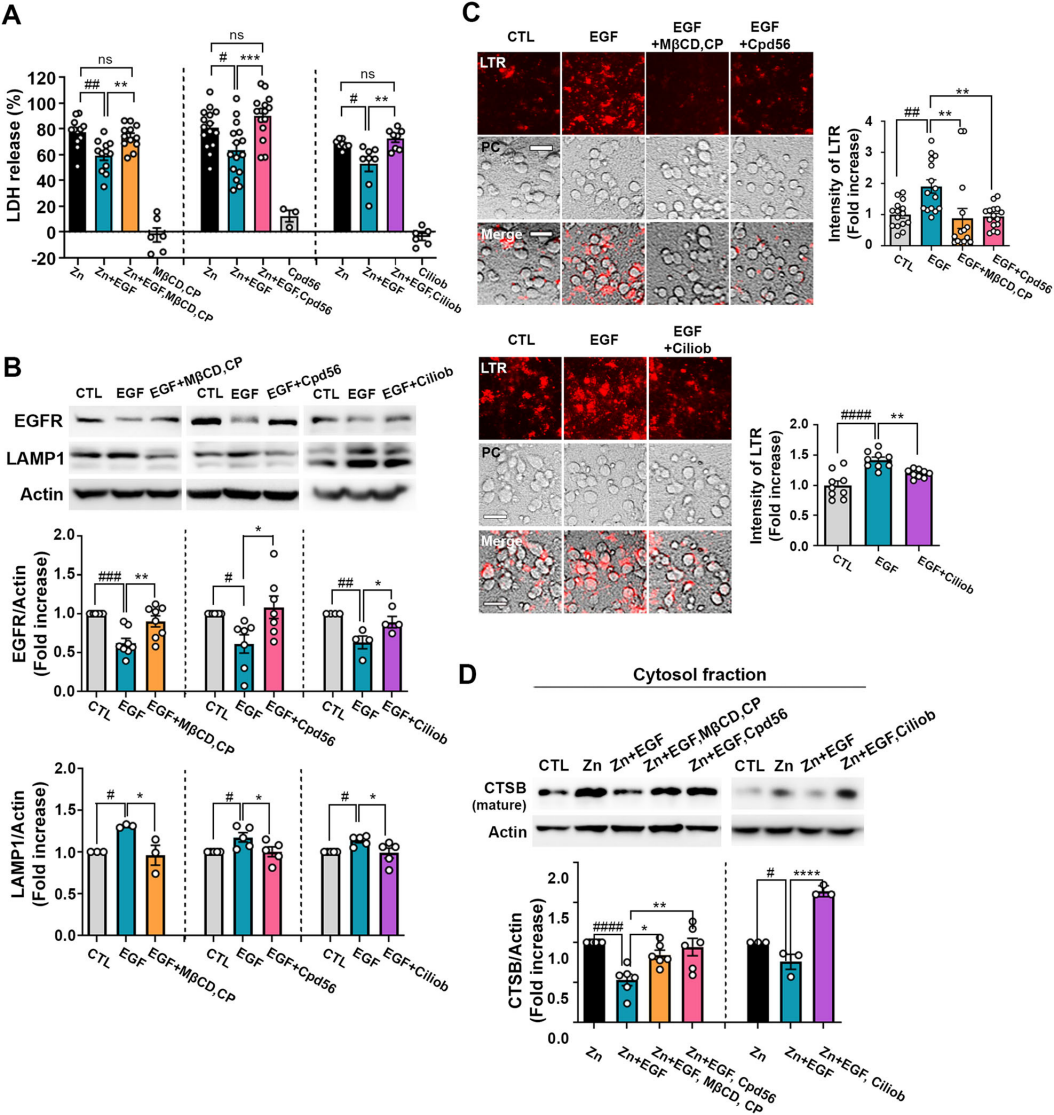
Endocytosis and retrograde trafficking mediate EGF’s protection against zinc-induced LMP and neuronal cell death in primary mouse cerebrocortical cultures. **A** Bars represent LDH release at 20 hours after exposure to zinc (40 µM), with or without EGF (100 ng/ml), methyl-beta-cyclodextrin (MβCD, 2 mM; caveolin-dependent endocytosis inhibitor), chlorpromazine (CP, 2 µM; clathrin-dependent endocytosis inhibitor), compound 56 (Cpd56, 1 µM; inhibitor of EGFR tyrosine kinase activity), or ciliobrevin A (ciliob, 500 nM; dynein inhibitor) (mean ± SEM, *n* = 3-15 taken from ≥3 independent biological replicate experiments, ^#^*p* < 0.05, ^##^*p* < 0.01, or not significant (ns) for zinc, and **p < 0.01 or ***p < 0.001 for Zn + EGF, analyzed using ANOVA with Dunnett’s post-hoc test). **B** Western blot analysis (upper) and quantitative graphs (lower) demonstrate the protein levels of EGFR and LAMP-1 at 0.5 hours after EGF (100 ng/ml) treatment with or without endocytosis inhibitors: MβCD (2 mM), CP (2 µM), Cpd56 (1 µM), or Ciliob (500 nM). The bar graphs indicate the band density ratio of EGFR or LAMP-1 to Actin (mean ± SEM, *n* = 4–8 independent biological replicate experiments, ^#^*p* < 0.05, ^##^*p* < 0.01, or ^###^*p* < 0.001 for control^,^ and *p < 0.05 or **p < 0.01 for EGF, analyzed by ANOVA using Dunnett’s post-hoc test). **C** Microscopic images (left) and quantitative graphs (right) show LTR-labeled neuronal cells at 1 h after EGF (100 ng/ml) treatment, with or without endocytosis inhibitors: MβCD (2 mM), CP (2 µM), Cpd56 (1 µM), or Ciliob (500 nM). Scale bar: 100 µm. The bar graphs represent LTR intensity measured using the Image J program (mean ± SEM, *n* = 9 or 15 different fields taken from ≥4 independent biological replicate experiments, ^##^*p* < 0.01 or ^####^*p* < 0.0001 for control, and **p < 0.01 for EGF, analyzed by ANOVA with Dunnett’s post-hoc test). **D** Western blot analysis demonstrates the release of cathepsin B (CTSB) from lysosomes to the cytosol. Cytosolic proteins were extracted at 6 h after exposure to 40 µM zinc with or without EGF (100 ng/ml), MβCD (2 mM), CP (2 µM), Cpd56 (1 µM), or Ciliob (500 nM). The lower bar graph presents the band intensity ratio of mature CTSB to Actin (mean ± SEM, *n* = 6 or 3 independent biological replicate experiments, ^#^*p* < 0.05, or ^####^*p* < 0.0001 for zinc, and *p < 0.05, **p < 0.01, or ****p < 0.0001 for Zn + EGF, analyzed by ANOVA using Dunnett’s post-hoc test).

Pretreatment with methyl-β-cyclodextrin (MβCD), a caveolin-dependent endocytosis inhibitor, and chlorpromazine (CP), a clathrin-dependent endocytosis blocker, before concurrent zinc and EGF treatment, effectively reversed the neuroprotective effect conferred by EGF against zinc-induced neurotoxicity (Fig. 4A left). We subsequently confirmed that the reversal effect of MβCD and CP was a result of inhibiting EGFR endocytosis. Notably, these agents, MβCD and CP, significantly preserved EGFR levels even after EGF treatment, and EGF-induced LAMP-1 expression returned to its control baseline (Fig. 4B left). MβCD and CP also led to a reduction in the number of acidified lysosomal vesicles (Fig. 4C). Furthermore, the improvement of CTSB release by EGF was significantly reversed by MβCD and CP (Fig. 4D). All these results underscore the contribution of CME and NCE to EGF-induced lysosomal enhancement and subsequent protection against zinc-induced LMP and neuronal death.

Subsequently, we investigated the impact of Compound 56 (Cpd56), a tyrosine kinase inhibitor targeting EGFR, on EGF-induced lysosomal upregulation and neuroprotection. Inhibiting EGFR’s tyrosine kinase activity with Cpd56 led to a reversal of EGF-mediated neuroprotection (Fig. 4A middle). We confirmed that Cpd56’s effect was mediated by the blockade of EGFR endocytosis, as demonstrated by the maintenance of EGFR (Fig. 4B middle). EGF-triggered LAMP-1 induction and the elevation in lysosomal vesicles were also reversed by Cpd56 (Fig. 4B, C). Finally, we observed that the blocking of zinc-induced CTSB release by EGF was effectively counteracted by the EGFR tyrosine kinase inhibitor (Fig. 4D).

Next, we assessed the role of retrograde trafficking in EGF-mediated neuroprotection by using ciliobrevin A (ciliob), a dynein-inhibiting chemical agent. Disrupting endosome maturation via retrograde transport inhibition with ciliobrevin A significantly reversed EGF-triggered neuroprotection against zinc toxicity (Fig. 4A right). Notably, ciliobrevin A also reversed EGF-triggered EGFR degradation and subsequent lysosomal formation (Fig. 4B, C). Ciliobrevin A also reversed the EGF-induced attenuation of CTSB release (Fig. 4D). Taken together, our findings underscore the pivotal role of EGF-triggered EGFR endocytosis in promoting lysosomal upregulation and providing neuroprotection against zinc-induced neurotoxicity.

### LAMP-1 overexpression also attenuates zinc-induced LMP and cell death in neuronal cultures and HEK cells

**Fig. 5:**
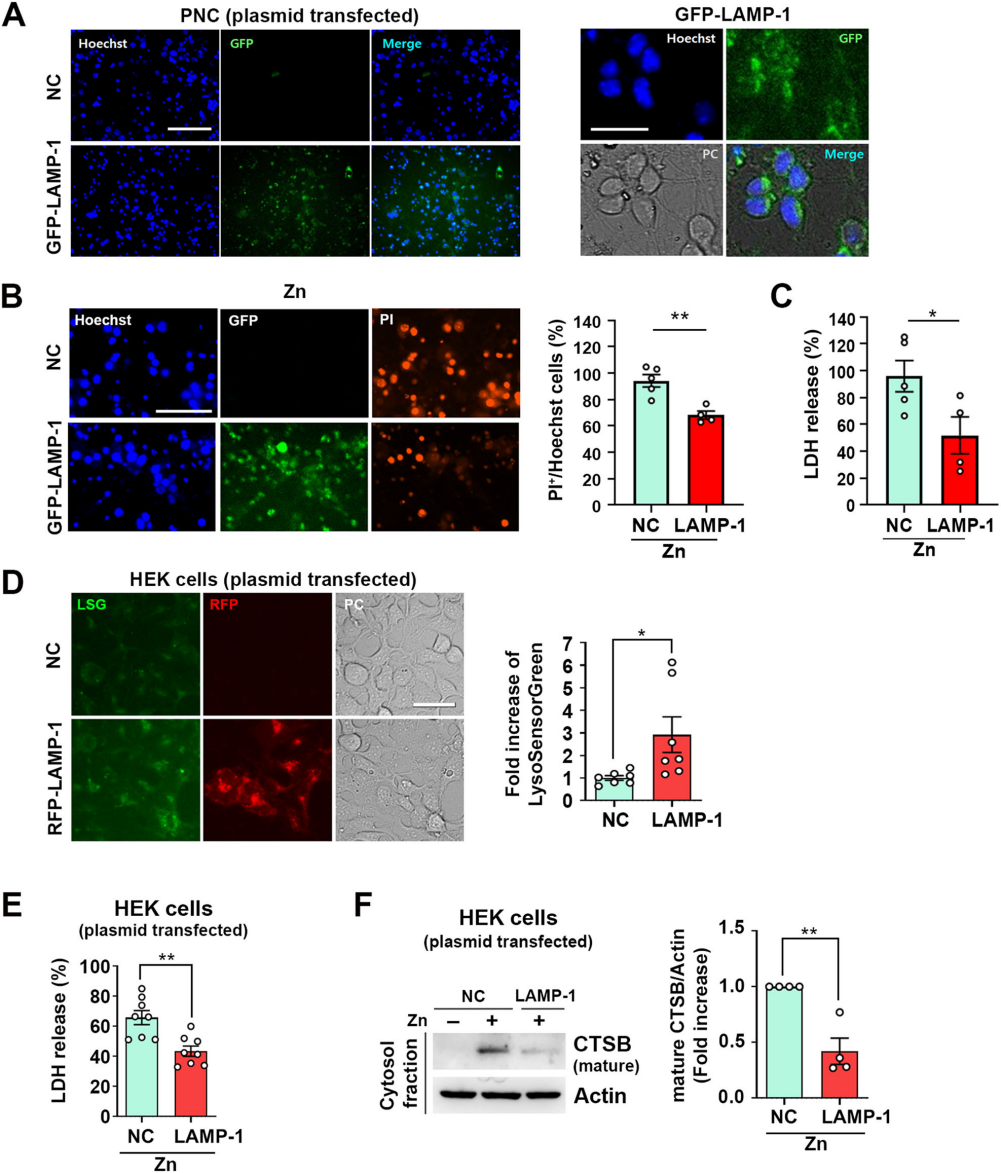
Attenuation of zinc-induced LMP and cell death in Primary cerebrocortical neuronal cells and HEK cells through LAMP-1 overexpression. **A** Microscopic images showing GFP fluorescence following transient transfection with either pCMV3-untagged negative control vector (NC) or GFP-LAMP-1 tagged plasmid in primary neuronal cells (PNC). More than 50% neurons expressed GFP-LAMP1 in these cultures. Scale bar: 75 µm (left panel), and 20 µm (right panel). **B** Microscopic images (left) and a quantitative graph (right) showing PI-stained damaged neuronal cells 18 hours after exposure to 40 µM zinc in either empty vector (NC) or LAMP-1 overexpressing neuronal cells. Scale bar: 50 µm. The bar graph indicates the ratio of PI-positive cells compared to Hoechst counterstained cells, measured using the Image J program (mean ± SEM, *n* = 4–5 different fields taken from 3 independent biological replicate experiments, **p < 0.01; two-tailed Student’s *t*-test). **C** Bar graph showing LDH release 18 h after exposure to zinc (40 µM) in empty vector (NC) or LAMP-1 overexpressing neuronal cells (mean ± SEM, *n* = 4–5 taken from ≥ 3 independent biological replicate experiments, *p < 0.05; two-tailed Student’s *t*-test). **D** Microscopic images (left) and a quantitative graph (right) showing Lysosensor Green (LSG) fluorescence following transient transfection with pCMV3-untagged negative control vector (NC) or RFP-LAMP-1 tagged plasmid. Scale bar: 50 µm. The bar graph represents LSG intensity using the Image J program (mean ± SEM, *n* = 7 different fields taken from ≥4 independent experiments, *p < 0.05; two-tailed Student’s *t*-test). **E** Bar graph depicts LDH release at 12 h after exposure to zinc (80 µM) in empty vector (NC) or LAMP-1 overexpressing HEK cells (mean ± SEM, *n* = 8 taken from ≥4 independent experiments, **p < 0.01; two-tailed Student’s *t*-test). **F** Western blot analysis demonstra*t*es the release of cathepsin B (CTSB) from lysosomes to the cytosol. Cytosolic proteins were extracted at 5 h with or without 80 µM zinc treatment in empty vector (NC) or LAMP-1 overexpressing HEK cells. The bar graph presents the band intensity ratio of mature CTSB to Actin (mean ± SEM, *n* = 4 independent experiments, **p < 0.01; two-tailed Student’s *t*-test).

Previously, we observed that EGF-triggered lysosomal upregulation effectively attenuated zinc-induced LMP and cell death. Here, we investigated whether genetic overexpression of LAMP-1 could replicate these protective effects. Initially, we transiently transfected a GFP-LAMP-1 plasmid into primary mouse cerebrocortical near-pure neuronal cultures, achieving a transfection efficiency of 52.11 ± 19.5% (Fig. 5A). LAMP-1 overexpression provided significant neuroprotection against zinc neurotoxicity, as indicated by PI staining and LDH assay (Fig. 5B, C). However, due to the limited transfection efficiency in primary mouse cerebrocortical neurons, we turned to human embryonic kidney (HEK) cells for further experiments. In HEK cells, overexpression of RFP-LAMP-1 markedly increased the population of RFP-positive organelles, indicative of lysosomes. We confirmed that LAMP-1 plasmid transfection not only elevated lysosome numbers but also enhanced lysosomal acidification, as indicated by lysosensor green (LSG) staining (Fig. 5D). Remarkably, elevated LAMP-1 expression substantially reduced zinc-induced toxicity (Fig. 5E) and prevented zinc-induced LMP and CTSB release into the cytosol in HEK cells (Fig. 5F). These findings collectively indicate that LAMP-1 overexpression enhances both the quantity and activity of lysosomes, thereby contributing to the regulation of intracellular zinc homeostasis.

### EGF does not protect against glutamate-induced excitotoxicity and STSP-induced apoptosis

**Fig. 6:**
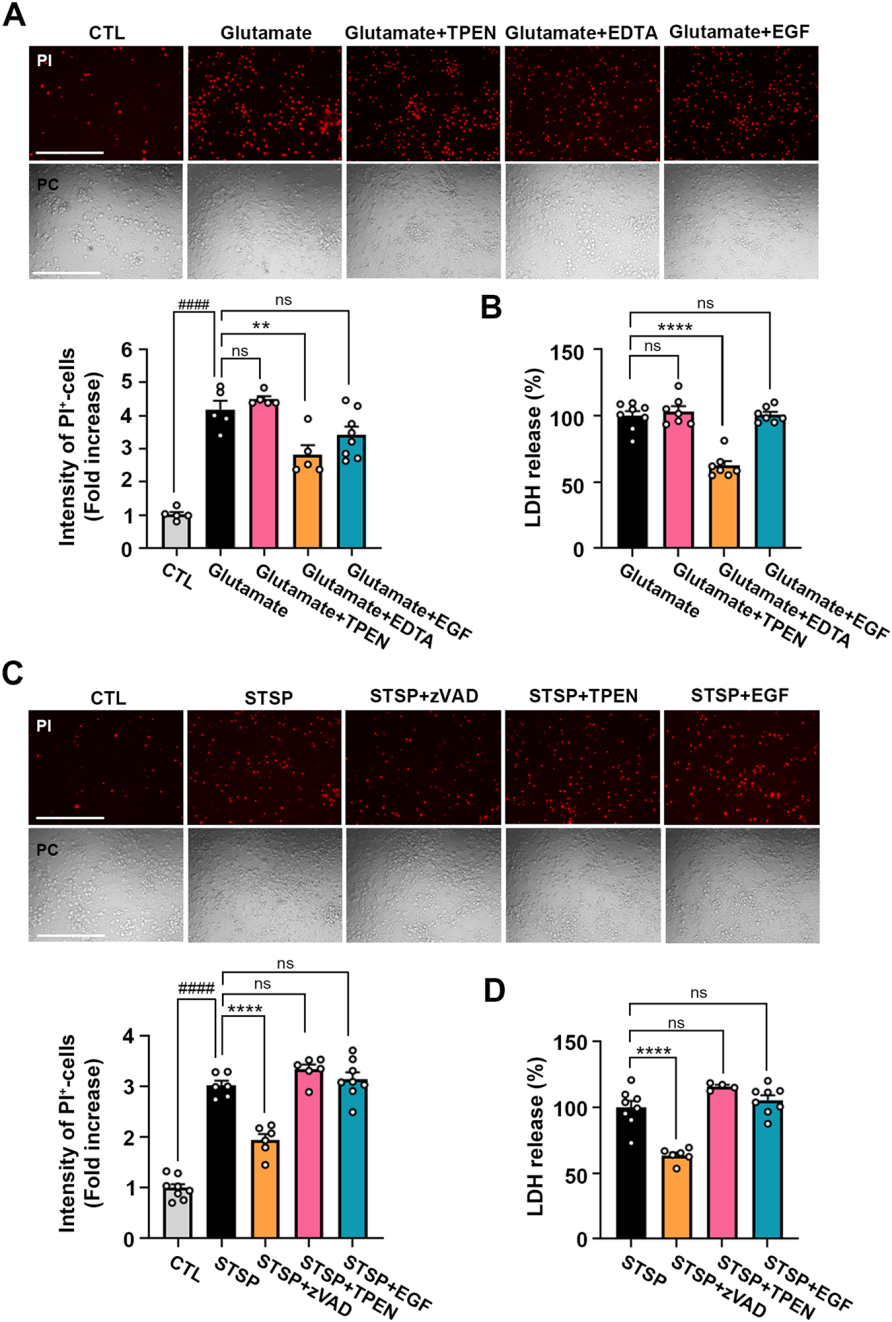
Ineffectiveness of EGF in protecting against glutamate-induced excitotoxicity and STSP-induced apoptosis in mouse cerebrocortical cultures. **A** Microscopic images (upper) and a quantitative graph (lower) show PI-stained damaged neuronal cells after exposure to glutamate (150 µM, 12 h) with or without TPEN (1 µM), EDTA (1 mM) or EGF (100 ng/ml) in mouse cerebrocortical cultures. Scale bar: 200 µm. The bar graph indicates the fluorescence intensity of PI measured using the Image J program (mean ± SEM, *n* = 8 or 6 different fields taken from ≥4 independent biological replicate experiments, ^####^*p* < 0.0001 for control, and **p < 0.01 or not significant (ns) for glutamate, analyzed using ANOVA with Dunnett’s post-hoc test). **B** Bars indicate LDH release from damaged neuronal cells after exposure to glutamate (150 µM, 12 h) with or without TPEN (1 µM), EDTA (1 mM) or EGF (100 ng/ml) in mouse cerebrocortical cultures (mean ± SEM, *n* = 8 or 7 taken from ≥3 independent biological replicate experiments, ****p < 0.0001 or not significant (ns) for glutamate, analyzed using ANOVA with Dunnett’s post-hoc test). **C** Microscopic images (upper) and a quantitative graph (lower) show PI-stained damaged neuronal cells after exposure to staurosporine (STSP, 200 nM, 24 h) with or without zVAD (100 µM), TPEN (1 µM) or EGF (100 ng/ml) in mouse cerebrocortical cultures. Scale bar: 200 µm. The bar graph indicates the fluorescence intensity of PI (mean ± SEM, *n* = 6 ~ 8 different fields taken from ≥4 independent biological replicate experiments, ^####^*p* < 0.0001 for control, and ****p < 0.0001 or not significant (ns) for STSP, analyzed using ANOVA with Dunnett’s post-hoc test). **D** Bars indicate LDH release from damaged neuronal cells after exposure to STSP (200 nM, 24 h) with or without zVAD (100 µM), TPEN (1 µM) or EGF (100 ng/ml) in mouse cerebrocortical cultures (mean ± SEM, *n* = 5–8 taken from ≥4 independent biological replicate experiments, ****p < 0.0001 or not significant (ns) for STSP, analyzed using ANOVA with Dunnett’s post-hoc test).

Zinc-induced neuronal death plays a crucial role in neuronal loss in the cortex and hippocampus following acute brain injuries such as stroke, trauma, and epilepsy [4, 10–12, 45]. Since excess calcium influx and excitotoxicity are also significant contributors to neuronal death in these conditions [46], we investigated whether EGF-induced lysosome upregulation could reduce calcium-mediated toxicity. Initially, we observed that neuronal death induced by glutamate was not inhibited by the zinc chelator TPEN, but significantly decreased by the calcium chelator EDTA (Fig. 6A, B). In addition, EGF did not exhibit a reduction in calcium-mediated excitotoxicity (Fig. 6A, B).

CTSB released into the cytoplasm after LMP can activate caspase-3 and contribute to cell death in the form of apoptosis [43, 47] Therefore, we further investigated whether lysosomal upregulation induced by EGF treatment could reduce staurosporine (STSP)-induced apoptosis. STSP-induced neuronal death was effectively inhibited by the pan-caspase inhibitor zVAD but remained unaffected by the zinc chelator TPEN or EGF (Fig. 6C, D). In summary, EGF-mediated lysosomal upregulation appears to specifically target zinc-related neuronal cell death mechanisms.

### Oxidative damage mediated by zinc, following treatment with H_2_O_2_ or MPP^+^, is also reduced under conditions of lysosome upregulation

**Fig. 7:**
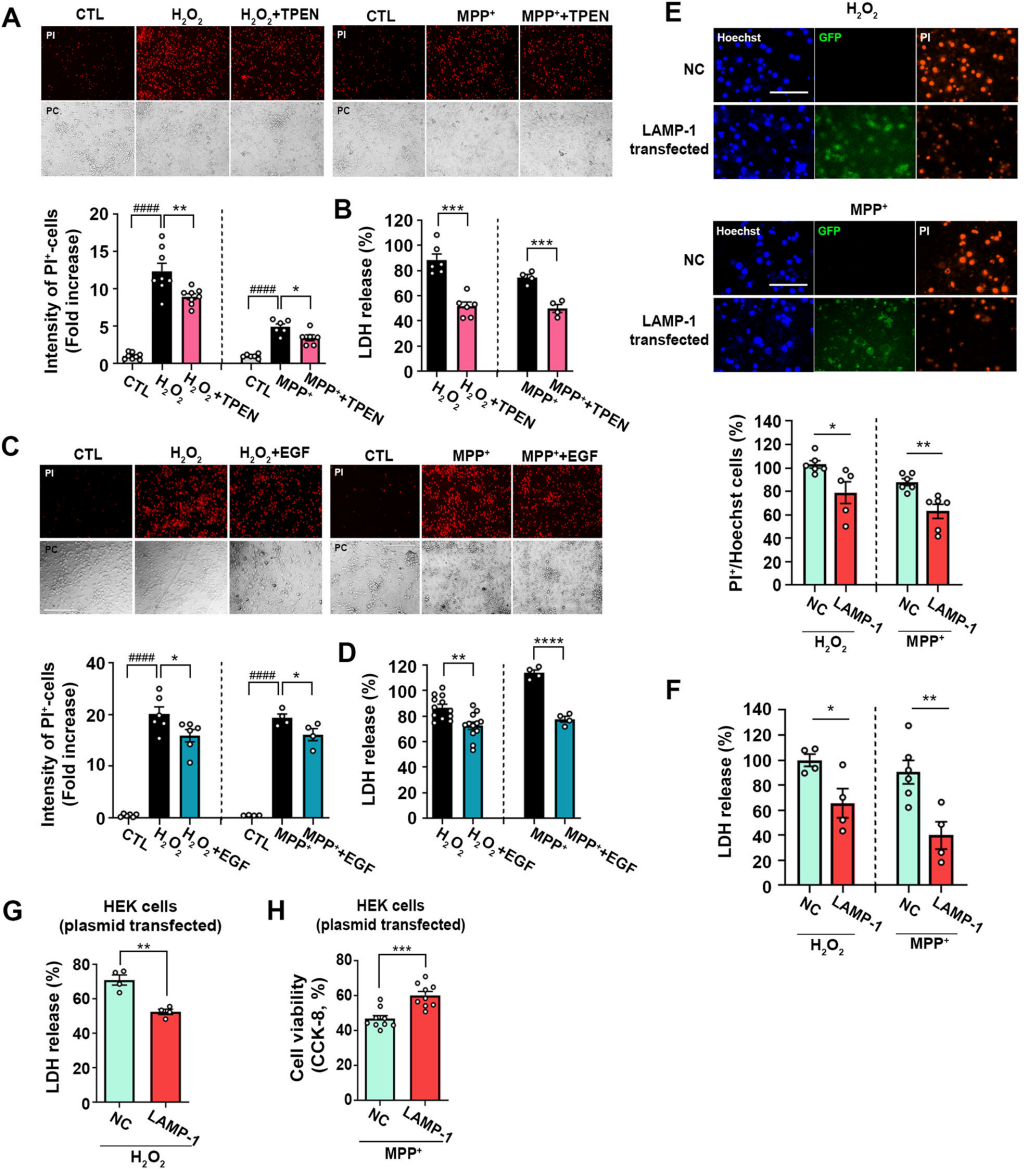
Alleviation of oxidative damage by H_2_O_2_ or MPP^+^ in lysosome-upregulated conditions. **A** Microscopic images (upper) and a quantitative graph (lower) show PI-stained damaged neuronal cells after exposure to H_2_O_2_ (140 µM, 5 h) or MPP^+^ (300 µM, 24 h) with or without TPEN (1 µM) in mouse cerebrocortical cultures. Scale bar: 200 µm. The bar graph indicates the fluorescence intensity of PI measured using the Image J program (mean ± SEM, *n* = 8 or 6 different fields taken from ≥4 independent biological replicate experiments, ^####^*p* < 0.0001 for control, **p < 0.01 for H_2_O_2_, and *p < 0.05 for MPP^+^, analyzed using ANOVA with Dunnett’s post-hoc test). **B** Bars indicate LDH release from damaged neuronal cells after exposure to H_2_O_2_ (140 µM, 5 h) or MPP^+^ (300 µM, 24 h) with or without TPEN (1 µM) in mouse cerebrocortical cultures (mean ± SEM, *n* = 6 or 4 taken from ≥4 independent biological replicate experiments, ***p < 0.001; two-tailed Student’s *t*-test). **C** Microscopic images (upper) and a quantitative graph (lower) show PI-stained damaged neuronal cells after exposure to H_2_O_2_ (140 µM, 5 h) or MPP^+^ (300 µM, 24 h) with or without EGF (100 ng/ml) in mouse cerebrocortical cultures. Scale bar: 200 µm. The bar graph indicates the fluorescence intensity of PI (mean ± SEM, *n* = 6 or 4 different fields taken from ≥4 independent biological replicate experiments, ^####^*p* < 0.0001 for control, and *p < 0.05 for H_2_O_2_ or MPP^+^, analyzed using ANOVA with Dunnett’s post-hoc test). **D** Bars indicate LDH release from damaged neuronal cells after exposure to H_2_O_2_ (140 µM, 5 h) or MPP^+^ (300 µM, 24 h) with or without EGF (100 ng/ml) in mouse cerebrocortical cultures (mean ± SEM, *n* = 12 or 4 taken from ≥4 independent biological replicate experiments, **p < 0.01 or ****p < 0.0001; two-tailed Student’s *t*-test). **E** Microsco*p*ic images (upper) and a quantitative graph (lower) showing PI-stained damaged neuronal cells after exposure to H_2_O_2_ (140 µM, 5 h) or MPP^+^ (300 µM, 24 h) in empty vector (NC) or LAMP-1 overexpressing neuronal cells. Scale bar: 50 µm. The bar graph indicates the ratio of PI-positive cells compared to Hoechst counterstained cells, measured using the Image J program (mean ± SEM, *n* = 5–6 different fields taken from 3 independent biological replicate experiments, *p < 0.05 for H_2_O_2_ and **p < 0.01 for MPP^+^, analyzed using two-tailed Student’s *t*-test). **F** Bar graph showing LDH release after exposure to H_2_O_2_ (140 µM, 5 h) or MPP^+^ (300 µM, 24 h) in empty vector (NC) or LAMP-1 overexpressing neuronal cells (mean ± SEM, *n* = 4–6 taken from ≥3 independent biological replicate experiments, *p < 0.05 for H_2_O_2_ and **p < 0.01 for MPP^+^, analyzed using two-tailed Student’s *t*-test). **G** Bars indicate LDH release from damaged HEK cells at 12 h after exposure to H_2_O_2_ (280 µM) in empty vector (NC) or LAMP-1 overexpressing HEK cells (mean ± SEM, *n* = 4, **p < 0.01; two-tailed Student’s *t*-test). **H** Bars indicate cell viability measured using CCK-8 viability assay at 50 h after exposure to MPP^+^ (3 mM) in empty vector (NC) or LAMP-1 overexpressing HEK cells (mean ± SEM, *n* = 9, ***p < 0.001; two-tailed Student’s *t*-test).

Numerous studies have emphasized the accumulation of zinc within lysosomes, leading to LMP upon exposure to hydrogen peroxide (H_2_O_2_) in cerebrocortical and hippocampal neuronal cultures [24]. Additionally, the complex I inhibitor, 1-methyl-4-phenylpyridinium ion (MPP^+^), has been shown to induce intracellular zinc accumulation both in vitro and in vivo. Furthermore, lysosomal breakdown has been observed in dopaminergic neuronal cells exposed to MPP^+^ [38]. Given these findings, we investigated whether EGF could confer protective effects against other types of oxidative stress, such as H_2_O_2_ or MPP^+^. Before assessing the impact of EGF, we confirmed that cell death induced by H_2_O_2_ and MPP^+^ could be prevented by the zinc chelator TPEN in mouse cerebrocortical cultures (Fig. 7A, B), thus confirming the role of zinc in mediating neuronal death. Subsequently, we observed that EGF effectively prevented H_2_O_2_ and MPP^+^-induced neuronal death (Fig. 7C, D). Notably, the overexpression of LAMP-1 in primary mouse cerebrocortical neuronal cultures significantly suppressed H_2_O_2_ or MPP^+^-induced neurotoxicity, as indicated by PI staining and LDH assays (Fig. 7E, F). We then confirmed the protective effects of LAMP-1 overexpression against H_2_O_2_ or MPP^+^-induced cytotoxicity in HEK cells. LAMP-1 overexpression markedly attenuated these toxicities (Fig. 7G, H), further reinforcing the applicability of our findings to diverse forms of neurotoxicity arising from intracellular zinc elevation and subsequent LMP.

## Discussion

In our study, we delved into the crucial role of lysosomes in guarding against zinc-mediated neurotoxicity, a significant contributor to acute brain injuries. Firstly, we observed a rapid increase in the number of lysosomal vesicles and the promotion of lysosomal biogenesis triggered by EGF. This augmentation, induced by EGF, effectively shielded against zinc-induced LMP and subsequent neuronal death. We confirmed that EGF’s protective effects were mediated through clathrin- and caveolin-mediated endocytosis pathways, in conjunction with retrograde trafficking. Remarkably, the overexpression of LAMP-1 replicated EGF’s protective effects in neurons and HEK cells. Also noted was EGF-induced lysosomal enhancement extending protection to other oxidative stress associated with intracellular zinc release. These findings highlight the intricate interplay between EGF-triggered EGFR endocytosis, lysosomal upregulation, enhanced regulation of zinc homeostasis, and the alleviation of zinc-induced neurotoxicity.

EGFR, the first discovered member of the receptor tyrosine kinase superfamily, plays a crucial role in the growth, differentiation, maintenance, and repair of various tissues and organs [48]. In the nervous system, research has primarily focused on neurotrophins such as NGF and BDNF and their receptors, leaving the role of EGFR relatively underexplored. However, EGFR is expressed in both the central and peripheral nervous systems [49, 50], and its trafficking and signaling are known to be involved in the onset and progression of neurodegenerative diseases and acute ischemic brain injury. In Parkinson’s disease (PD), reduced levels of EGF and EGFR expression have been observed in the postmortem brains of patients [51]. Additionally, dopaminergic lesions in animal models of PD lead to reduced EGF levels and EGFR activity [51]. Changes in EGFR endolysosomal trafficking have been reported to induce damage to dopaminergic neurons and potentially lead to PD [52]. Particularly in brain ischemic injury, the mechanisms of EGFR have been more thoroughly investigated. For instance, in a middle cerebral artery occlusion (MCAO) mouse model, intracerebroventricular injection of EGF has been shown to reduce the expression of Neutrophil gelatinase-associated lipocalin (NGAL), a marker of ischemic stroke, through the JAK2/STAT3 pathway, consequently decreasing brain infarct volume [53]. Additionally, in vivo global brain ischemia rat model and in vitro oxygen glucose deprivation (OGD) mouse model have demonstrated that endogenous tissue-type plasminogen activator (tPA) increases following ischemic damage, contributing to reduced neuronal damage via EGFR receptors [54]. While other research groups have focused on the signaling aspects of EGFR, we are the first to demonstrate that EGFR endocytosis can reduce zinc-mediated neurotoxicity through a quantitative increase in lysosomes. Therefore, studying neuroprotective mechanisms centered on EGFR is expected to contribute to understanding the mechanisms of ischemic brain injury and developing therapeutic strategies to control it.

Here, our study demonstrated that EGFR endocytosis triggered by EGF leads to a transient escalation in lysosomal vesicles characterized by a lower pH. This surge is accompanied with increased expressions of both LAMP-1, a representative lysosomal marker protein, and CTSB, a prototypical lysosomal protease. Notably, while sustained elevation of LAMP-1 and inactive CTSB proform persist for up to 4 hours post-EGF treatment, the count of acidic vesicles returns to baseline after 2–4 h (Fig. 1A–C). Lysosomal biogenesis continues for up to 4 h, but a significant increase in acidic lysosomal vesicles begins to decrease after 2 h. Corresponding to this lysosomal dynamics, CTSB activity peaks at 1 h post-EGF treatment, diminishes, and peak again at 4 hours (Fig. 1D). In essence, these CTSB activity fluctuations post-EGF treatment represent a combined outcome from the initial surge in low pH vesicles and subsequent delayed, sustained rises in CTSB expression.

Additionally, we confirmed that EGF-induced lysosomal upregulation is not dependent on the activation of intracellular signaling cascade, but rather mediated by EGFR’s endocytosis process (Fig. 4). Firstly, we verified that the expression level of EGFR is not directly related to the suppression of zinc neurotoxicity. Following EGF treatment, the expression level of EGFR rapidly declined due to endocytosis and degradation starting at 15 minutes and did not recover even after 4 h. However, zinc neurotoxicity was only mitigated when cells were pretreated with EGF for 30 min to 2 h, and no protective effect was observed with pretreatment beyond 4 hours (Fig. 2C). Additionally, overexpressing EGFR in HEK cells did not further reduce zinc cytotoxicity (Fig. 2D), indicating no direct correlation between EGFR expression levels and cytotoxic protection. In contrast, blocking endocytosis and retrograde trafficking preserved intact EGFR without degradation, halting lysosomal upregulation. Consequently, inhibiting the endocytosis erased the neuroprotective effect against zinc-induced LMP and neuronal death. Furthermore, compound 56, an EGFR tyrosine kinase inhibitor, when co-treated with EGF, impeded EGFR endocytosis, thus nullifying lysosomal upregulation and the protective effects against zinc neurotoxicity. These results suggest that EGF’s neuroprotective effect against zinc neurotoxicity is due to the facilitation of endocytosis rather than activation of signaling cascade. Validating that endocytosis promotion effectively regulates cytosolic free zinc levels via lysosomal upregulation, inhibiting zinc-mediated neuronal cell death, could establish it as a therapeutic strategy for acute brain injury.

An important finding in EGF-induced lysosomal upregulation is the rapid increase in LAMP-1 expression within 15 min after EGF treatment, along with CTSB. Surprisingly, LAMP-1 overexpression alone augmented the count of low-pH lysosomes and reduced zinc-induced neurotoxicity. However, the mechanism driving the swift induction of LAMP-1 expression via endocytosis remains unclear. Transcription factor TF-EB is widely recognized for orchestrating lysosomal biogenesis, involving the activation of various lysosomal proteins like CTSB, cathepsin D, and v-ATPase subunits through dephosphorylation and nuclear translocation [23]. Despite the rapid increase in LAMP-1 and CTSB expression, we observed no significant alterations in TF-EB dephosphorylation or nuclear translocation (data not shown). During EGFR internalization, the number of early endosomal vesicles and late endosomal vesicles temporarily increases, which can result in a transient increase in lysosome numbers. As shown in Fig. 1A, the LTR signal returned to baseline after 2 h, suggesting that the quantitative increase in lysosomes might simply be a result of increased trafficking vesicles. However, this does not explain the sustained increase in LAMP-1 and CTSB expression. Further investigations are needed to elucidate how endocytosis promotion precisely triggers lysosomal biogenesis, particularly the mechanisms driving increased LAMP-1 and CTSB expressions.

Recent research has shed light on the impact of LAMP-1 overexpression on lysosomal function. To maintain a low pH inside vesicles, the action of v-ATPase is essential [55]. Jiang et al. demonstrated that when LAMP-1 or LAMP-2 binds to the TMEM175 channel in lysosomes, it forms a complex inhibiting TMEM175 function, thereby contributing to lysosomal acidification (Mol Cell, 2023) [56]. TMEM175 acts as a proton leak channel in acidic environments, releasing protons out of lysosomes, alongside v-ATPase, maintaining lysosomal pH balance. Increased LAMP-1 expression inhibits TMEM175 function, resulting in v-ATPase predominance, acidifying lysosomes, and creating an environment where lysosomal hydrolases can be actively functional. To comprehensively understand the role of LAMP-1, further research investigating how LAMP-1 promotes lysosomal functions is warranted.

Our observations highlight that increased intracellular free zinc, triggered by oxidative damage or excessive zinc intake, accelerates zinc entry into lysosomes, initiating sudden LMP. Following LMP, released lysosomal enzymes potentially induce cellular damage by degrading cytoplasmic proteins or cytoskeleton matrix [57–59]. Additionally, cytosolic release of CTSB may contribute to initiating an inflammatory response by promoting the assembly of the inflammasome—a complex comprising NLRP3, ACS, and pro-caspase-1 [60–62]. This provokes caspase-1 activation, leading to the cleavage of pro IL-1β and pro IL-18, subsequently releasing IL-1β and IL-18 into the extracellular space [63]. Furthermore, cytosolic release of CTSB might directly induce apoptotic cell death by cleaving caspase-3 [64]. Zinc-mediated neuronal cell death exhibits feature of both necrosis and apoptosis [8, 65], characterized by increased oxidative stress via nNOS, NADPH oxidase, etc., heightened caspase-3 activity facilitated by p75NTR and Egr-1 activation, and typical apoptotic traits including DNA fragmentation [11, 14, 15, 66, 67]. Consequently, the release of CTSB following LMP in zinc-mediated neuronal death is hypothesized to expedite the apoptotic process through caspase-3 activity. Given the unknown precise mechanism of CTSB in zinc-mediated neuronal death, further research is needed to determine if the neuroprotective mechanism of CTSB inhibition involves reducing cytosolic proteins or cytoskeletal degradation, suppressing inflammasome formation, or inhibiting caspase-3 activation.

Intracellular calcium elevation acts as a secondary messenger, typically maintained at low level of around ~100 nM [68]. During synaptic activity or cellular signaling, elevated calcium ions should be expelled from cytoplasm via energy-dependent transporter action [69]. Proteins like calbindin also contribute to intracellular calcium homeostasis [70]. In the case of zinc ions, typically at around 100 pM in the cytoplasm—much lower than cytoplasmic calcium concentration—can transiently surge to micromolar levels during brain ischemic damage [71, 72]. Ten known zinc transporters, ZnT1 to ZnT10, are present in organelles like lysosomes, ER, and synaptic vesicles, allowing zinc influx using proton gradients [3]. Studies indicated that as intracellular zinc levels rise, lysosomes uptake zinc ions, potentially causing LMP and subsequent cell death if excessive [33, 73]. The size of lysosomes is closely related to their function. Inhibition of v-ATPase with Bafilomycin A1 results in alkalinization of lysosomal pH and an increase in lysosome size compared to the control group [74, 75]. Enlarged lysosomes can also be observed in brain tissues of patients or animal models with diseases such as neuronal ceroid lipofuscinosis (NCL), caused by genetic defects leading to reduced lysosomal function and accumulation of lipofuscin, or proteinopathies like PD, Alzheimer’s disease, and Huntington’s disease, where specific protein aggregates accumulate [76]. However, the detailed mechanisms of how lysosomes maintain an appropriate size are not well understood. When cytoplasmic zinc increases, it rapidly moves into lysosomes, causing them to enlarge. As more zinc continues to enter the lysosomes, they gradually increase in size until, at a certain point, the lysosomal membrane bursts, leading to lysosomal membrane permeabilization (LMP). If there are more lysosomes, the influx of zinc is distributed, reducing the frequency of LMP and consequently decreasing neurotoxicity. Our study validated lysosomes as critical intracellular organelles regulating zinc levels. Augmenting lysosomes via facilitated endocytosis enhances zinc homeostasis, reducing zinc-mediated neuronal cell death. Besides from calcium, ER and mitochondria also uptake zinc ions to regulate cytoplasmic zinc levels [77, 78]. However, given the significant difference in concentrations—cytoplasmic calcium being approximately 10^3^ times higher than zinc—segregation of representative intracellular organelles regulating calcium and zinc seems advantageous. Lysosomes likely act as representative intracellular organelles in zinc homeostasis regulation. Furthermore, since an increase in reactive oxygen species (ROS) triggers zinc release from zinc-bound proteins, an elevation in zinc could be a cause in ROS-induced neuronal death. Hence, understanding lysosomal quantitative control mechanisms might alleviate neuronal cell death induced by ROS as well as intracellular zinc increase. Research on lysosomal upregulation mechanisms could be crucial in preventing and treating neurological disorders such as stroke, epilepsy, traumatic brain injury—conditions involving increased ROS and zinc levels—underscoring the importance of comprehending lysosomal regulation.

Our findings indicate that EGF treatment attenuated neuronal death caused by the mitochondrial toxicant MPP^+^. Mitochondrial impairment stands as a significant factor in PD [79]. Although our study observed LMP induction by MPP^+^ toxicity solely in a cellular model, in the progression of PD, recurrent LMP gradually leads to lysosome deficiency. Therefore, exploring strategies to inhibit LMP could not only attenuate acute neuronal death but also tackle neurodegenerative diseases such as PD. Recurrent LMP gradually leads to lysosome deficiency, impeding the clearance of α-synuclein aggregates, resulting in damage to dopaminergic neurons via the formation of Lewy bodies—a potential contributor to PD development.

In conclusion, EGF-triggered endocytosis enhances lysosomes, providing protection against zinc-induced neurotoxicity by regulating zinc homeostasis. This intricate interplay among endocytosis activation, lysosomal upregulation, and zinc homeostasis suggests potential strategies for mitigating acute brain injuries by targeting endocytosis pathways and suppressing LMP. Furthermore, these findings emphasize the significance of lysosomal regulation, not only in oxidative stress or zinc-mediated neurotoxicity but also as potential therapeutic targets for managing neurodegenerative diseases like PD, addressing lysosome-related issues in the context of mitochondrial impairment and α-synuclein aggregation.

## Materials and methods

### Cultures of primary mouse cerebrocortical cells and HEK cells

Primary cerebrocortical cells were cultured from ICR mouse embryos collected at embryonic day 13-14 (ORIENT BIO, Gyeonggi, South Korea). In brief, dissociated cortical cells were seeded onto poly-d-lysine (Sigma, St. Louis, MO, USA)-coated plates (SPL Life Sciences, Gyeonggi-do, South Korea), with 4 ~ 5 embryos per plate. These cultures were maintained in a growth medium, consisting of glutamine-free Dulbecco’s modified Eagle’s medium (DMEM; Invitrogen, Carlsbad, CA, USA), supplemented with 2 mM glutamine, 5% fetal bovine serum (FBS; Hyclone, Logan, UT, USA), and 5% horse serum (HS; Invitrogen). The cultures were incubated at 37 °C in a humidified 5% CO_2_ atmosphere. Experiments, except for confocal microscopy and GFP-LAMP-1 overexpression, were conducted using these mixed cerebrocortical cultures, which included both neurons and astrocytes, at 10–12 days in vitro (DIV). For confocal imaging or GFP-LAMP-1 plasmid transfection, near-pure neuronal cultures were used. To prepare near-pure neuronal cultures, primary cerebrocortical cultures were treated with 10 µM cytosine arabinoside (Sigma-Aldrich) at DIV 3 and used at DIV 7. To verify that astrocyte contamination in near-pure neuronal cultures was minimal, immunocytochemistry was performed using microtubule associated protein 2 (MAP2) and glial fibrillary acidic protein (GFAP) antibodies, confirming that astrocytes comprised less than 3% of the cultures. All animal experimental procedures were carried out under the guidelines for Care and Use of Laboratory Animals and were reviewed and approved by the Animal Care and Use Committee of Sejong University.

Human embryonic kidney (HEK) cells were maintained in high glucose DMEM (Welgene, Gyeongsan, South Korea) supplemented with 10% FBS (Invitrogen) and incubated at 37 °C in a humidified 5% CO_2_ atmosphere.

### Chemical treatments

The growth medium, containing 5% FBS and 5% HS, was replaced with a minimal essential medium (MEM; Invitrogen) before chemical exposure. Various compounds, including 100 ng/ml recombinant mouse epithelial growth factor (EGF; R&D, Minneapolis, MN, USA), 1 µM N,N,N’,N’-tetrakis (2-pyridylmethyl)-ethylenediamine (TPEN; Merck, Burlington, MA, USA), 20 µM CA074 methyl ester (CA074, Merck), or 100 µM leupeptin (Thermo Fisher, Waltham, MA, USA), were administered as a 30-min pre-treatment before exposure to 40 µM zinc (Sigma-Aldrich), 140 µM hydrogen peroxide (H_2_O_2_; Thermo Fisher), or 300 µM 1-methyl-4-phenylpyridinium ion (MPP^+^; Abcam, Cambridge, UK). Unless otherwise specified, compounds were pre-treated for 30 min before toxin treatment. HEK cells were subjected to 80 µM zinc, 280 µM H_2_O_2_, or 3 mM MPP^+^ to induce cytotoxicity.

To inhibit the endocytosis of EGF receptor (EGFR), 2 mM methyl-beta-cyclodextrin (MβCD; Sigma-Aldrich), 2 µM chlorpromazine (CP; Sigma-Aldrich), 1 µM compound 56 (Cpd56; Merck), or 500 nM ciliobrevin A (Ciliob; GlpBio, Montclair, CA, USA) were administered as a 30-min pre-treatment before EGF treatment.

### Immunocytochemistry

To confirm the presence of neurons and astrocytes, MAP2 and GFAP antibodies were used, respectively. To verify EGFR expression, an EGFR antibody was utilized and nuclei were counterstained with DAPI. For mixed cortical cultures at DIV10 and near-pure neuronal cultures at DIV7, the cultures were fixed with 4% paraformaldehyde for 15 min, permeabilized with 0.1% Triton X-100 and then blocked with 1% bovine serum albumin in PBS for 30 min. The primary antibodies were incubated for 1 h at room temperature. Antibodies against EGFR (Santa Cruz Biotechnologies), MAP2 (Sigma-Aldrich), and GFAP (Merck) were used. Alexa Fluor 488 or 546-conjugated secondary antibodies were used, and images were captured using a EVOS M7000 microscope (Thermo Fisher, MA, USA).

### LDH release assay

To assess cell death, we measured lactate dehydrogenase (LDH) activity in the culture medium as an indicator of cellular damage, following a previously published protocol [80]. In brief, at the desired time points after zinc exposure, 50 µl of culture medium containing extracellular LDH was mixed with 25 µl of 23 mM pyruvate and 125 µl of 0.03% NADH. The reduction of NADH was kinetically measured for 5 min at 340 nm using a VersaMax absorbance microplate reader (Molecular Devices, San Jose, CA, USA). The LDH value was normalized, with sham-wash set to 0%, and treatment with 100 µM N-methyl-d-aspartic acid (NMDA; Abcam) for cortical neuronal cultures or 500 µM zinc for HEK cells set to 100% in sister cultures.

### Propidium iodide (PI) staining

We also employed propidium iodide (PI) staining to quantify dead cells. After a designated time period following zinc exposure, cells were treated with 2.5 µg/ml PI (Sigma-Aldrich) for 10 minutes at 37 °C, followed by washing with MEM. Stained dead cells were observed using a fluorescence microscope (EVOS Cell Imaging System, Thermo Fisher). Images were randomly selected, and the fluorescence intensity was quantified using the Image J program.

### Cell counting kit-8 (CCK-8) assay

To assess the survival rate of HEK cells, we added CCK-8 solution (Abbkine, Wuhan, China) to 1/20 of the medium’s volume at the designated time point after MPP^+^ exposure. The mixture was then incubated at 37 °C for 3–4 h. The change in color was measured at 450 nm using a VersaMax absorbance microplate reader (Molecular Devices). Cell viability was calculated as 100% for untreated conditions (sham-wash) and 0% using 500 µM zinc as the condition representing complete cell death.

### Microscopic detection of lysosomal activity

To monitor lysosomal activity, neuronal cultures were exposed to 75 nM LysoTracker Red DND-99 (LTR; Invitrogen) or 1 µM Lysosensor Green DND-189 (LSG; Invitrogen) for 30 min at 37 °C. After a wash with MEM, the treated cells were exposed to the respective chemicals for the indicated duration. The intensity of fluorescence directly correlated with the lysosome’s acidity. Fluorescent signals were observed in live cells under a fluorescence microscope, and their intensity was quantified using the Image J program. Fluorescent images were selected randomly, and phase-contrast images from the same area were also provided.

### In situ microscopic detection of cathepsin B activity

To analyze in situ cathepsin B activity, neuronal cultures were exposed to the 1× Magic Red Cathepsin B Detection Kit (Immunochemistry, Minneapolis, MN, USA) following the manufacturer’s protocol. After washing with MEM, the cells were incubated with EGF (100 ng/ml) for the indicated times. Red fluorescence was detected in live cells under a fluorescence microscope, and the fluorescence intensity was measured using the Image J software. The fluorescent images were chosen randomly.

### Confocal imaging for lysosomal zinc

To visualize lysosomal zinc, primary near-pure neuronal cultures plated on poly-d-lysine-coated cover glasses were subjected to double staining with 5 µM Fluozin-3 (Invitrogen) and 75 nM Lysotracker Red for 30 min at 37°C before drug treatment. After removing excess dyes with MEM, cells were exposed to the specified chemical treatments. Subsequently, the cells were fixed in 4% paraformaldehyde for 15 min, followed by twice washing with cold PBS. Post-washing, the cells were immediately mounted and examined using the Leica TCS SP5 confocal laser scanning microscope (Wetzlar, Germany). The fluorescent cells were selected randomly, with a minimum of three fields analyzed for each condition.

### Western blots

Total protein extracts were prepared with RIPA lysis buffer (50 mM Tris, pH 7.5, 150 mM NaCl, 1% NP-40, 0.5% deoxycholic acid, 0.1% sodium dodecyl sulfate [SDS], and 5 mM ethylene-diamine-tetraacetic acid [EDTA] with freshly added 2 µg/ml aprotinin, 2 µg/ml leupeptin, 1 µg/ml pepstatin A, 1 mM phenylmethanesulfonyl fluoride [PMSF], 1 mM sodium orthovanadate [Na_3_VO_4_], 5 mM sodium fluoride, and 10 mM sodium pyrophosphate [Na_4_P_2_O_7_]).

The cytosol fraction was obtained following a previously described method [1]. In brief, after removing the culture medium, cytosol extraction buffer (250 mM glucose, 20 mM hydroxyethyl piperazine ethanesulfonic acid (HEPES), 10 mM KCl, 1.5 mM MgCl_2_, 2 mM EDTA, and 25 µg/ml digitonin) was added to just cover the cell surface. The plate was gently shaken on ice at 100 rpm for 15 min to allow cytosolic components to flow out into the extraction buffer. Acetone was added in a volume four times that of the buffer, and the cytosolic proteins were precipitated overnight at −20 °C. The precipitated protein was collected by centrifugation at 3000 rpm for 20 min at 4 °C, and the pellets were resuspended in cytosol lysis buffer (20 mM Tris, pH 7.4, 150 mM NaCl, 1% Triton X-100, 2 mM EDTA, 2.5 mM Na_4_P_2_O_7_, 1 µM Na_3_VO_4_, 1 µg/ml leupeptin, 1 mM PMSF). Cytosolic proteins were used without quantification.

For electrophoresis, the protein samples were boiled at 95 °C for 5 min, loaded onto SDS-polyacrylamide gels, and transferred to polyvinylidene fluoride (PVDF) membranes. Antibodies against EGFR (Santa Cruz Biotechnologies, Dallas, TX, USA), cathepsin B (Cell signaling Technology, Danvers, MA, USA), and LAMP-1 (Merck) were used. An anti-actin antibody (Abbkine) served as the loading control. Western blot bands were visualized through a chemical reaction involving horseradish peroxidase (HRP) and an enhanced chemiluminescence (ECL; Abbkine) solution, and a gel documentation system (BIS 303 PC, DNF Bio-Imaging Systems, Israel) was employed for documentation. Band densities were analyzed using the Image J program.

### EGFR or lysosomal associated membrane protein-1 (LAMP-1) overexpression

Near-pure cortical neuronal cells were transfected with either the pCMV3-untagged negative control vector (Sino Biological, Beijing, China) or the LAMP-1-GFP plasmid using program CU110 of the Amaxa 4D Nucleofector system (Lonza, Swiss). HEK cells were transfected with 250 ng/ml pCMV3-untagged negative control vector (Sino Biological, Beijing, China), LAMP-1-RFP plasmid or EGFR-GFP plasmid for 4 hours using Lipofectamine 2000 (Invitrogen). The bathing medium was then replaced with DMEM containing 10% FBS and 100 µg/ml gentamicin. The cells were allowed to stabilize for an additional day before the experiment. We gratefully acknowledge Dr. Jung-Jin Hwang (University of Ulsan College of Medicine, Seoul, South Korea) for providing the RFP-LAMP-1 plasmid.

### Statistical analysis

All quantitative data were presented as mean ± standard error of the mean (SEM). Two-group comparisons were performed using a two-tailed Student’s *t*-test. For comparisons involving multiple groups, the entire quantitative dataset was analyzed using an ANOVA test with Dunnett’s multiple comparison post-hoc analysis. The figure legend provides the *p*-value for statistical significance. All data were analyzed using the GraphPad Prism program.

## Supplementary information

Supplementary Figure 1

Supplementary Figure Legends

Western Blot source data

## Data Availability

The authors declare that all data supporting the findings of this study are available within the article and the Supplementary Information. All other data are available from the corresponding authors upon request.
