# Supplementary figures and images for: An increase of lysosomes through EGF-triggered endocytosis attenuated zinc-mediated lysosomal membrane permeabilization and neuronal cell death
Source: Cell Death Dis. 2024 Nov 13;15(11):823. doi: 10.1038/s41419-024-07192-6 (PMC11560978; doi:10.1038/s41419-024-07192-6)

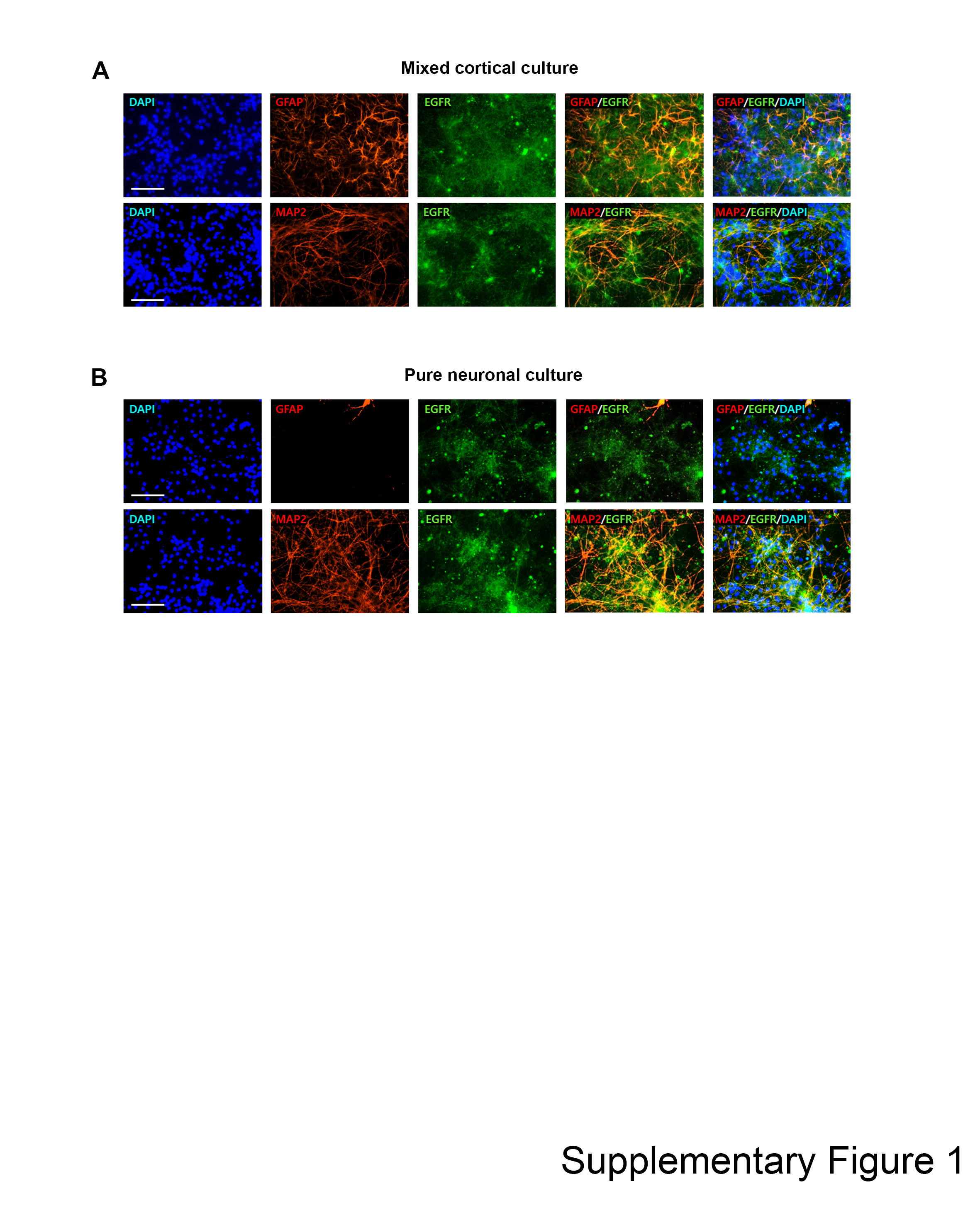

Supplement: Supplementary file 1 — Supplementary Figure 1 [file 41419_2024_7192_MOESM1_ESM.tif]
