# Supplementary material for: An increase of lysosomes through EGF-triggered endocytosis attenuated zinc-mediated lysosomal membrane permeabilization and neuronal cell death
Source: Cell Death Dis. 2024 Nov 13;15(11):823. doi: 10.1038/s41419-024-07192-6 (PMC11560978; doi:10.1038/s41419-024-07192-6)
Supplement: Supplementary file 2 — Supplementary Figure Legends [file 41419_2024_7192_MOESM2_ESM.doc]

**SUPPLEMENTARY FIGURE LEGENDS**

**Supplementary Data 1.** Expression of EGFR in neurons and astrocytes in primary mouse cerebrocortical cultures. **A.** Microscopic images of neurons and astrocytes stained with MAP2 or GFAP, and EGFR in mixed cortical cultures at DIV 10. DAPI was used for nuclei counterstaining. Scale bar: 150 µm. **B.** Microscopic images of neurons and astrocytes stained with MAP2 or GFAP, and EGFR in near-pure cortical neuronal cultures at DIV 7. Scale bar: 150 µm. GFAP positive astrocytes were observed in less than 3% of the cultures. EGFR is widely expressed in neurons.
