# Supplementary material for: An increase of lysosomes through EGF-triggered endocytosis attenuated zinc-mediated lysosomal membrane permeabilization and neuronal cell death
Source: Cell Death Dis. 2024 Nov 13;15(11):823. doi: 10.1038/s41419-024-07192-6 (PMC11560978; doi:10.1038/s41419-024-07192-6)

Figure 1B-EGFR_#1

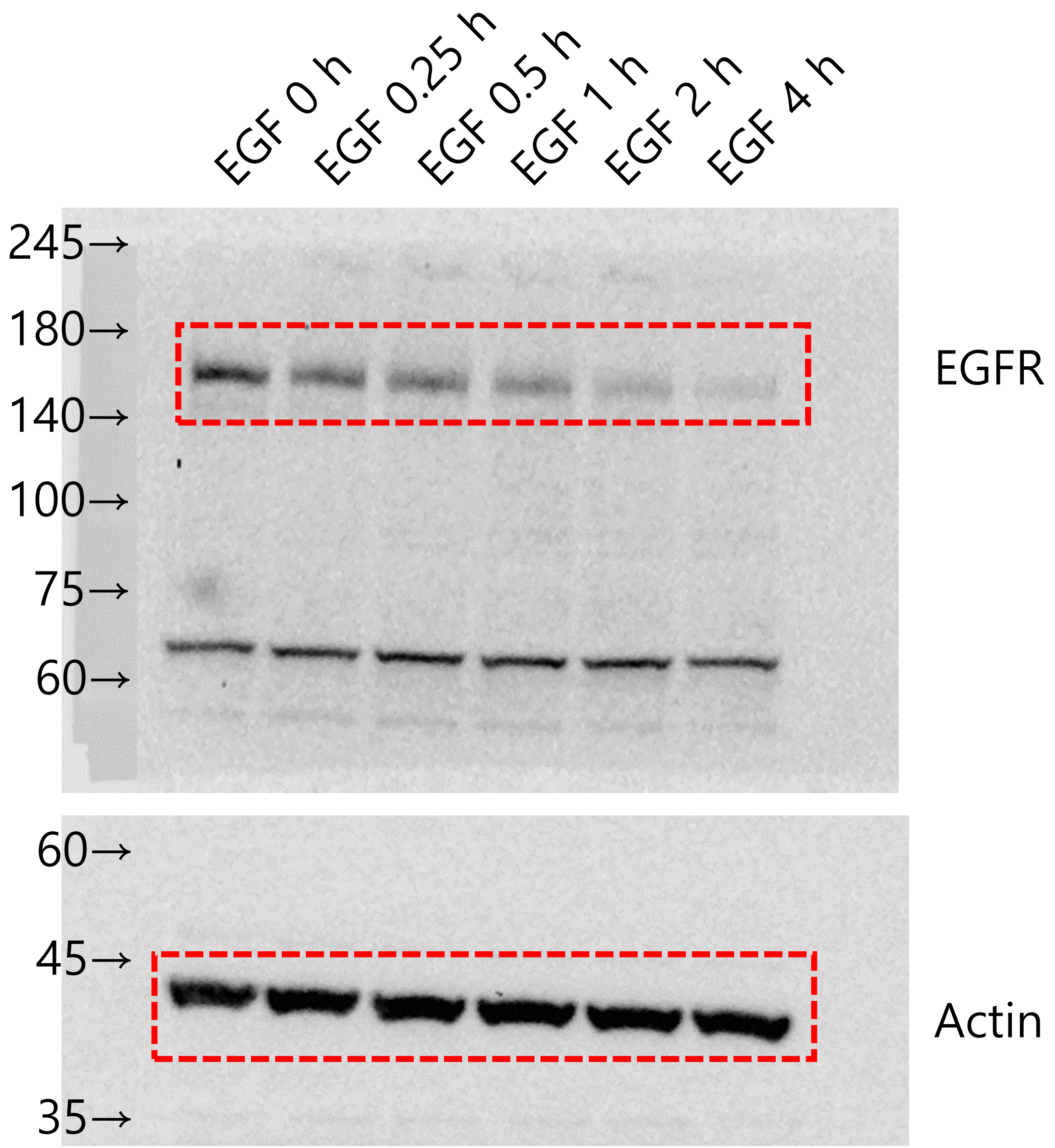

Figure 1B-EGFR_#2

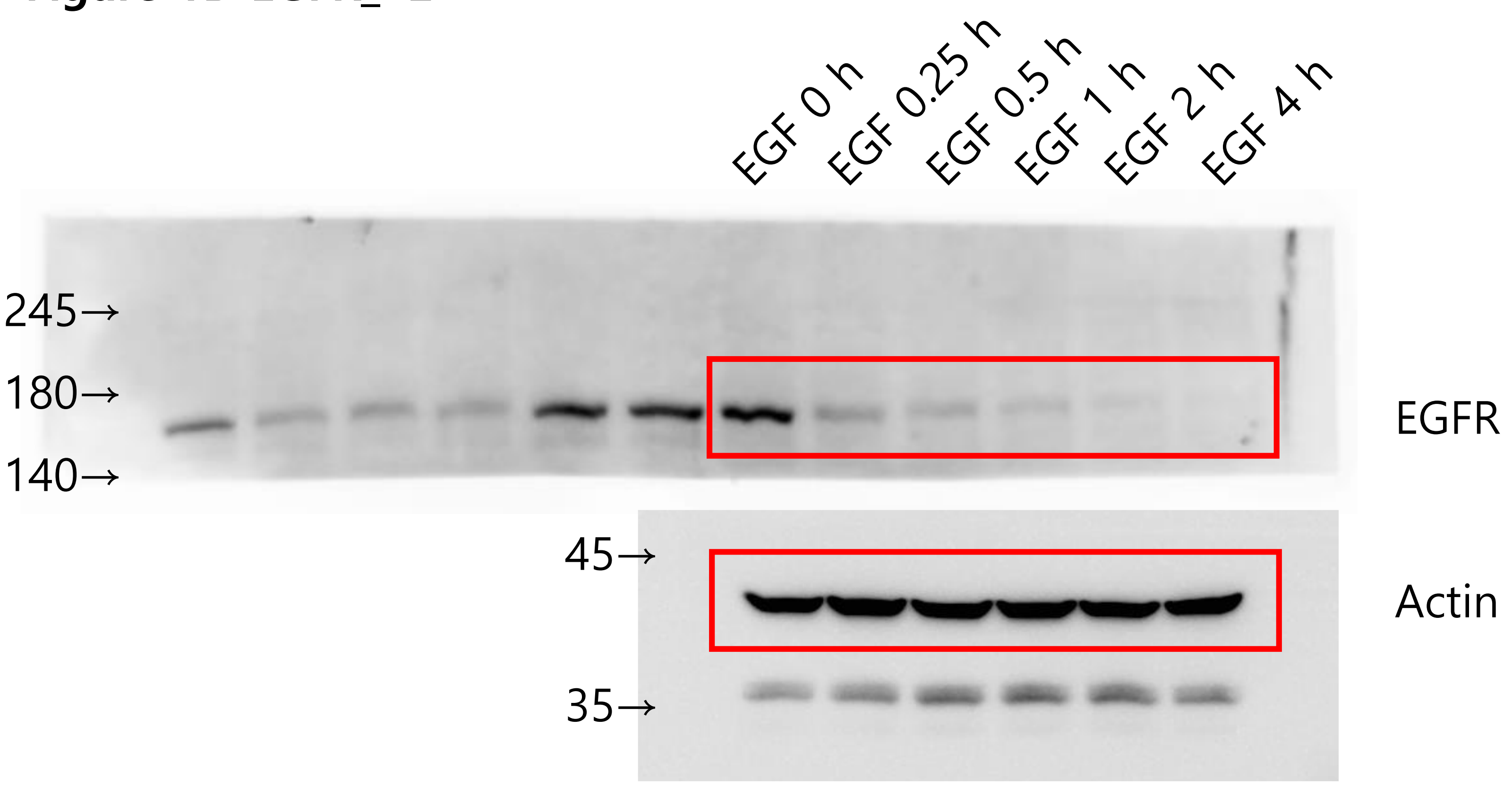

Figure 1B-EGFR_#3

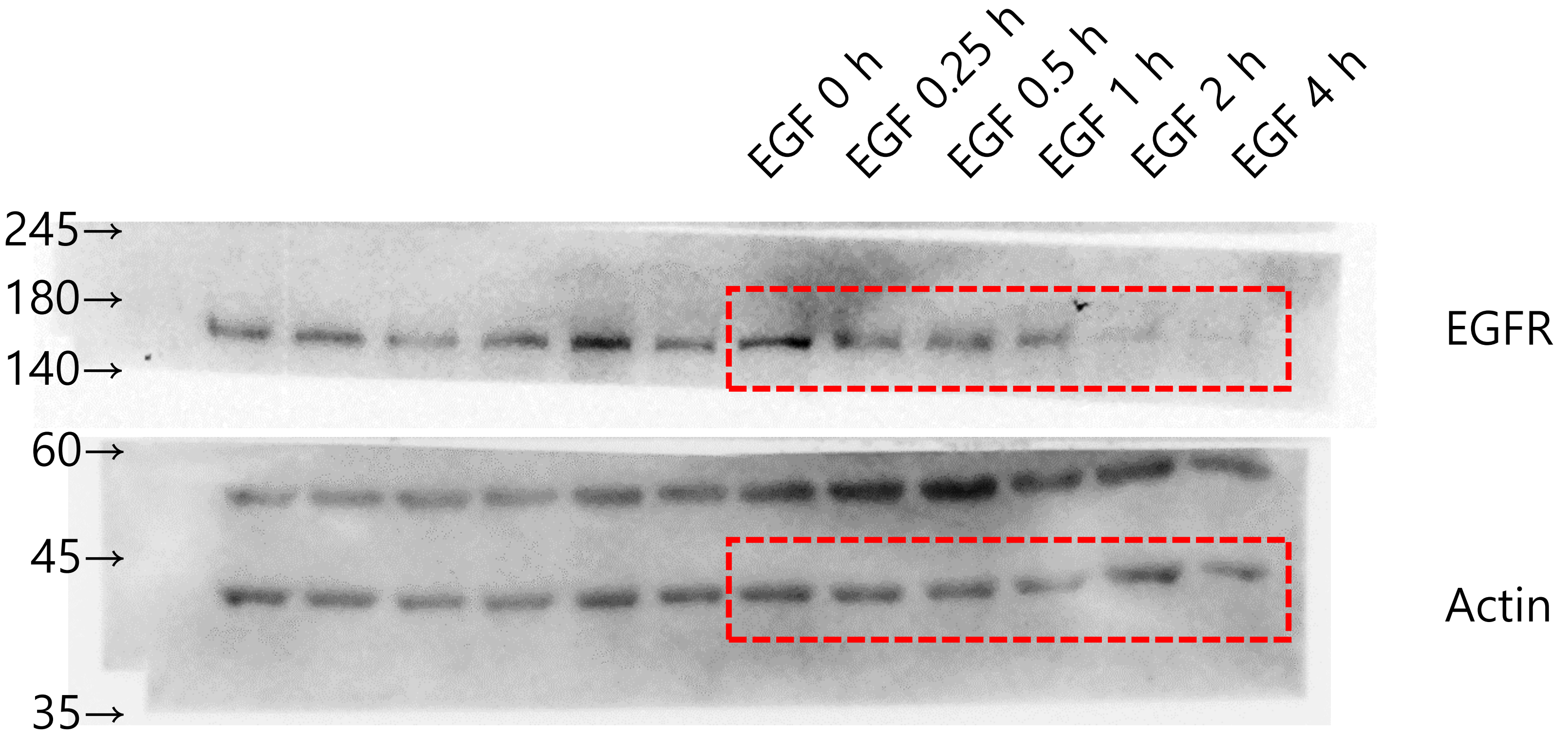

Figure 1B-LAMP-1_#1

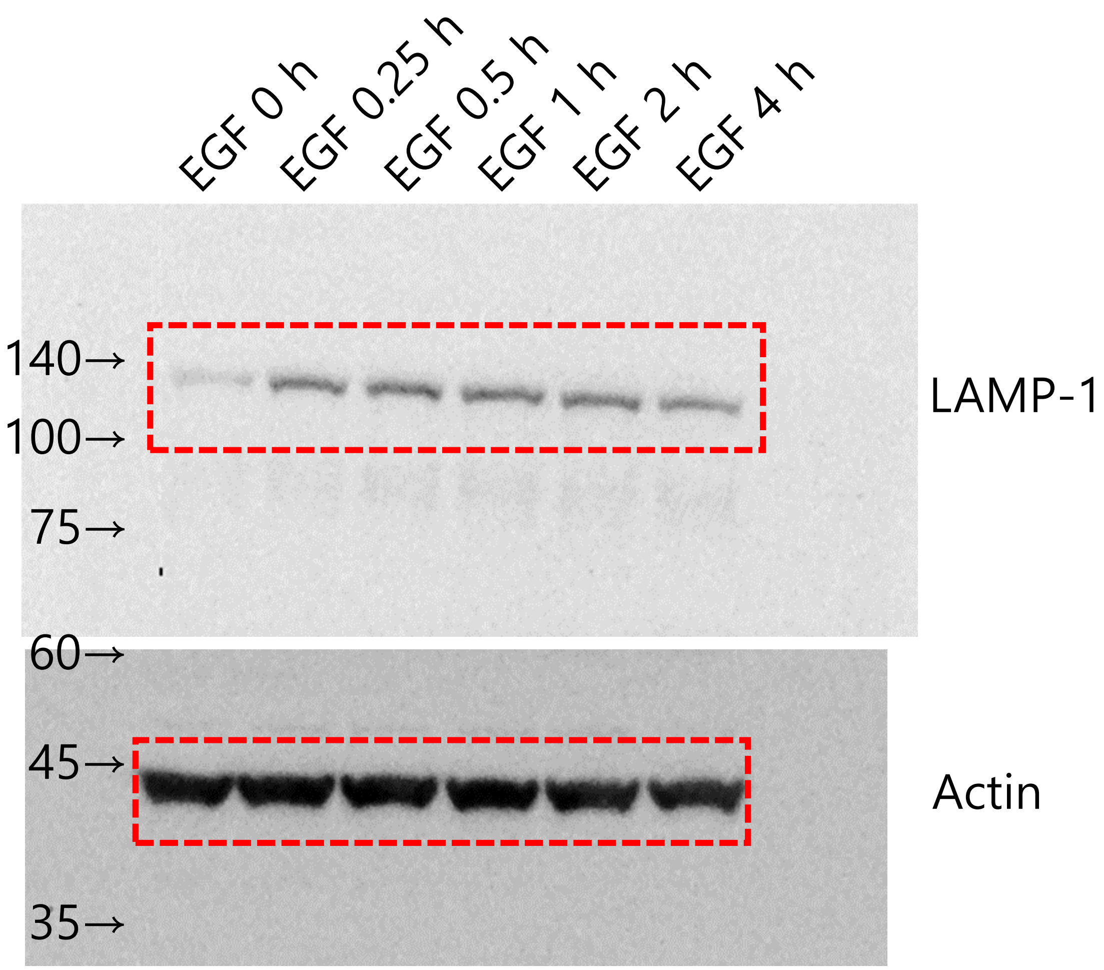

Figure 1B-LAMP-1_#2

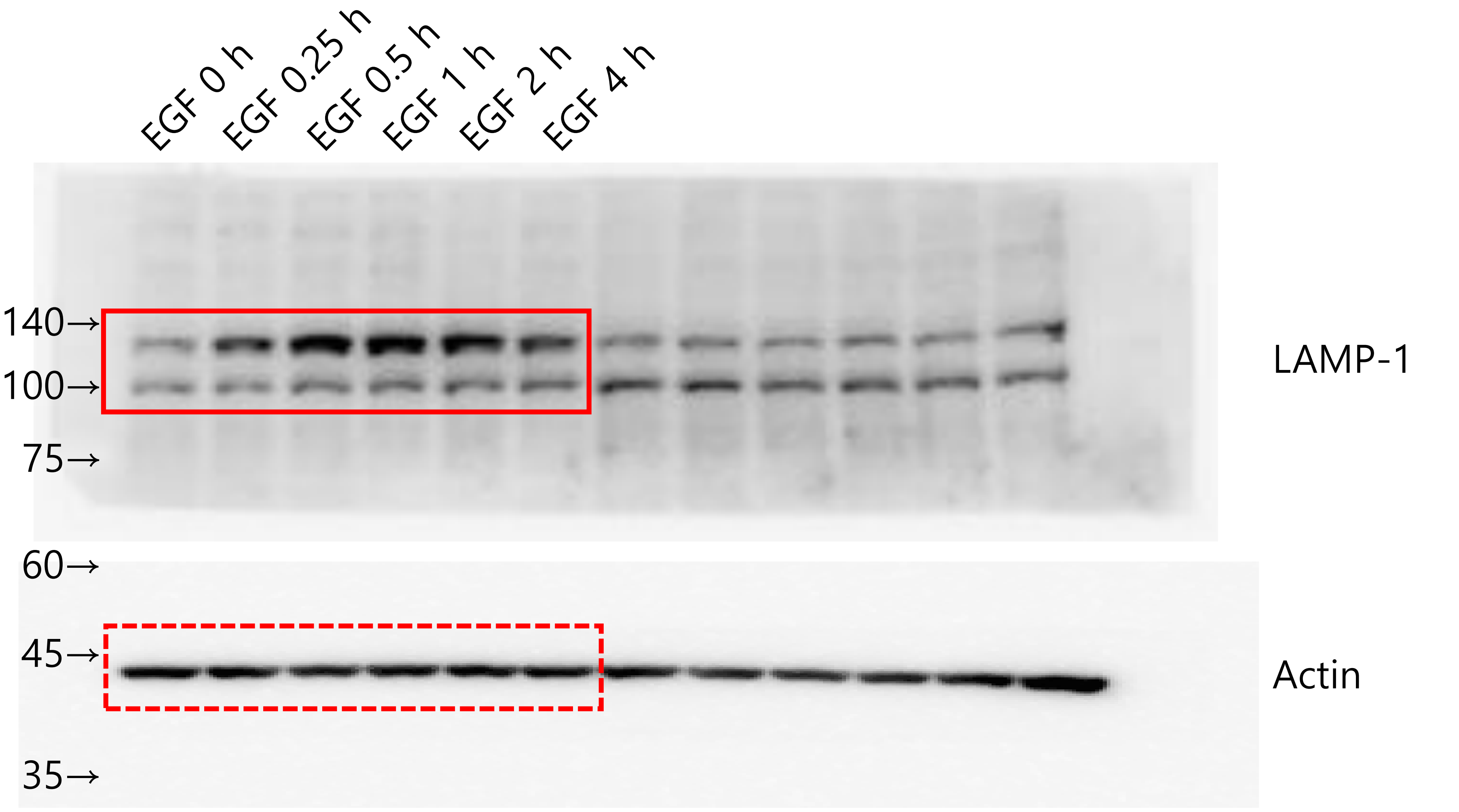

Figure 1B-LAMP-1_#3

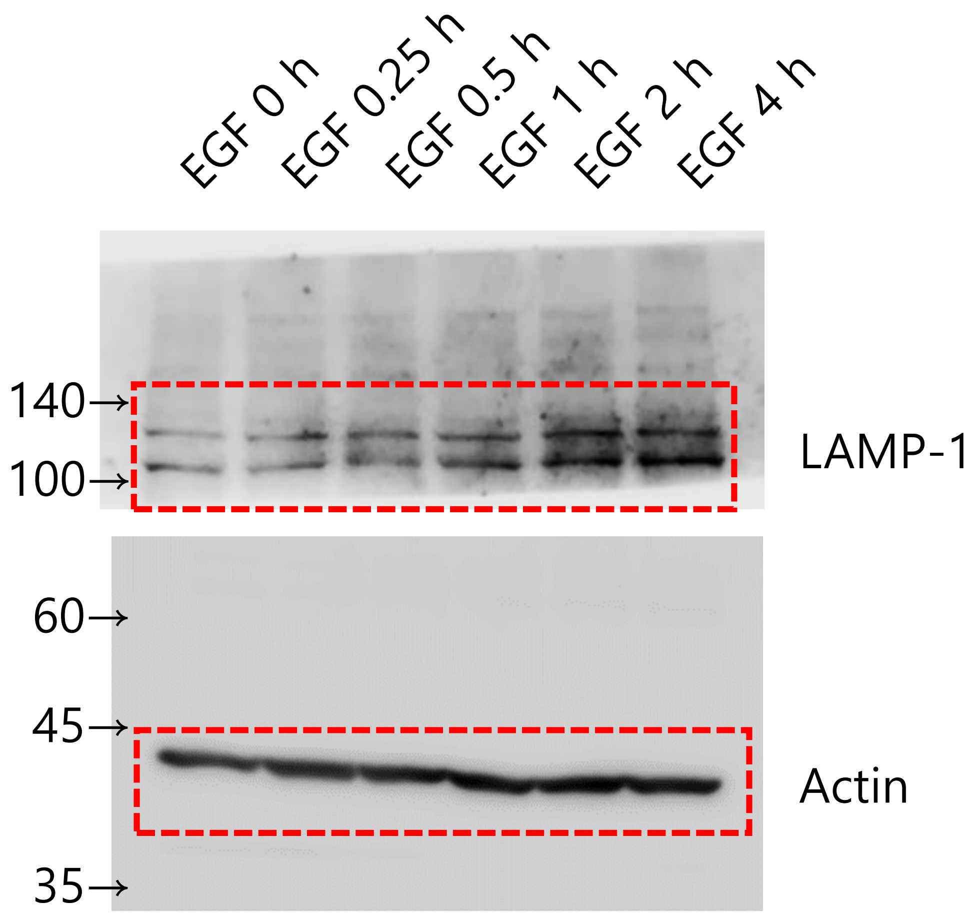

Figure 1C-CTSB(pro, mature)_#1

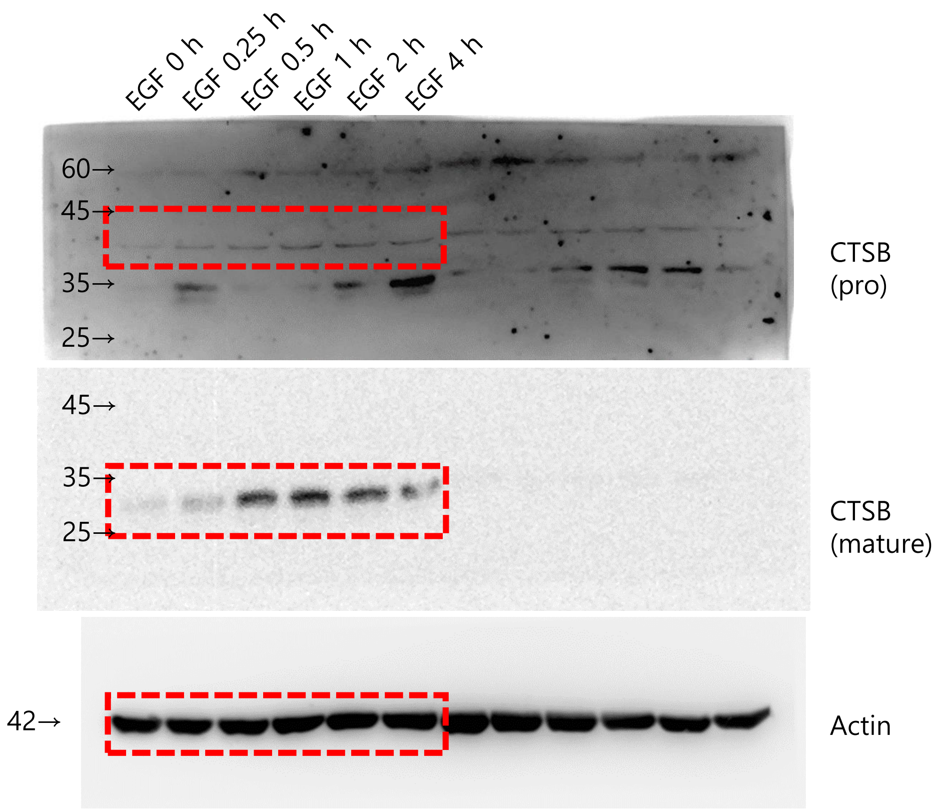

Figure 1C-CTSB(pro, mature)_#2~6

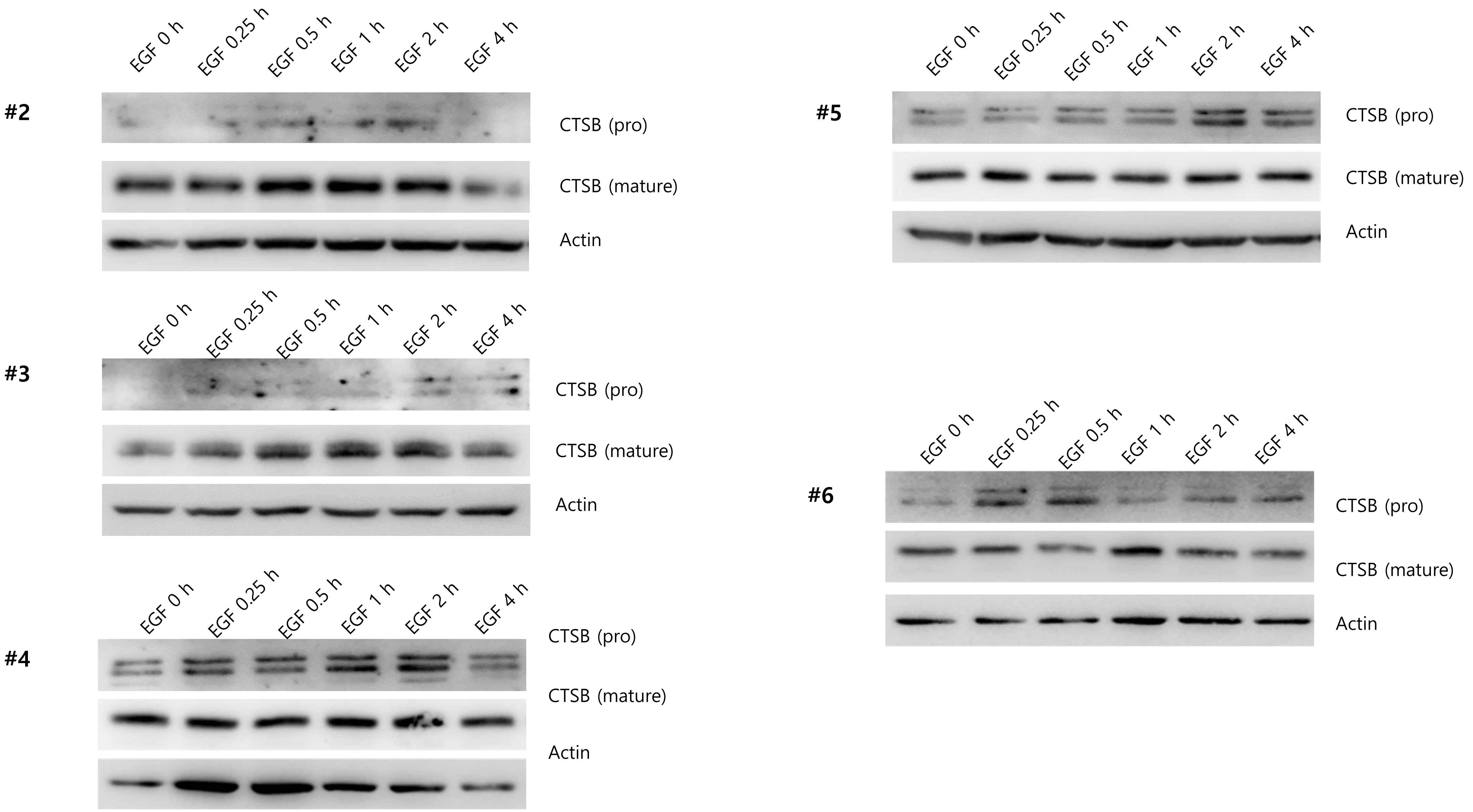

Figure 3B-Mature cathepsin B_#1

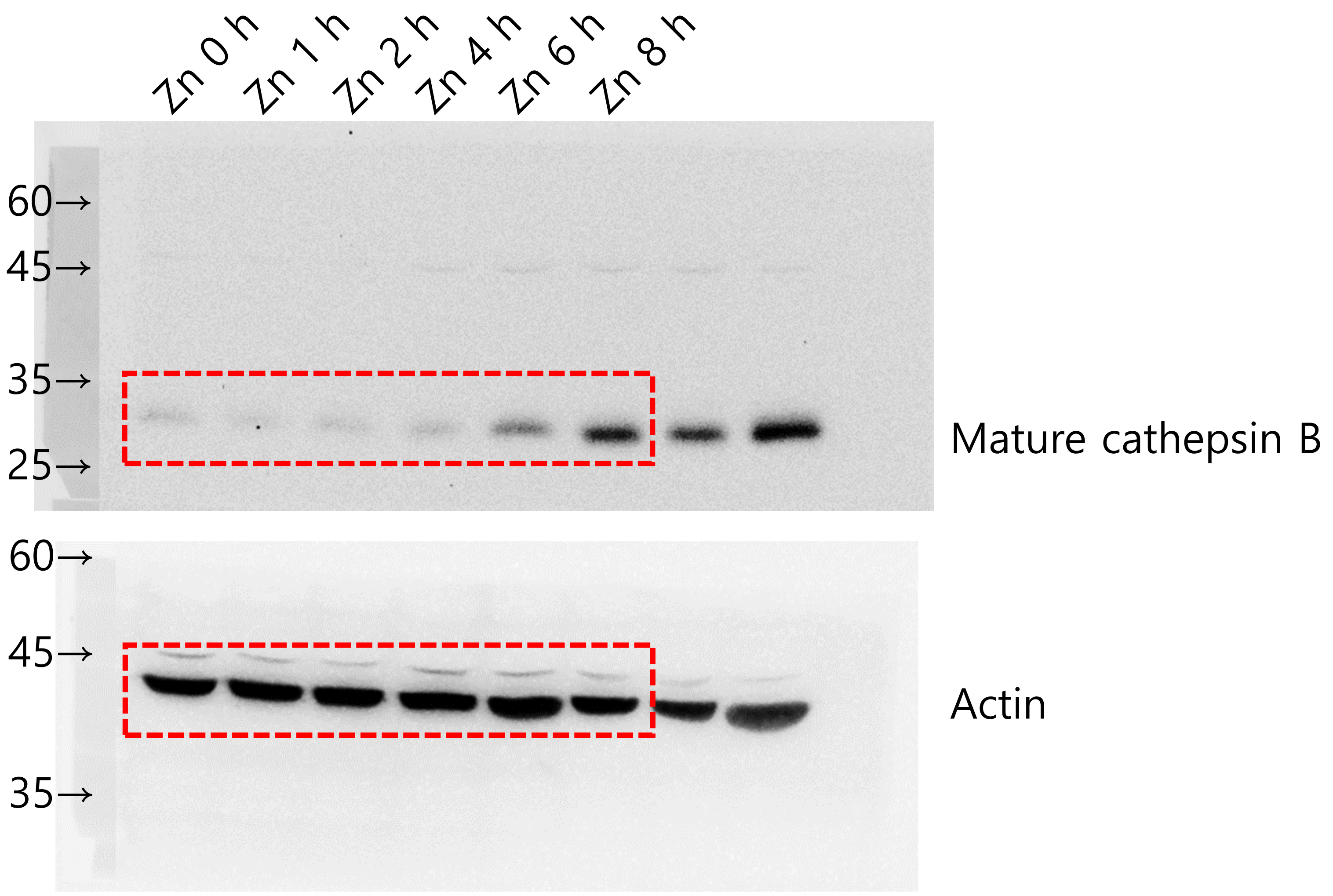

Figure 3B-Mature cathepsin B_#2

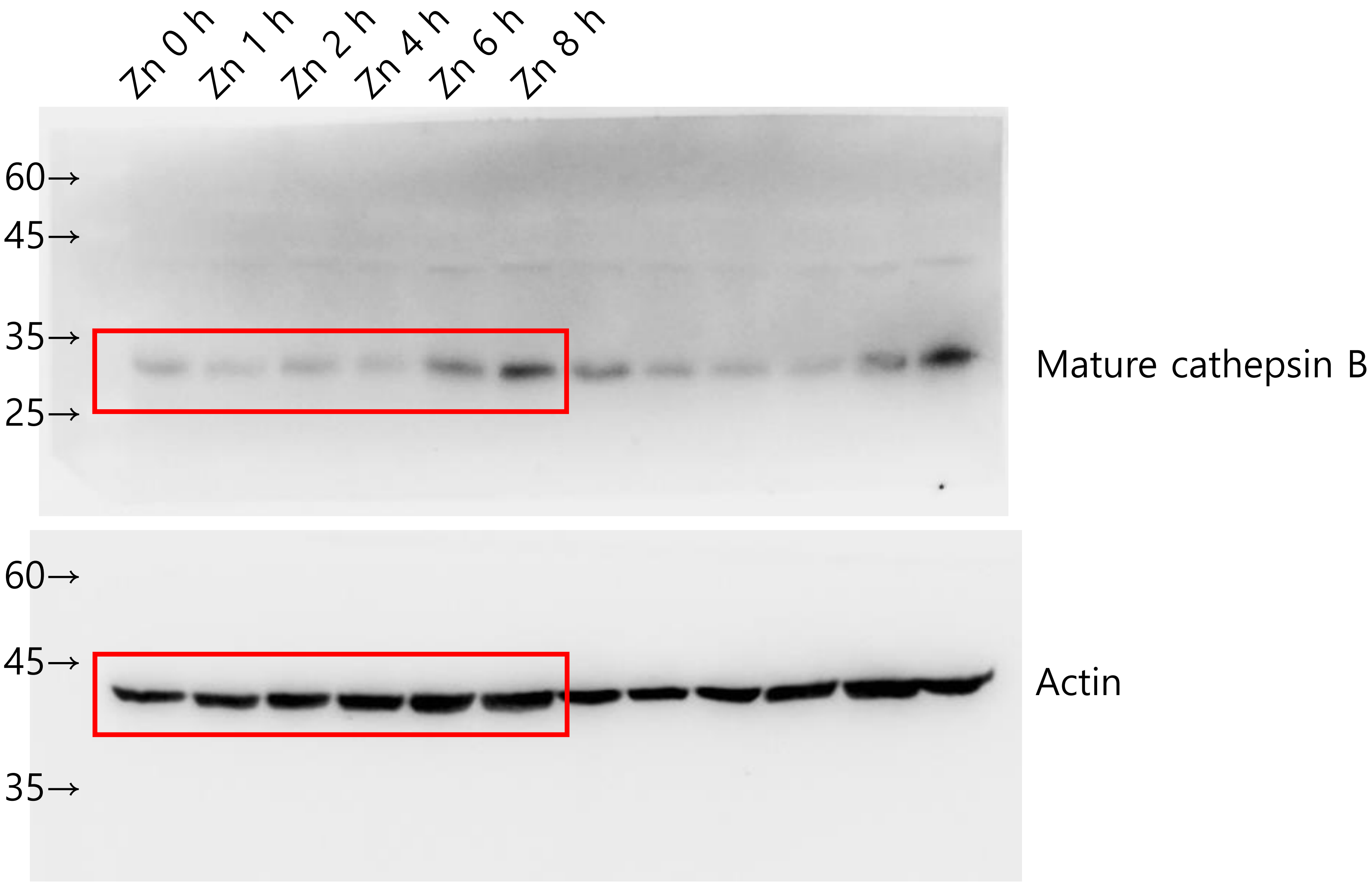

Figure 3B-Mature cathepsin B_#3

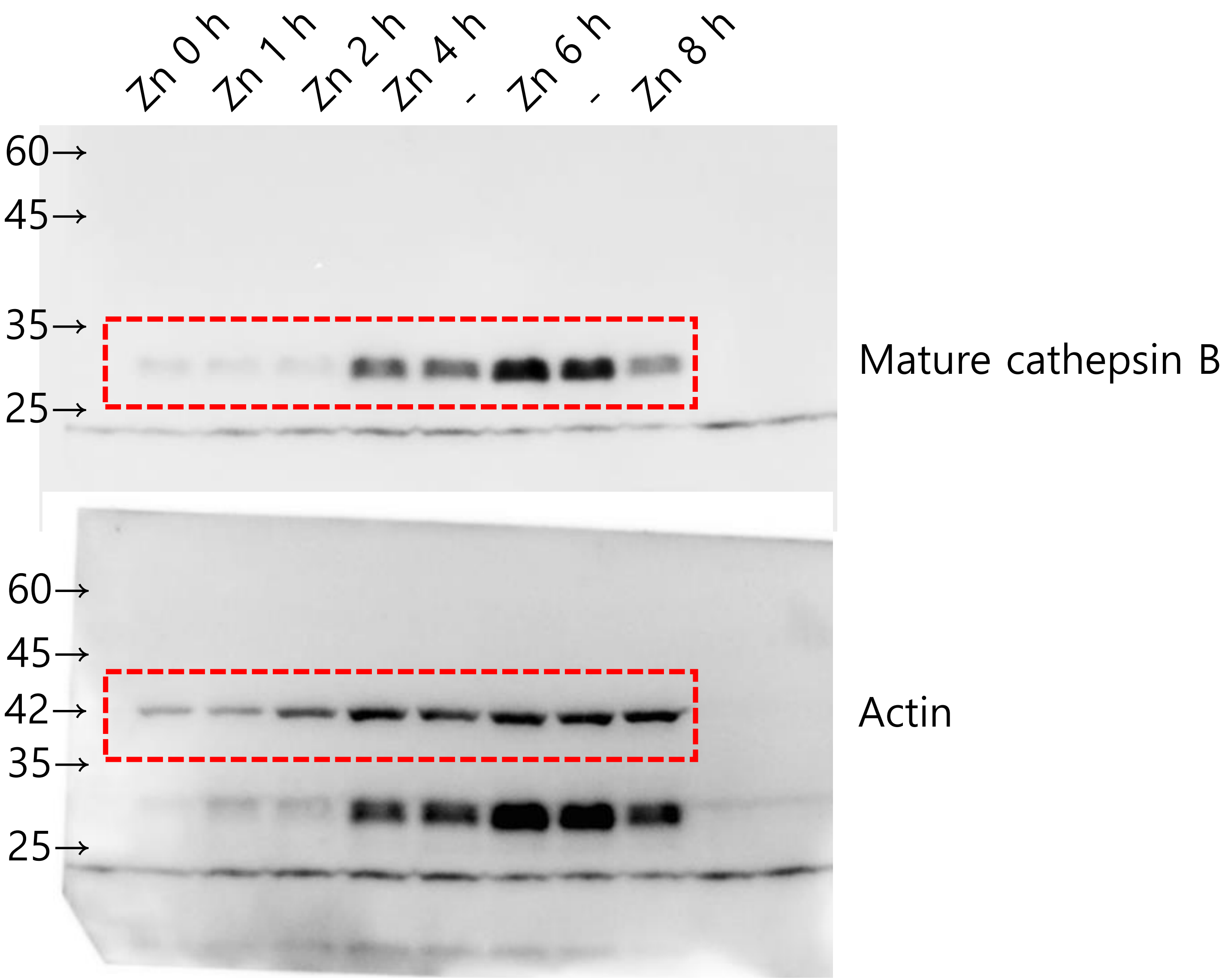

Figure 3B-Mature cathepsin B_#4

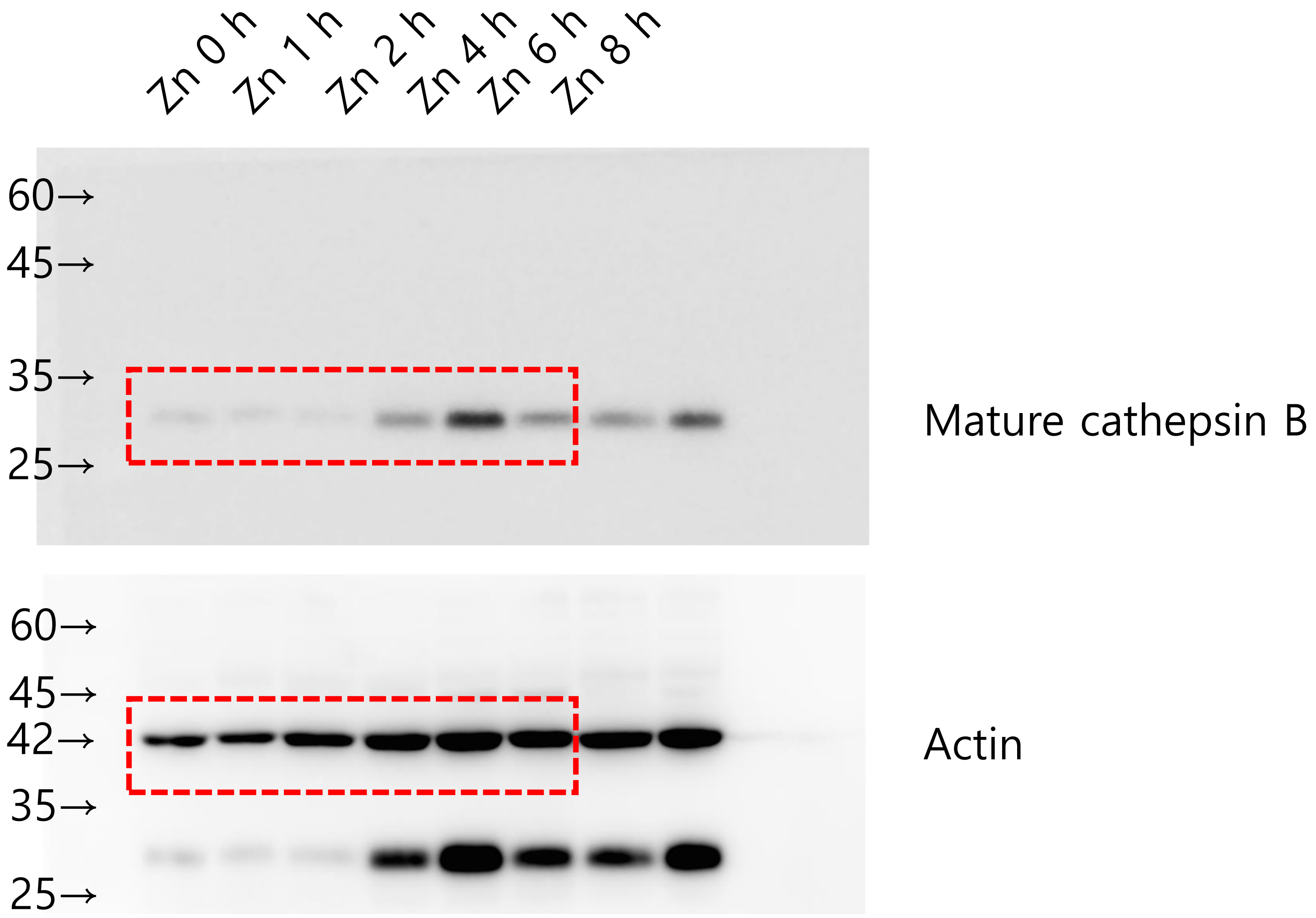

Figure 3D-Mature cathepsin B_EGF_#1

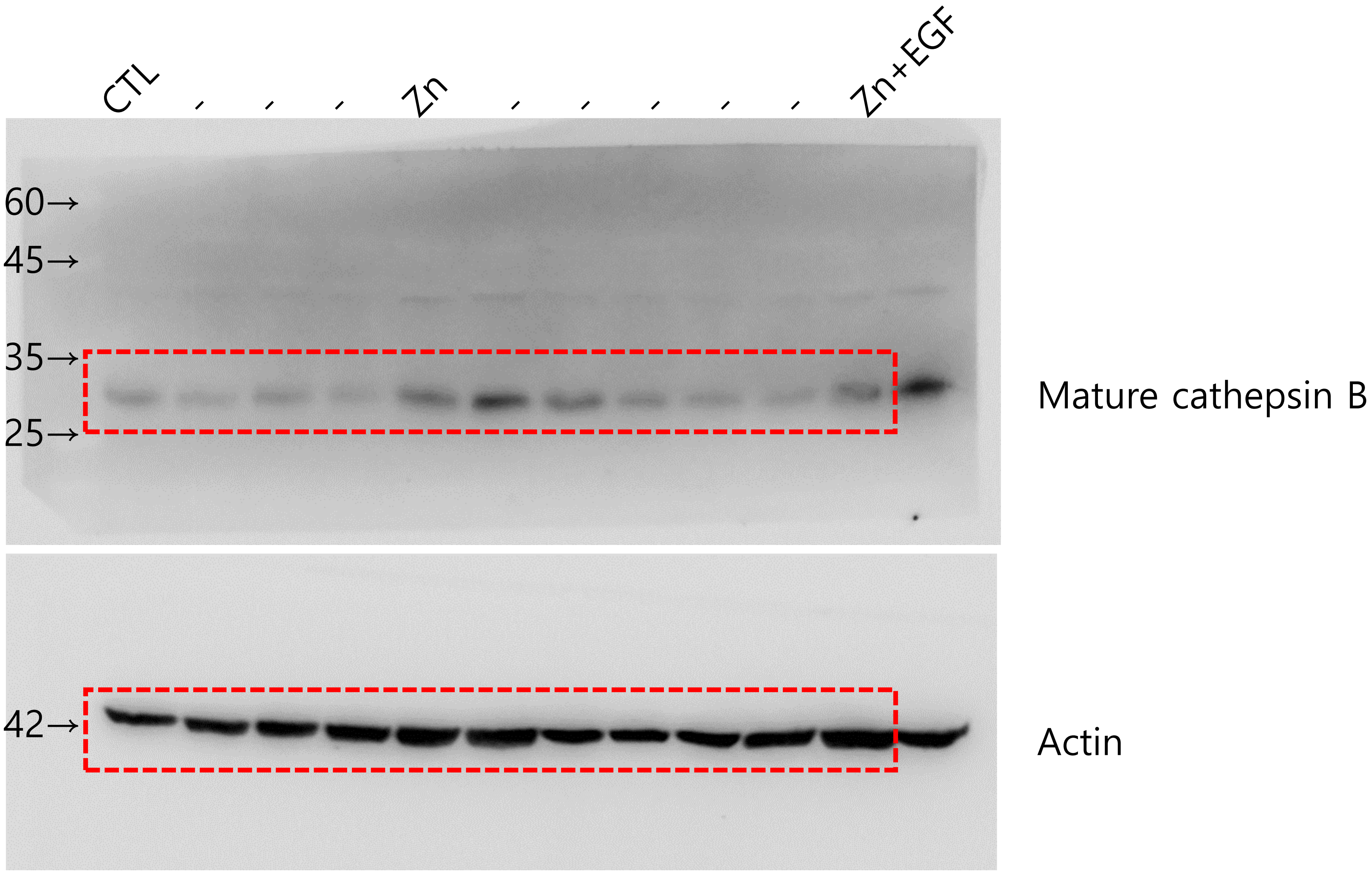

Figure 3D-Mature cathepsin B_EGF_#2

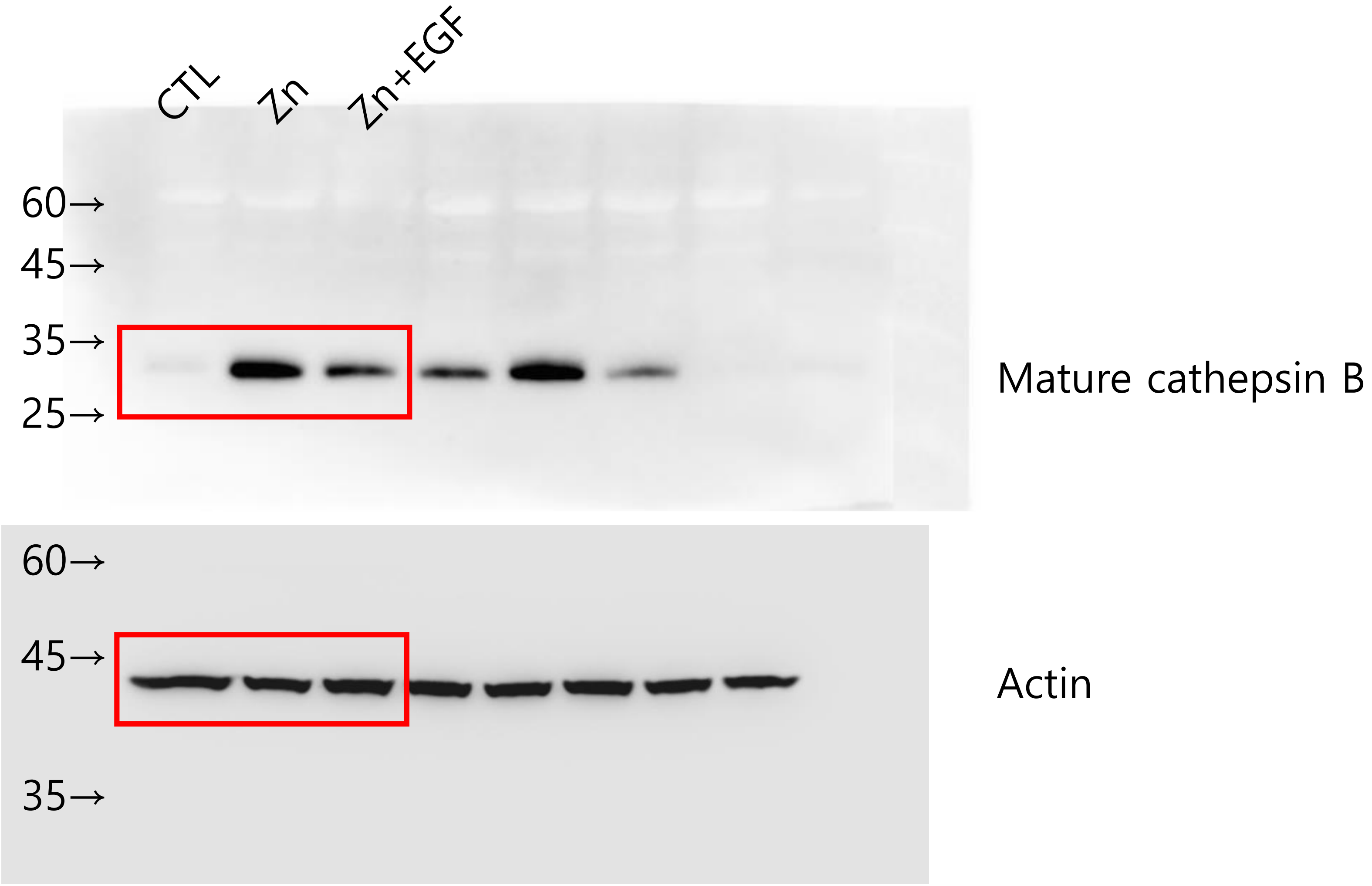

Figure 3D-Mature cathepsin B_EGF_#3

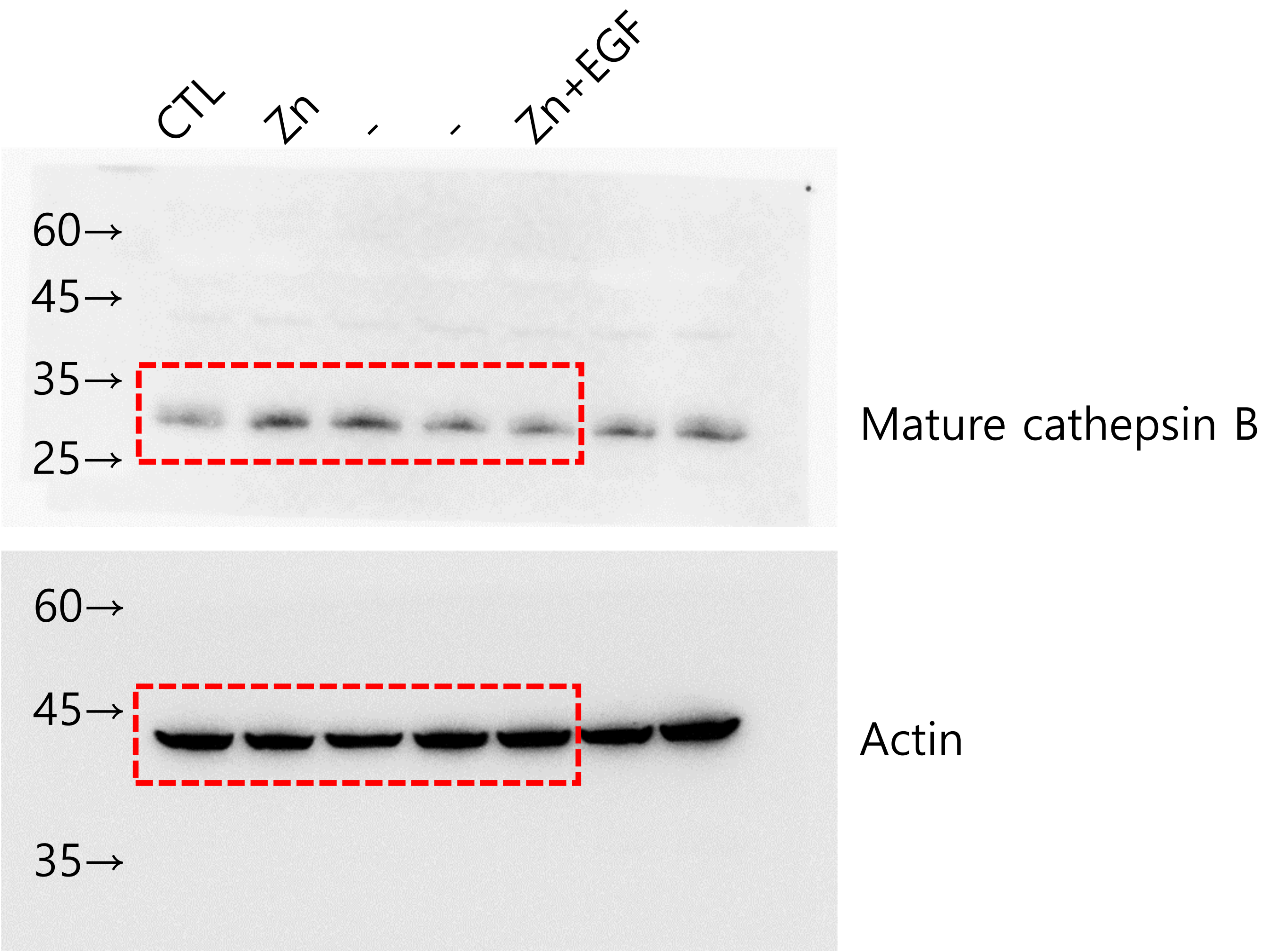

Figure 3D-Mature cathepsin B_EGF_#4

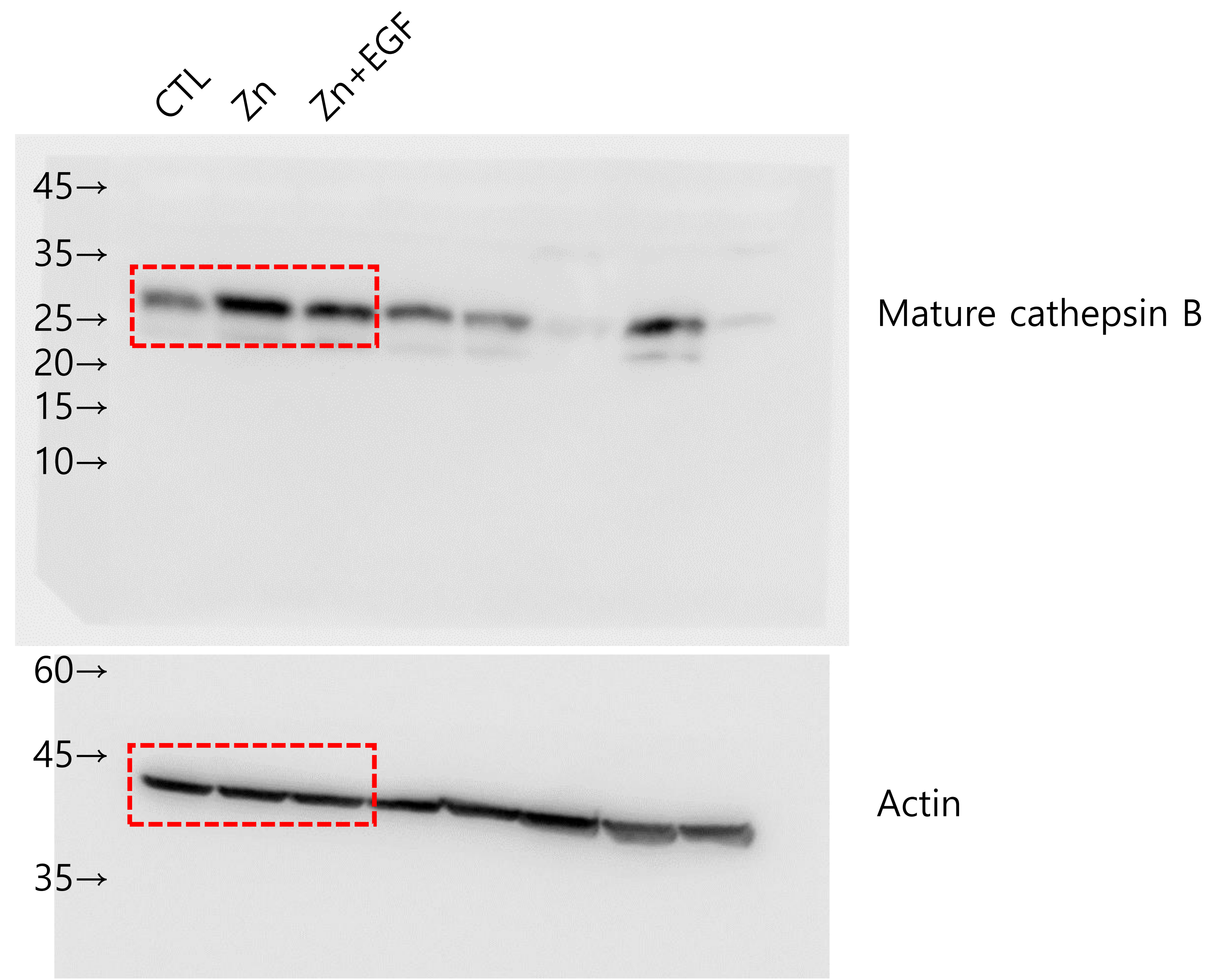

Figure 3D-Mature cathepsin B_EGF_#5

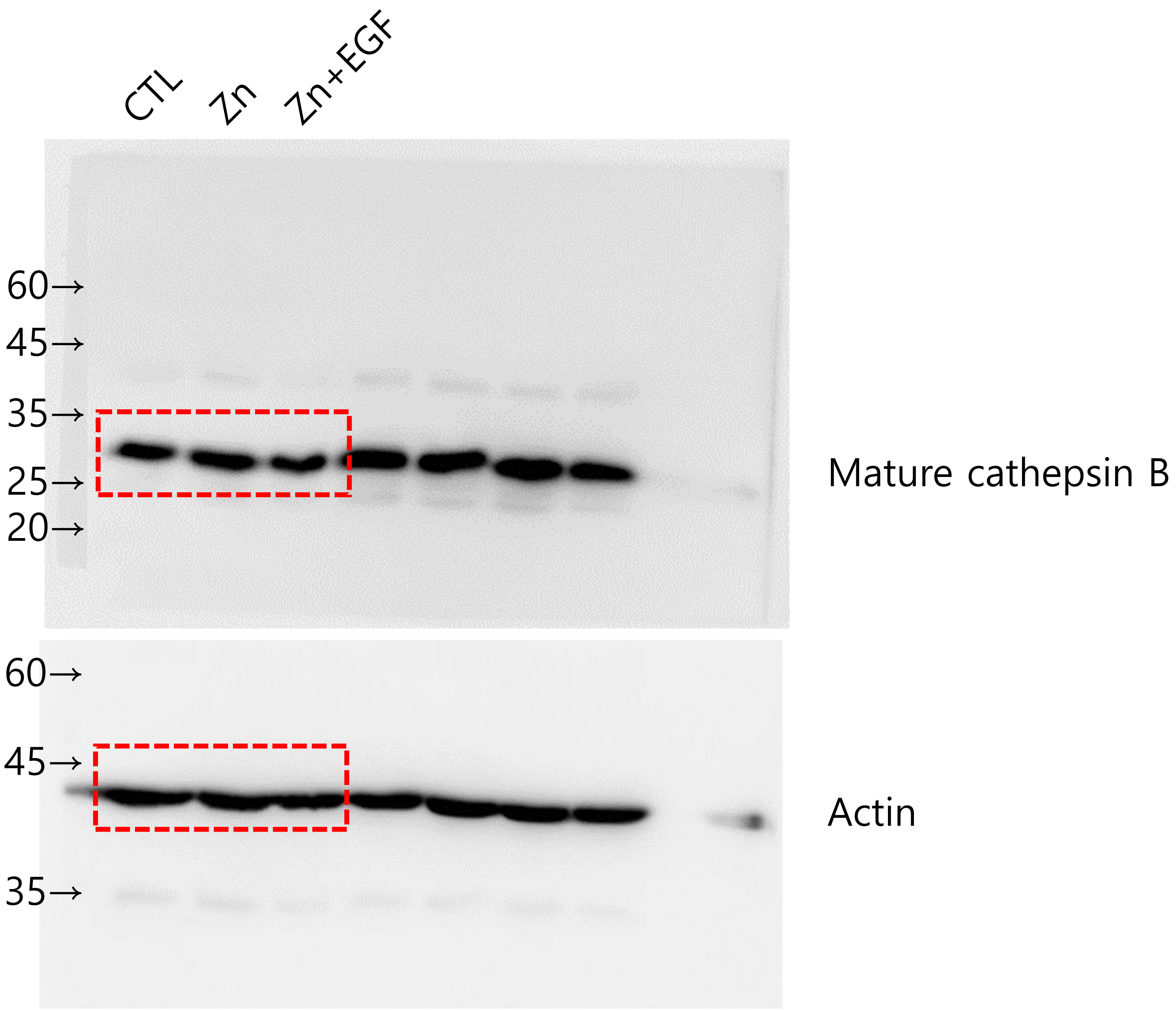

Figure 3D-Mature cathepsin B_TPEN_#1

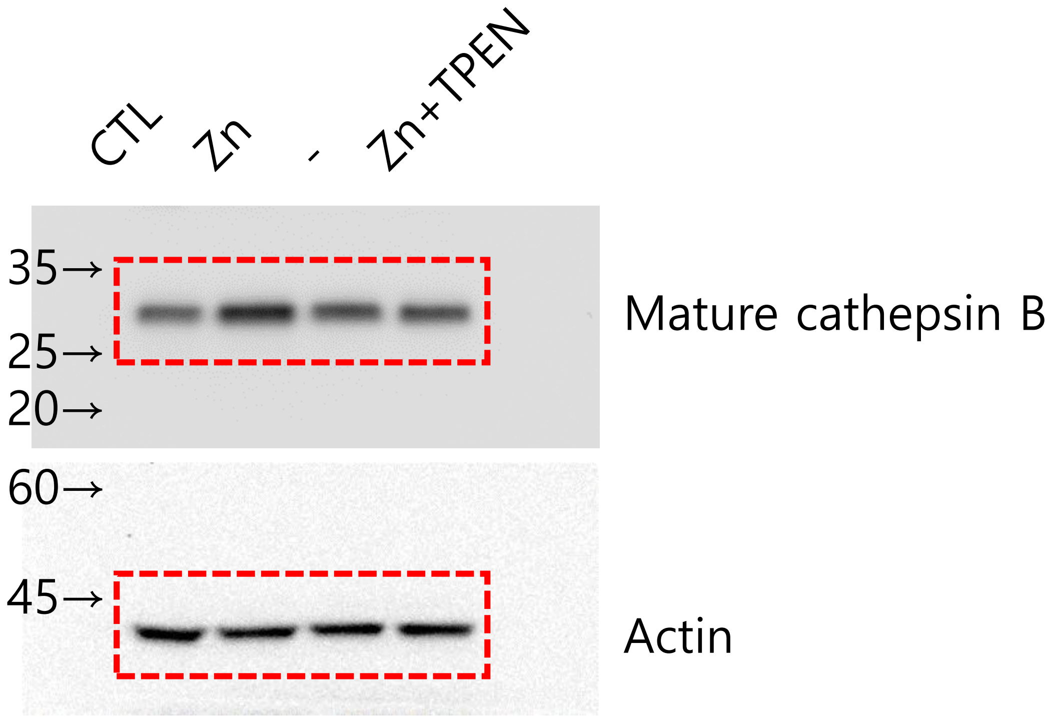

Figure 3D-Mature cathepsin B_TPEN_#2

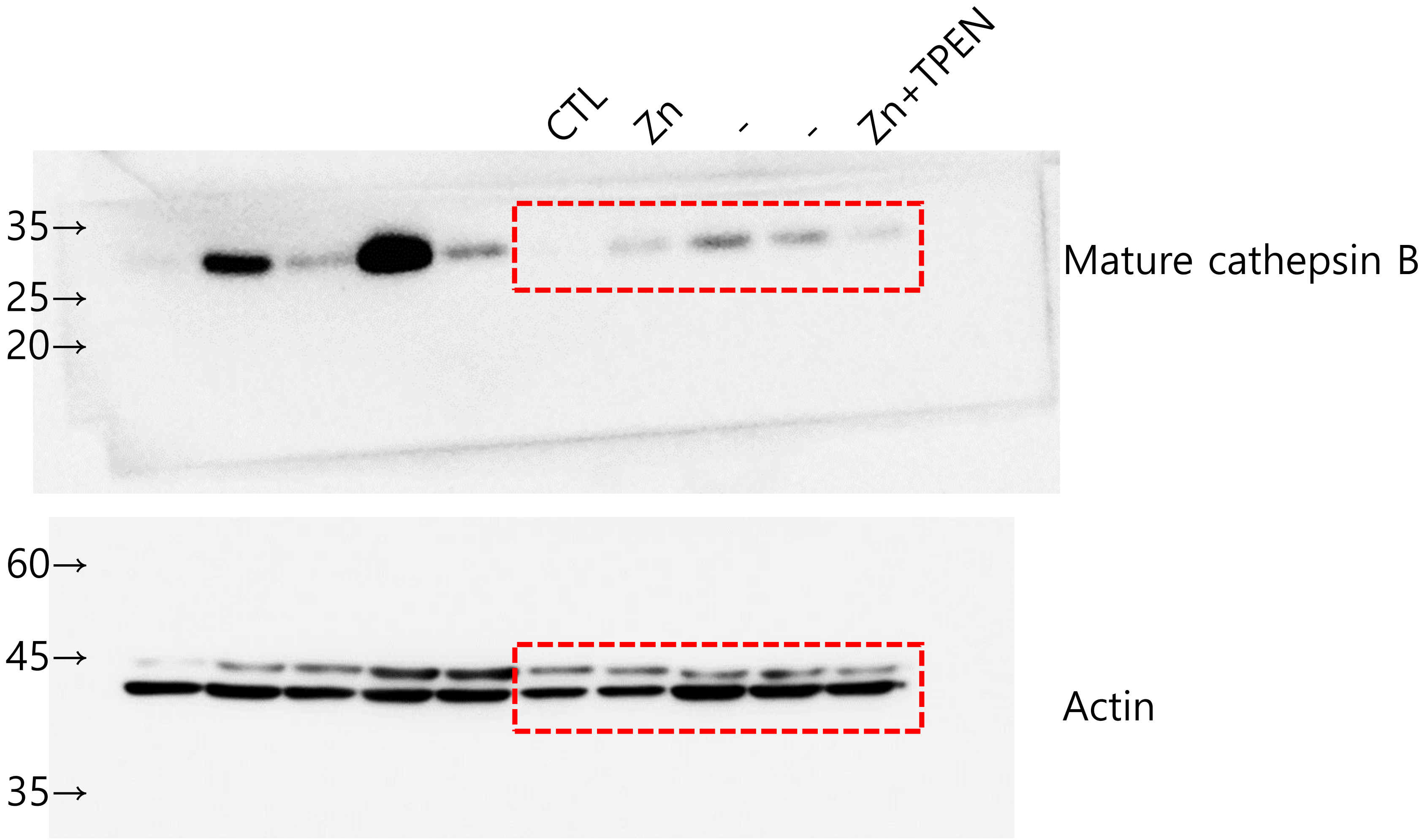

Figure 3D-Mature cathepsin B_TPEN_#3

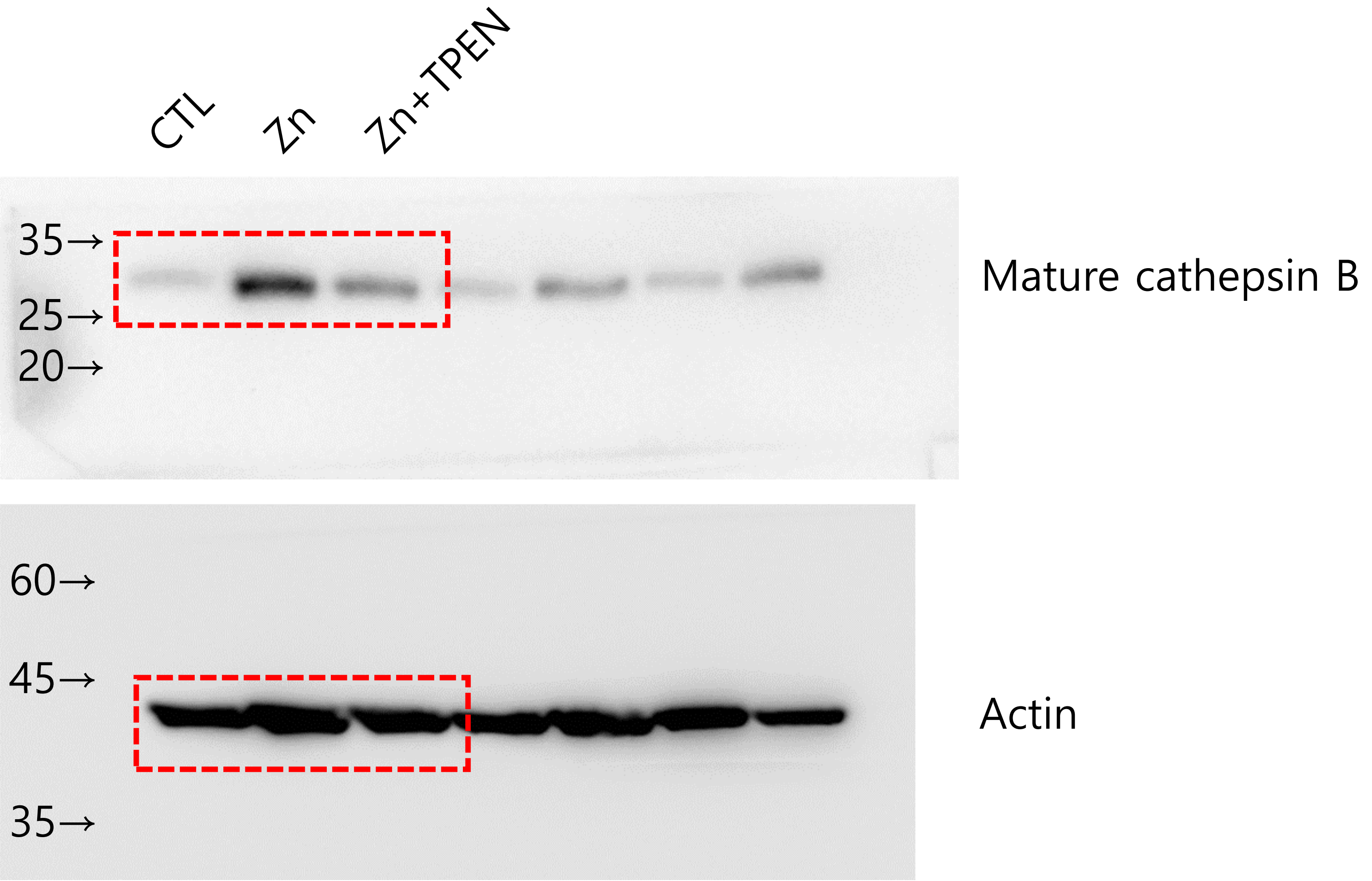

Figure 3D-Mature cathepsin B_TPEN_#4

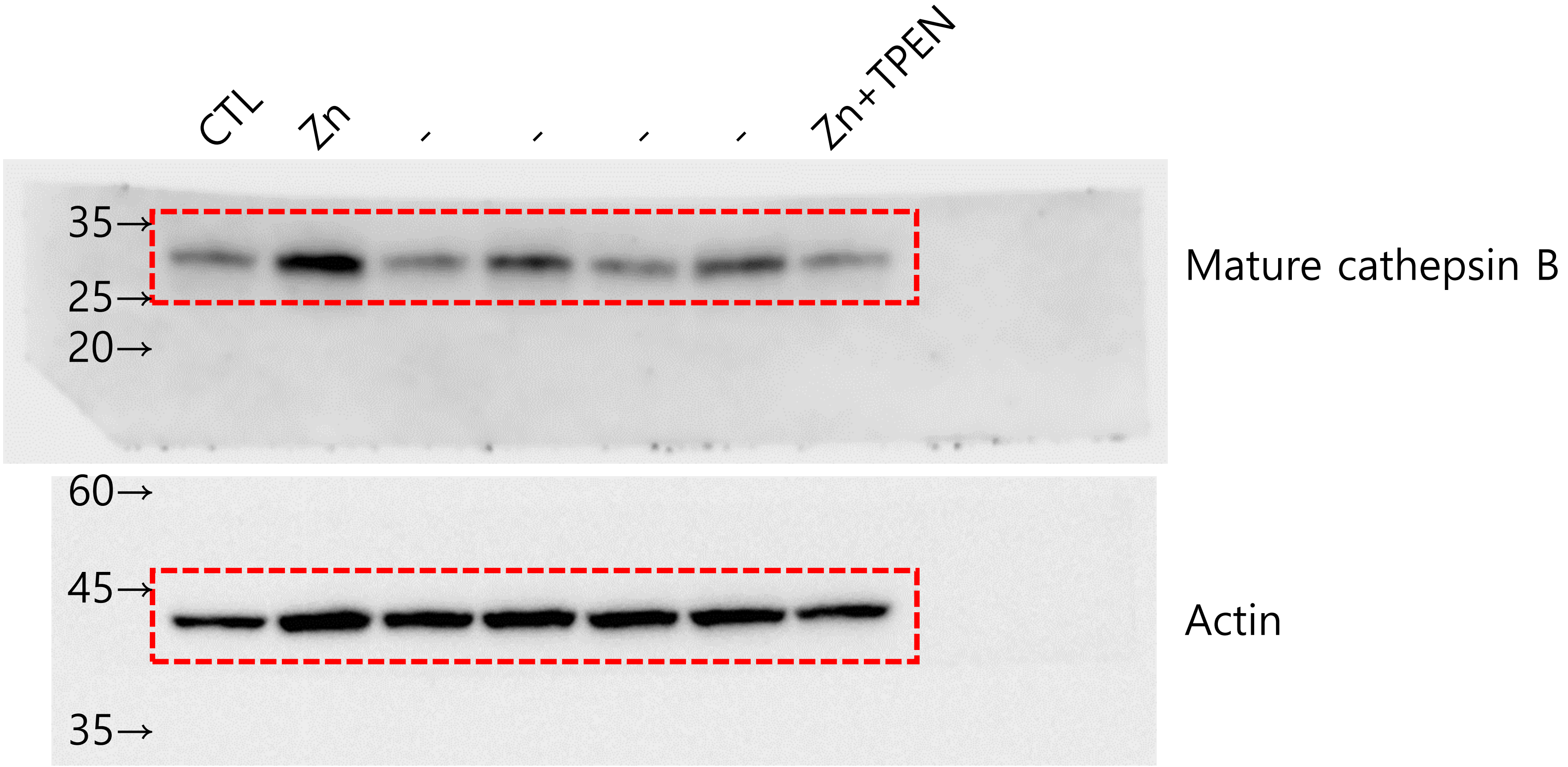

Figure 3D-Mature cathepsin B_TPEN_#5

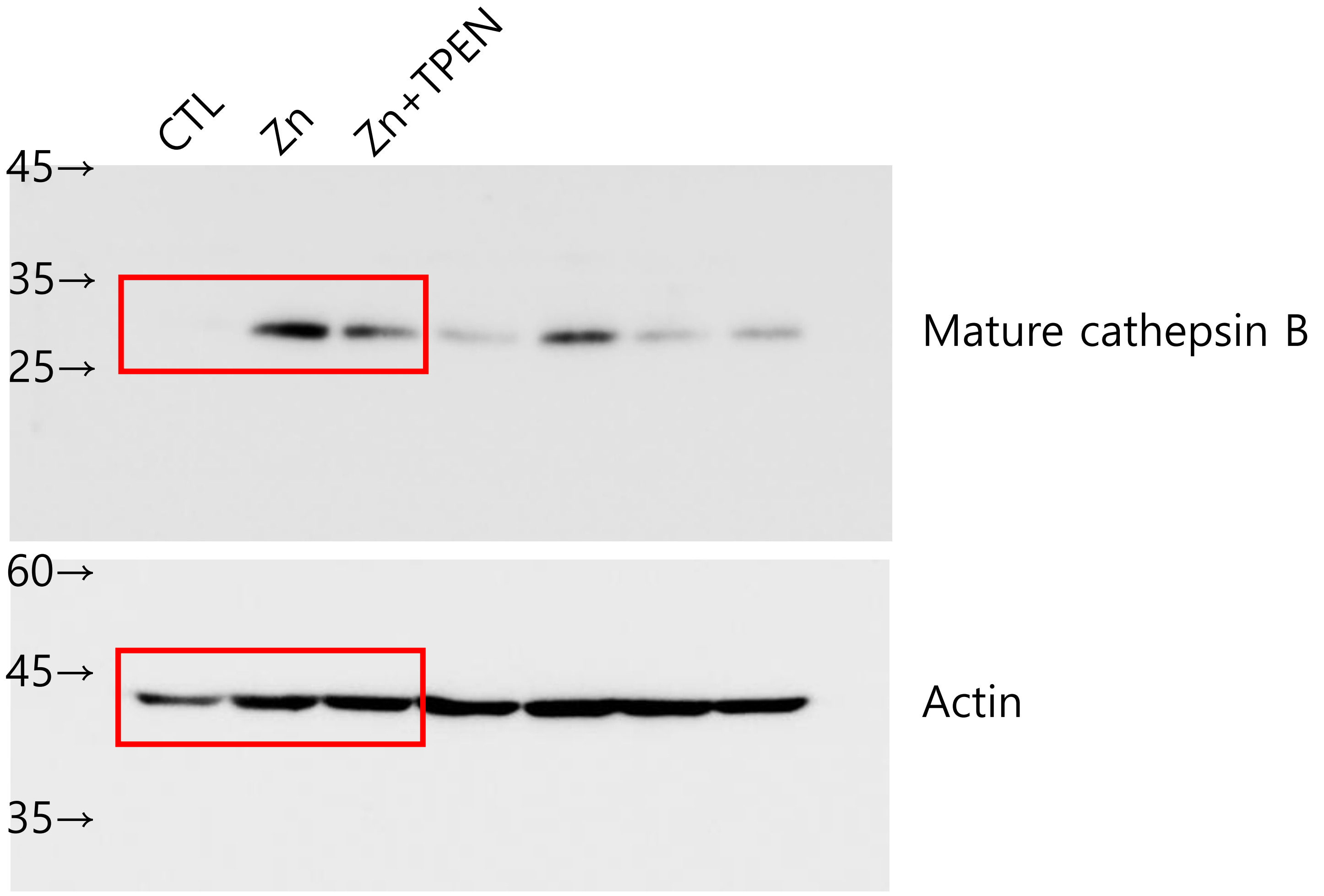

Figure 3D-Mature cathepsin B_leupeptin/CA074_#1

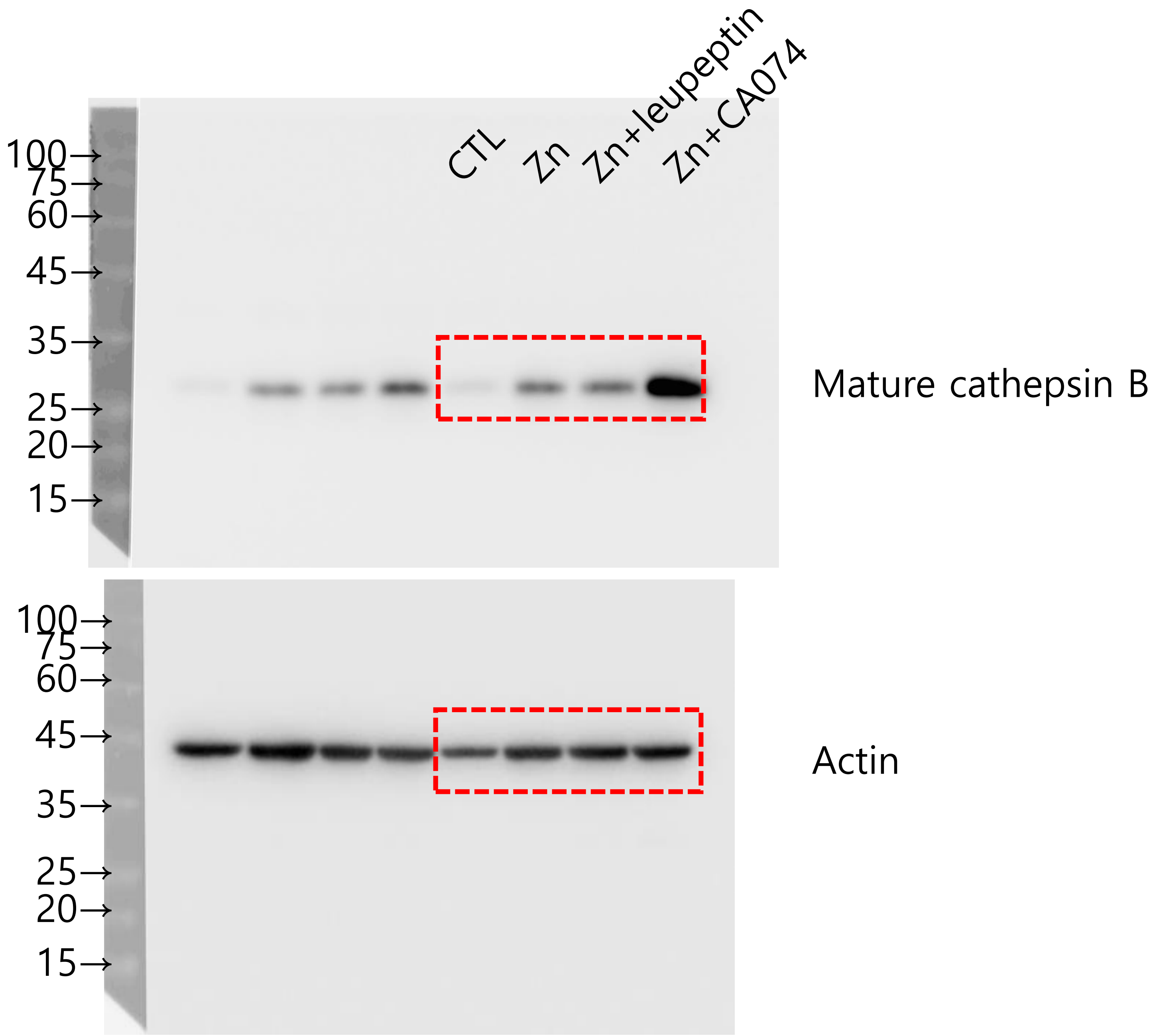

Figure 3D-Mature cathepsin B_leupeptin/CA074_#2

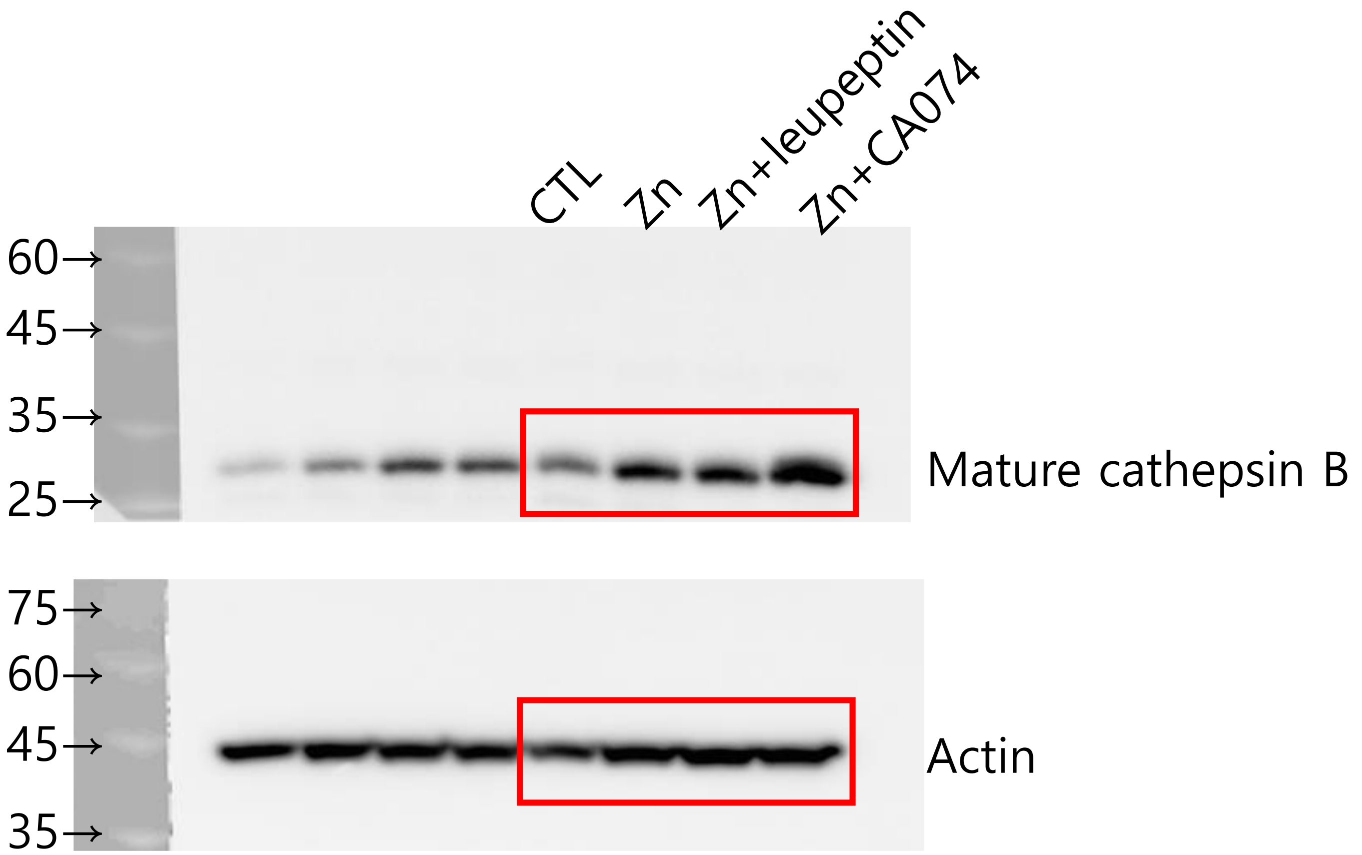

Figure 3D-Mature cathepsin B_leupeptin/CA074_#3

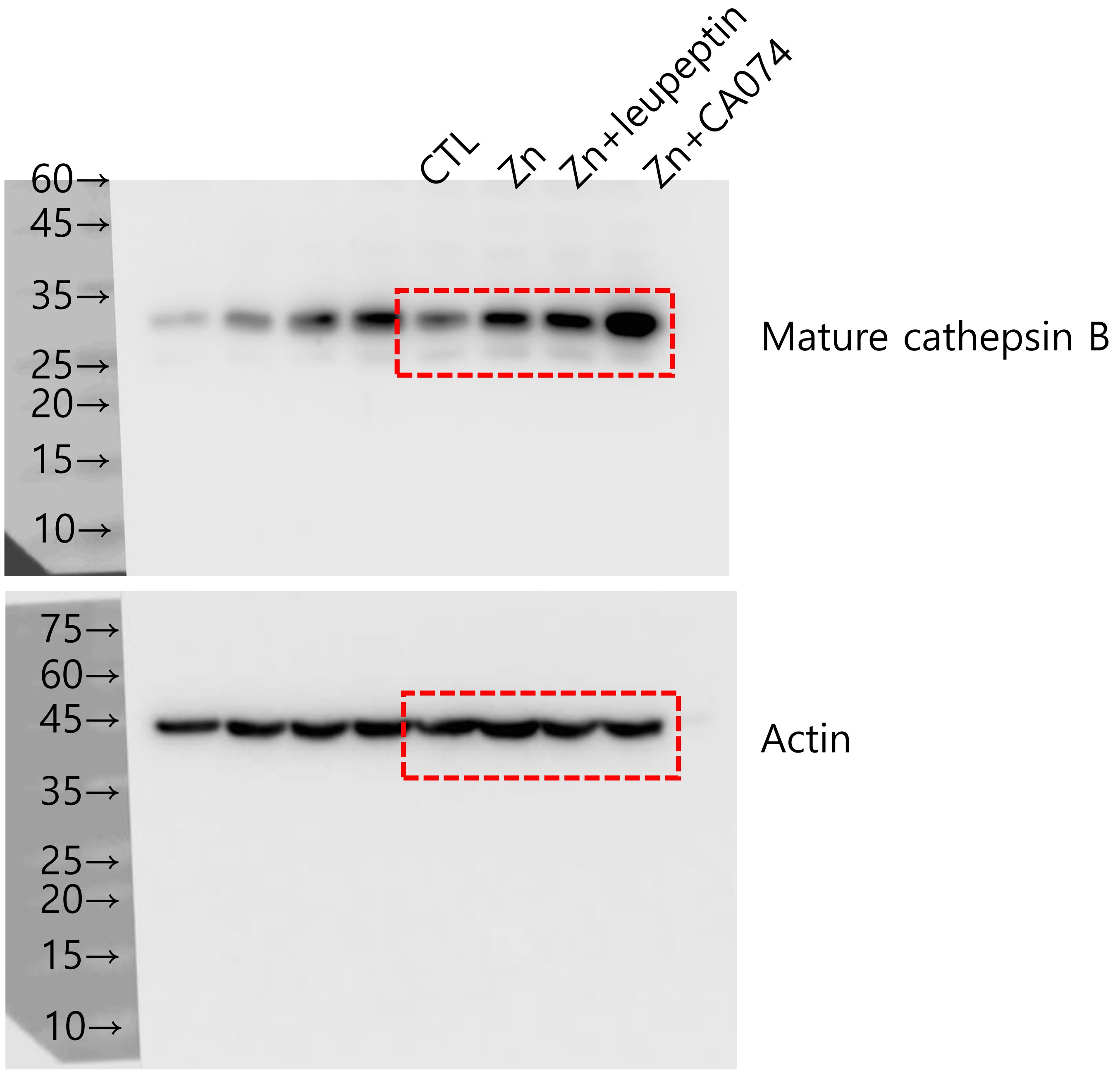

Figure 3D-Mature cathepsin B_leupeptin/CA074_#4

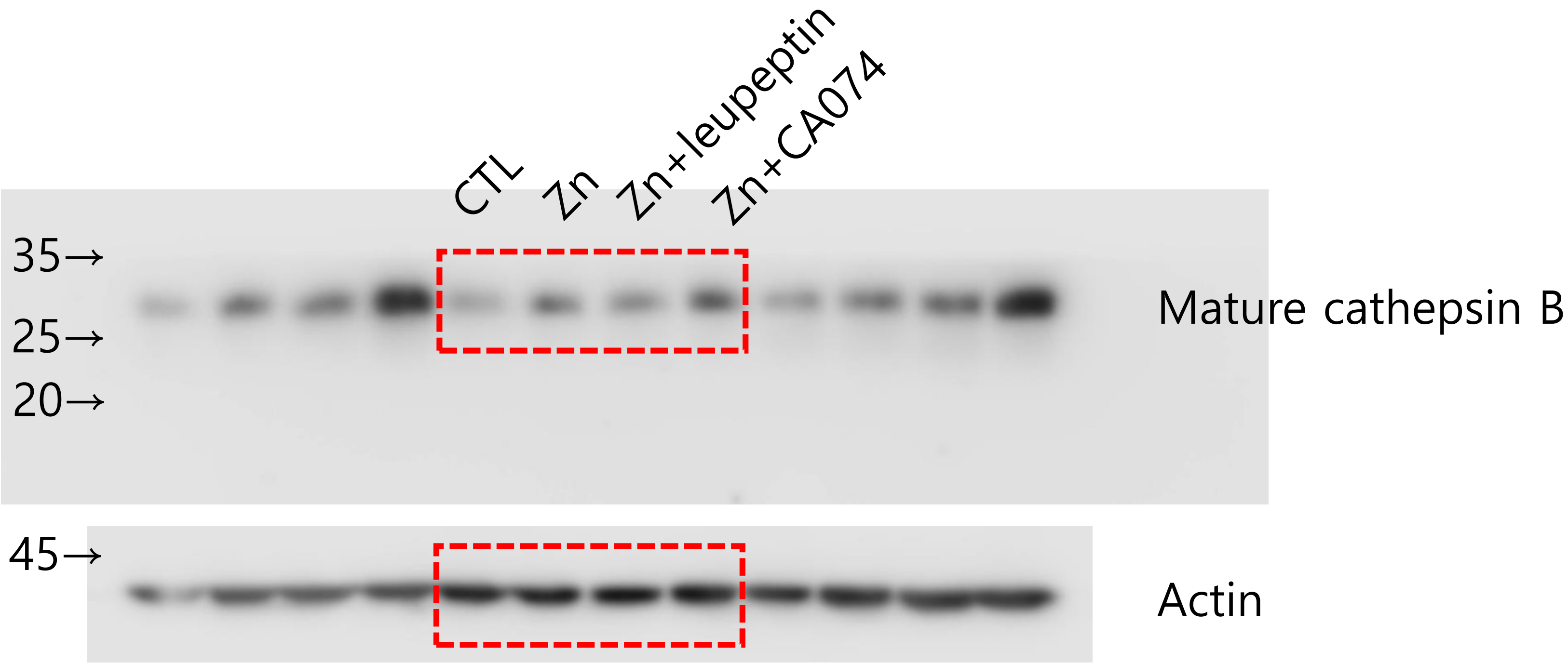

Figure 3D-Mature cathepsin B_leupeptin/CA074_#5

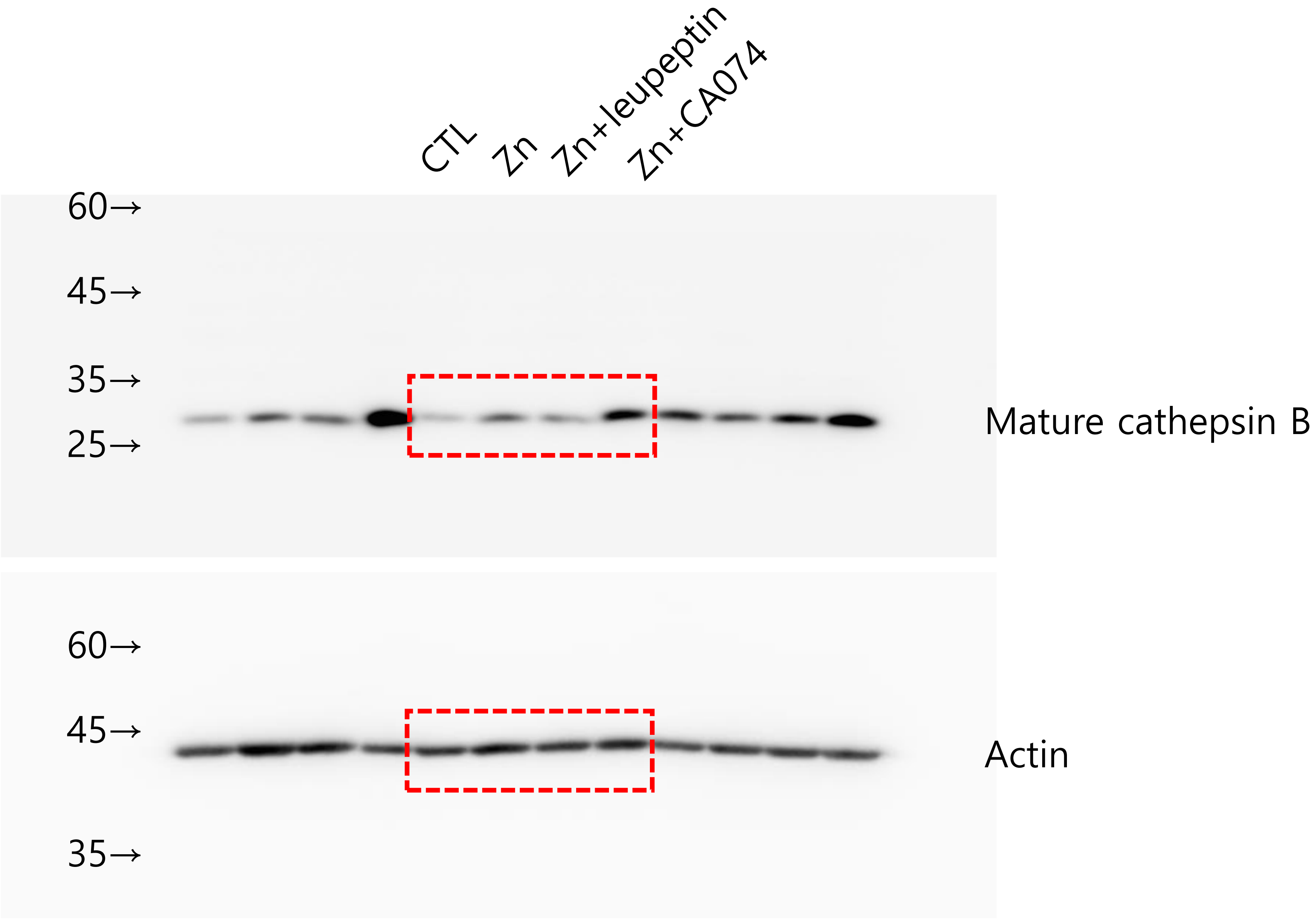

Figure 4B-EGFR_MβCD,CP_#1

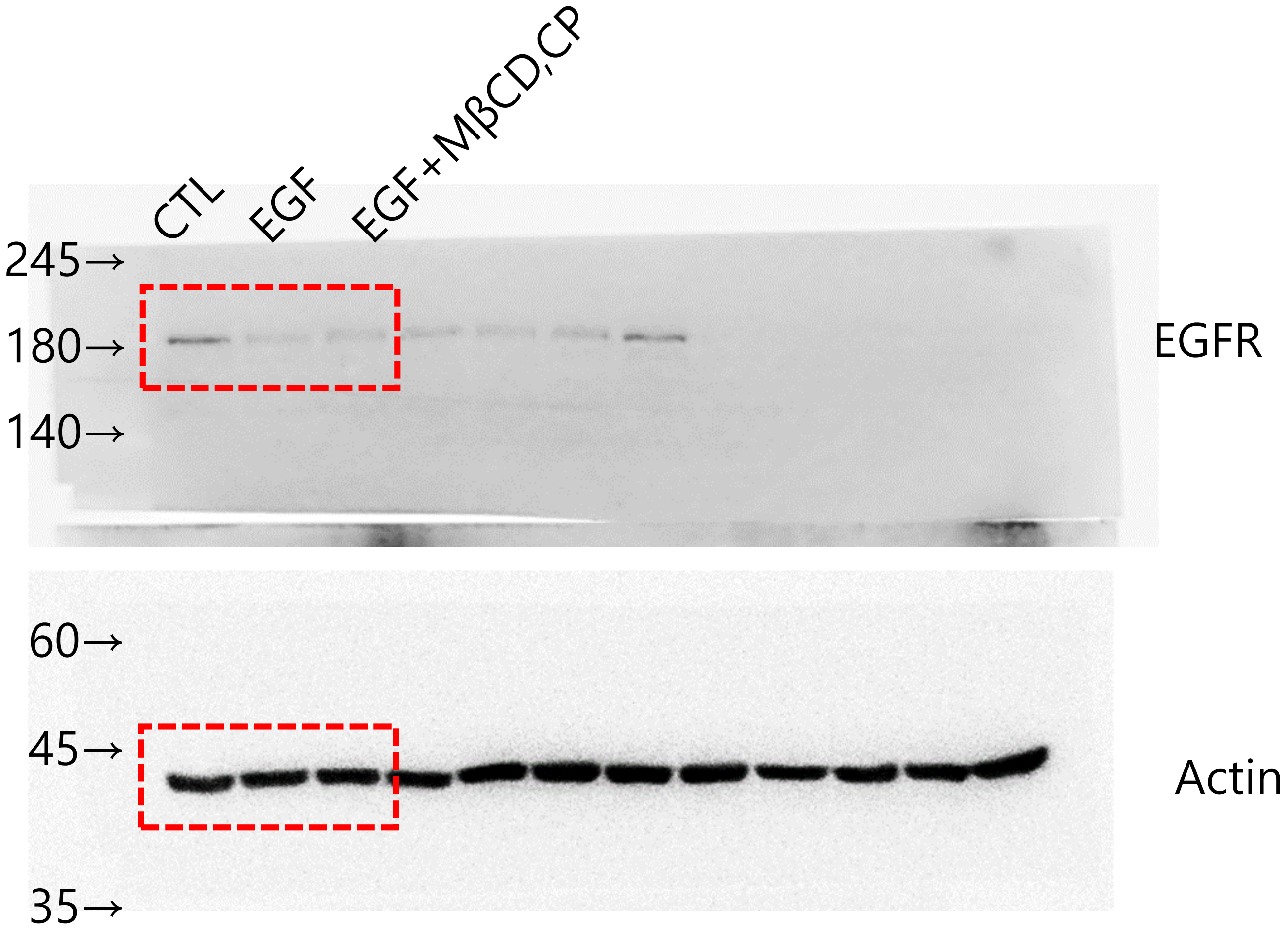

Figure 4B-EGFR_MβCD,CP_#2

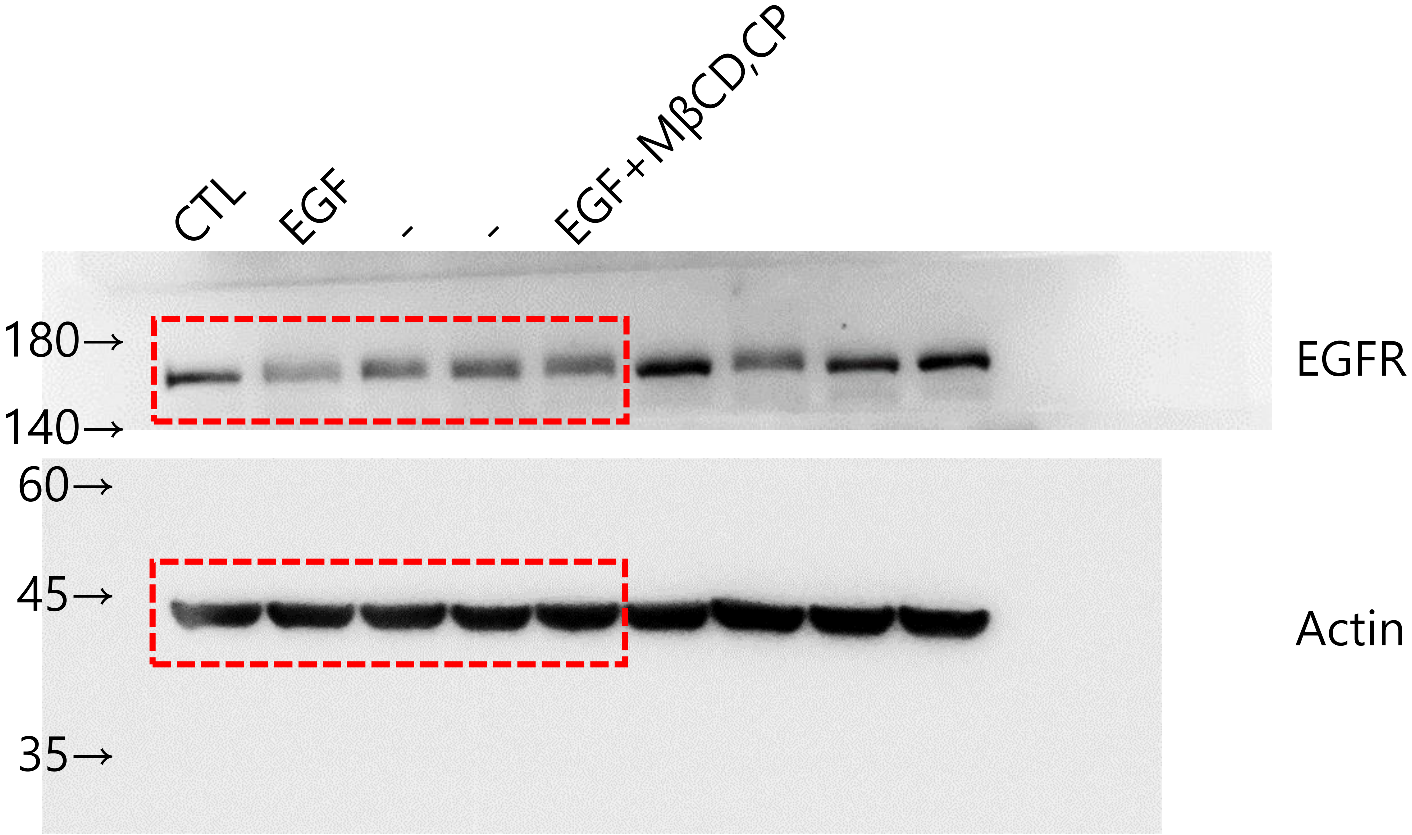

Figure 4B-EGFR_MβCD,CP_#3

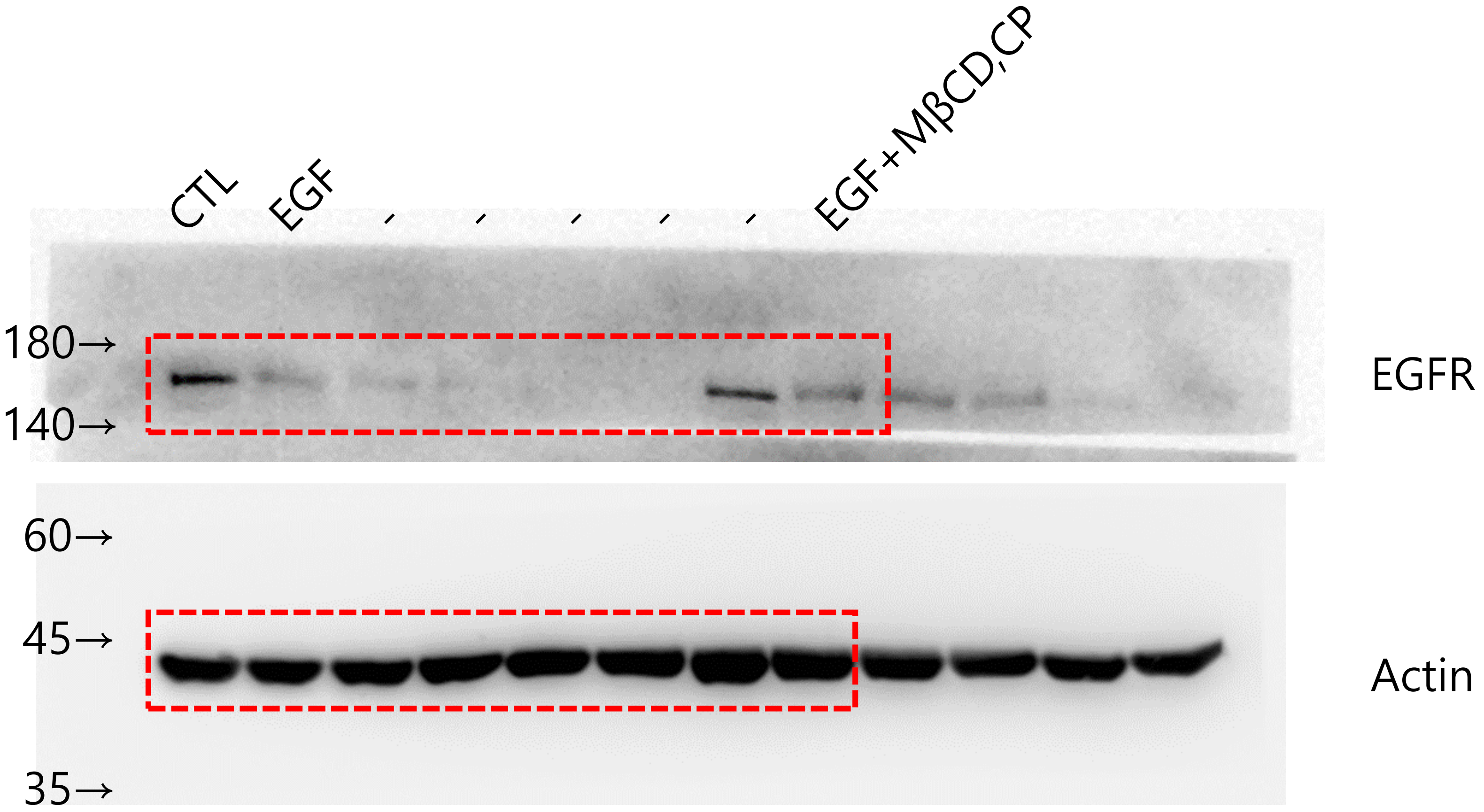

Figure 4B-EGFR_MβCD,CP_#4

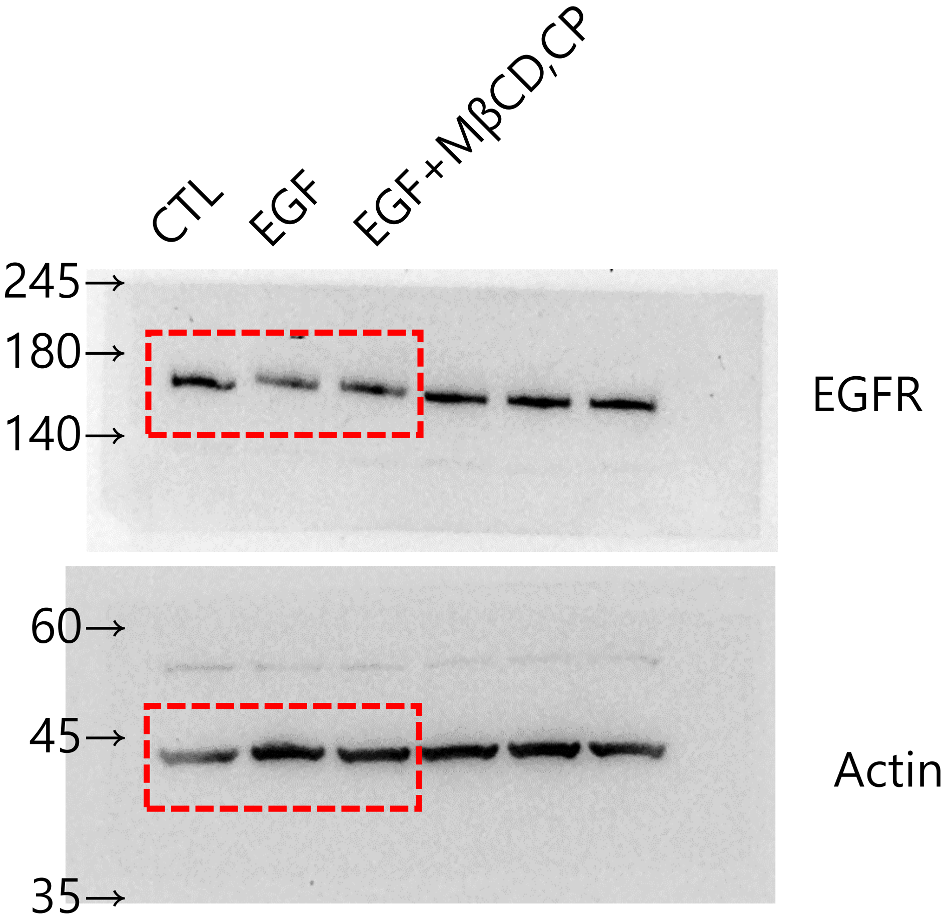

Figure 4B-EGFR_MβCD,CP_#5,6

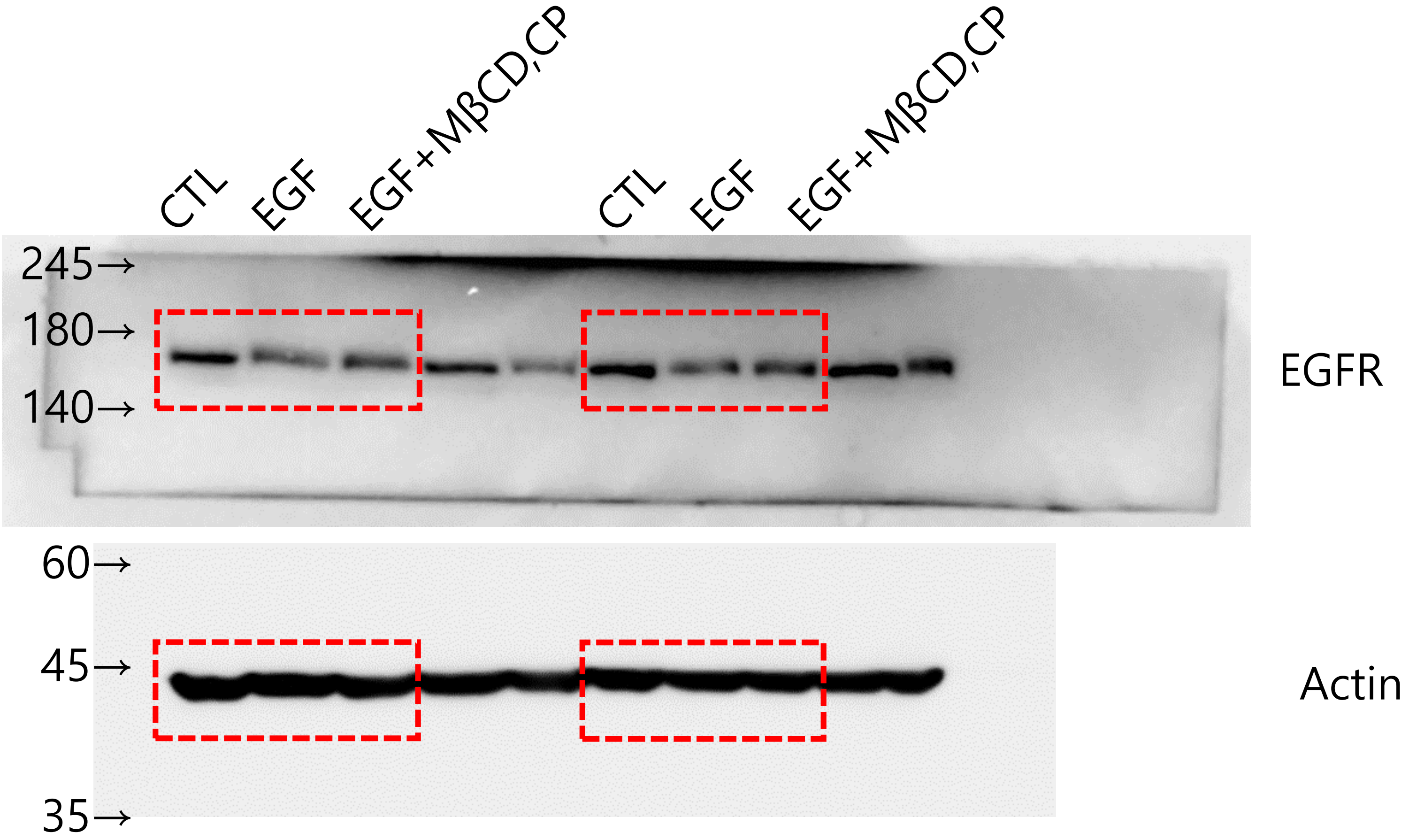

Figure 4B-EGFR_MβCD,CP_#7

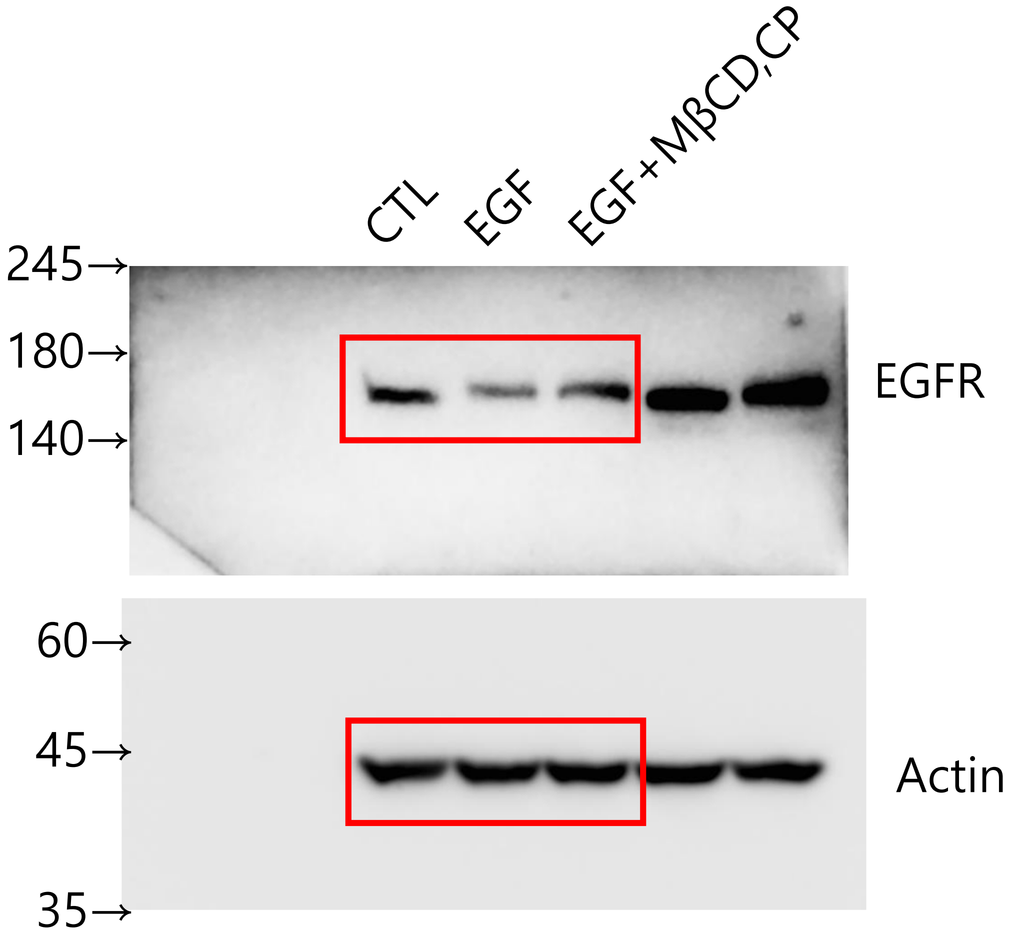

Figure 4B-EGFR_MβCD,CP_#8

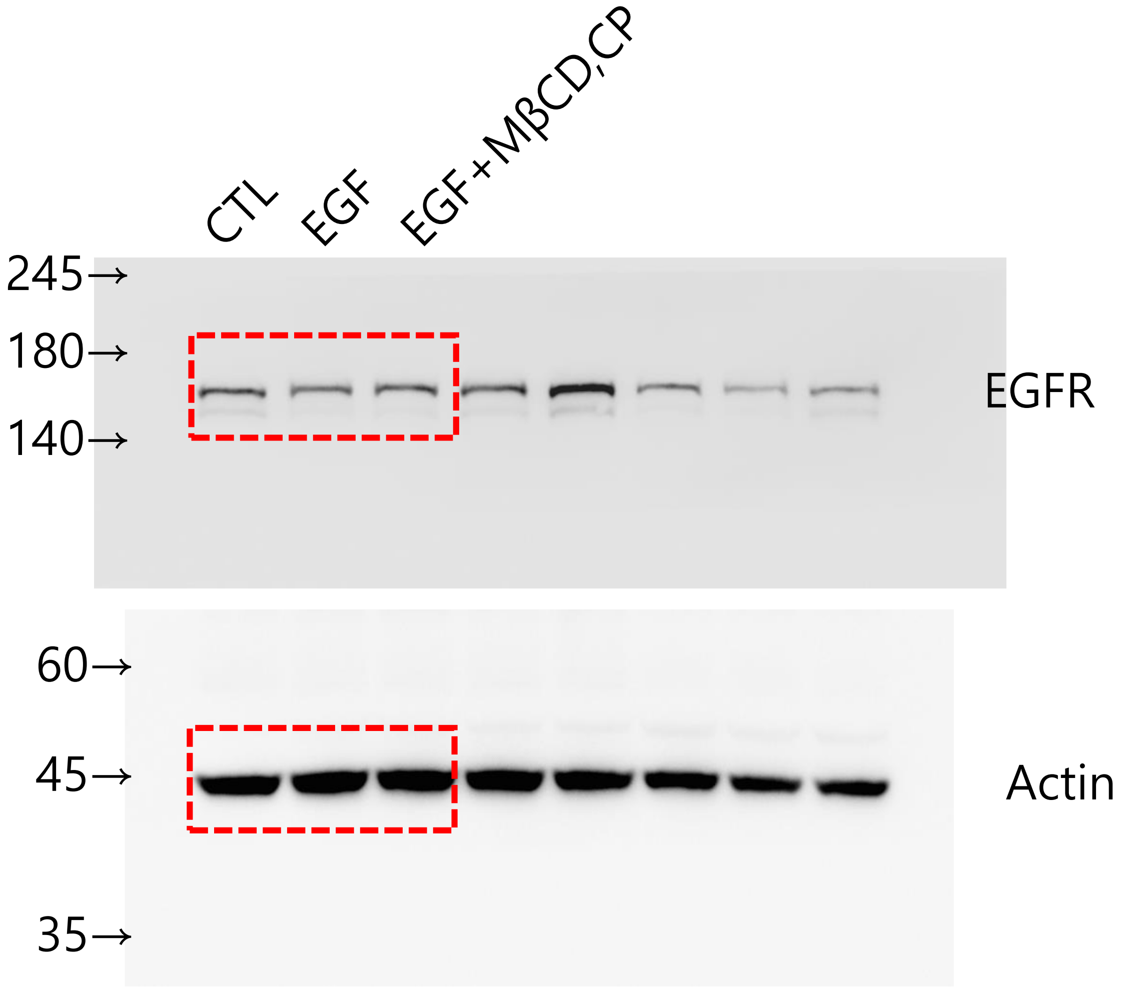

Figure 4B-LAMP-1_MβCD,CP_#1

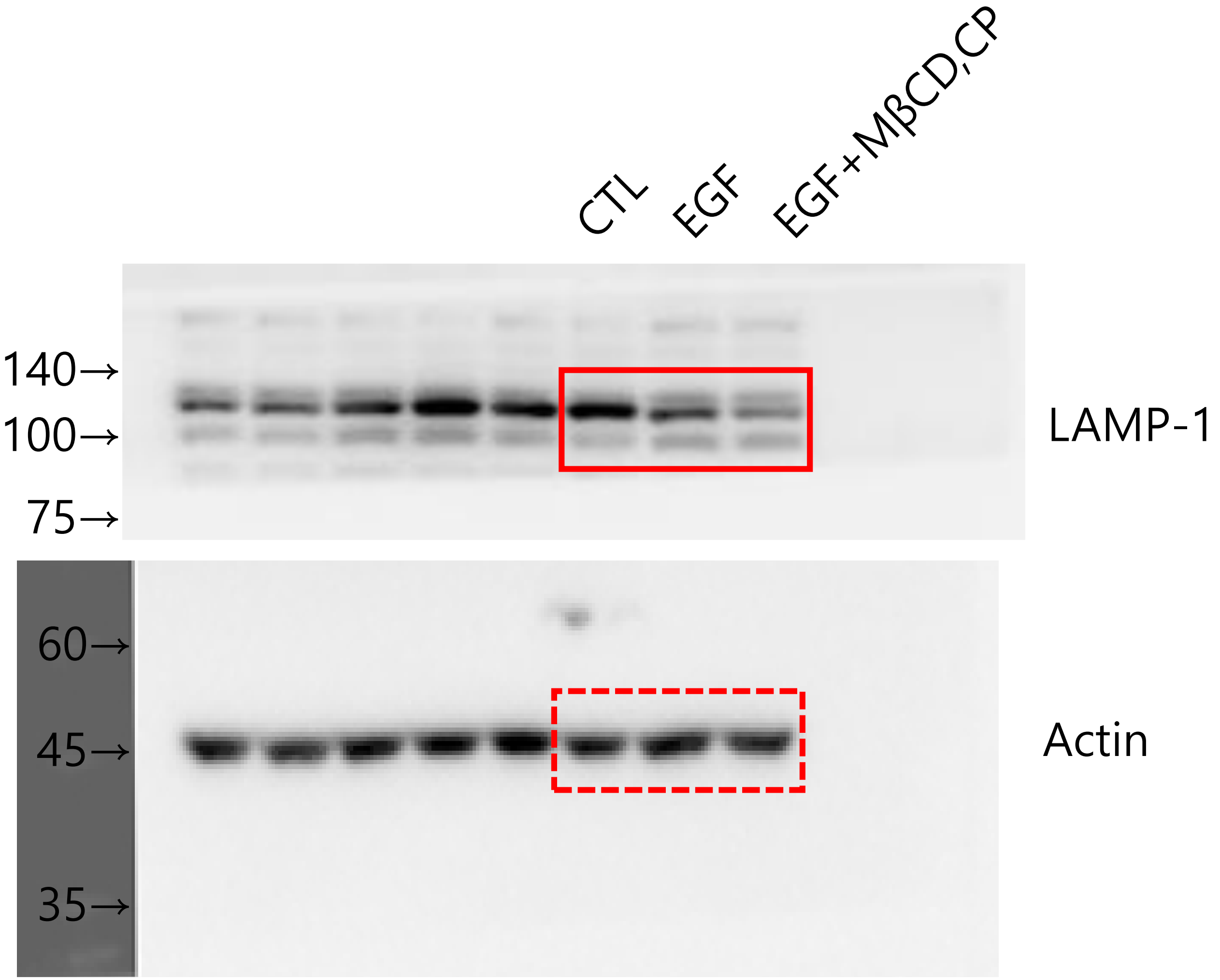

Figure 4B-LAMP-1_MβCD,CP_#2

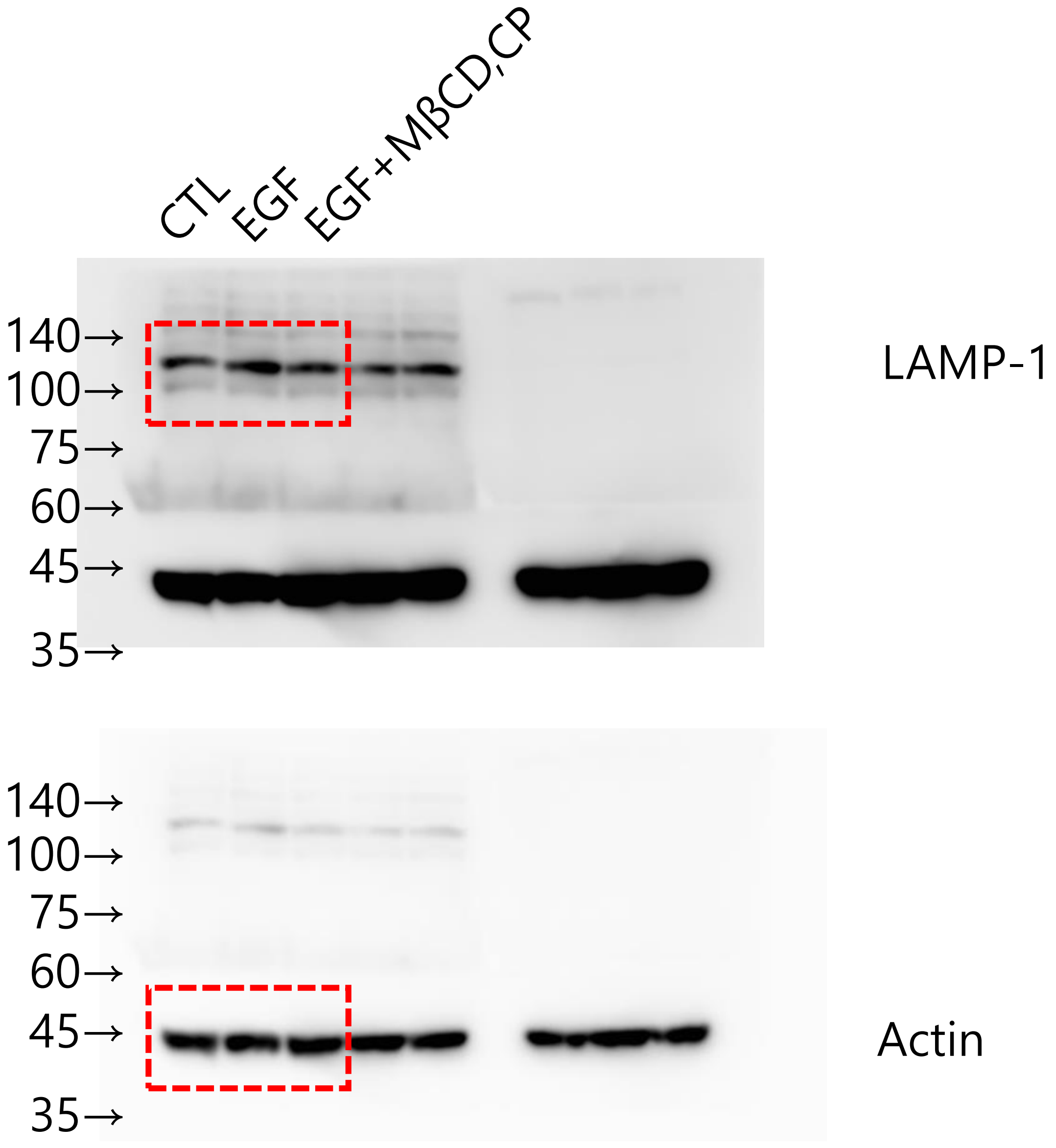

Figure 4B-LAMP-1_MβCD,CP_#3

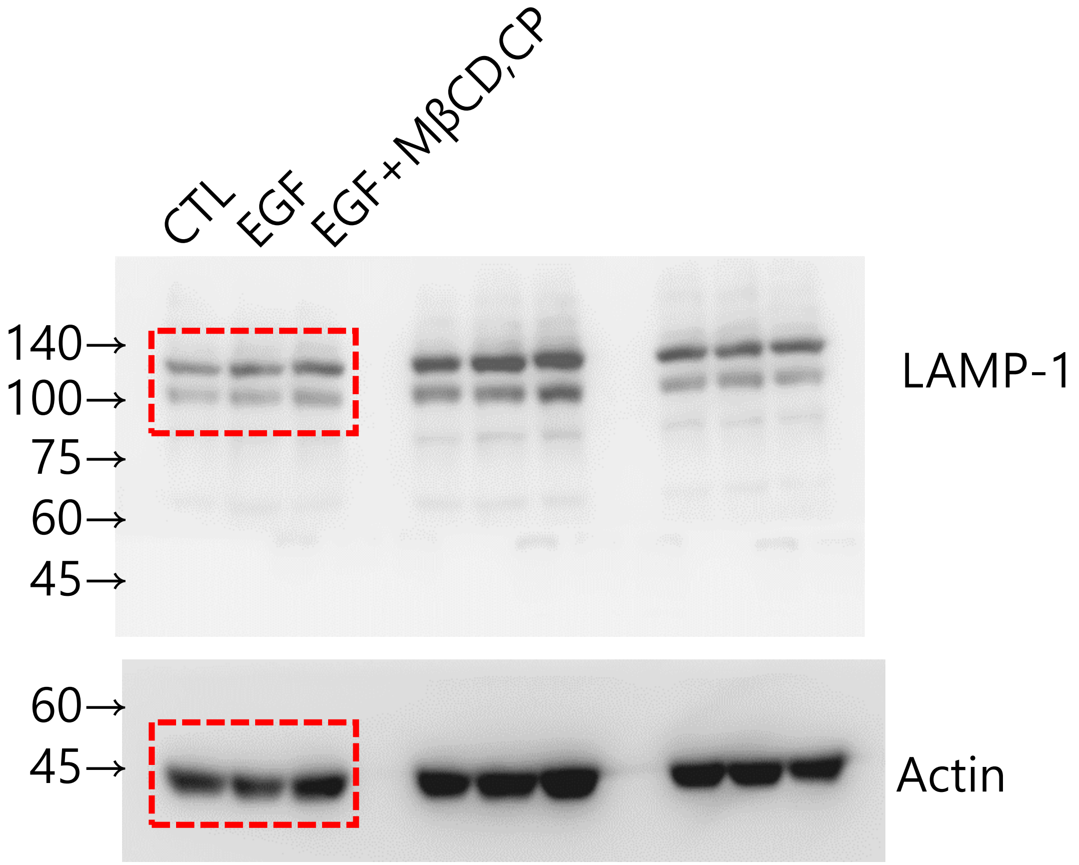

Figure 4B-EGFR_Cpd56_#1

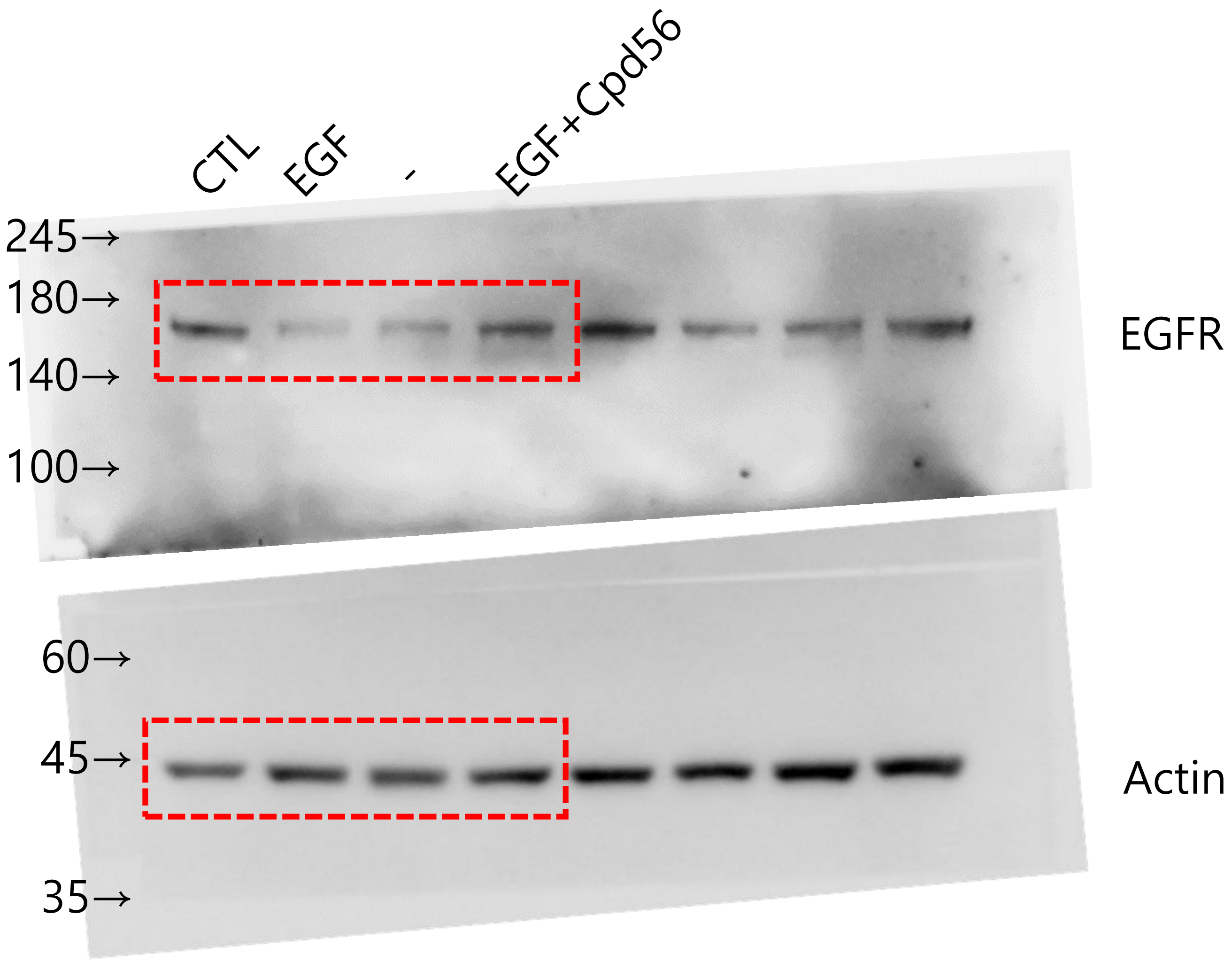

Figure 4B-EGFR_Cpd56_#2

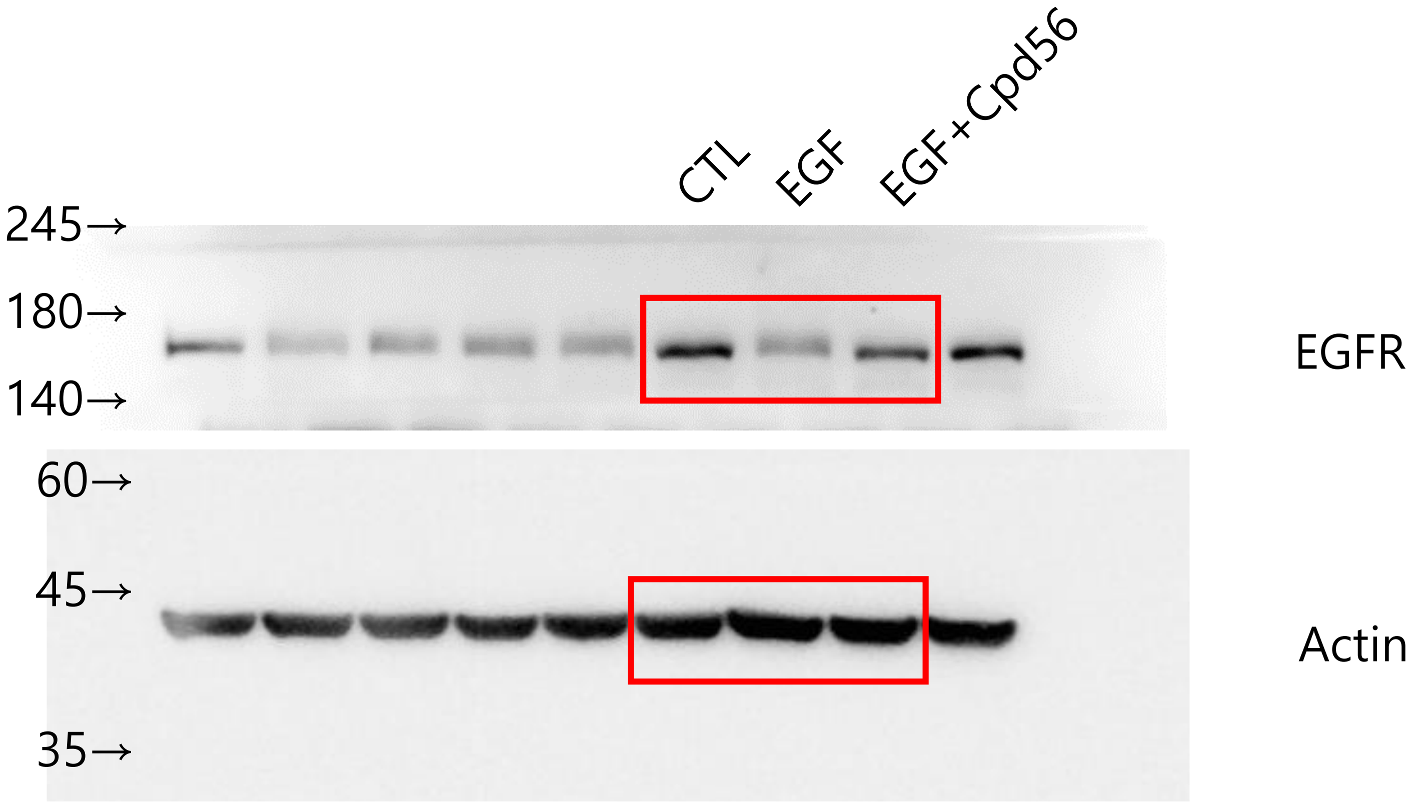

Figure 4B-EGFR_Cpd56_#3

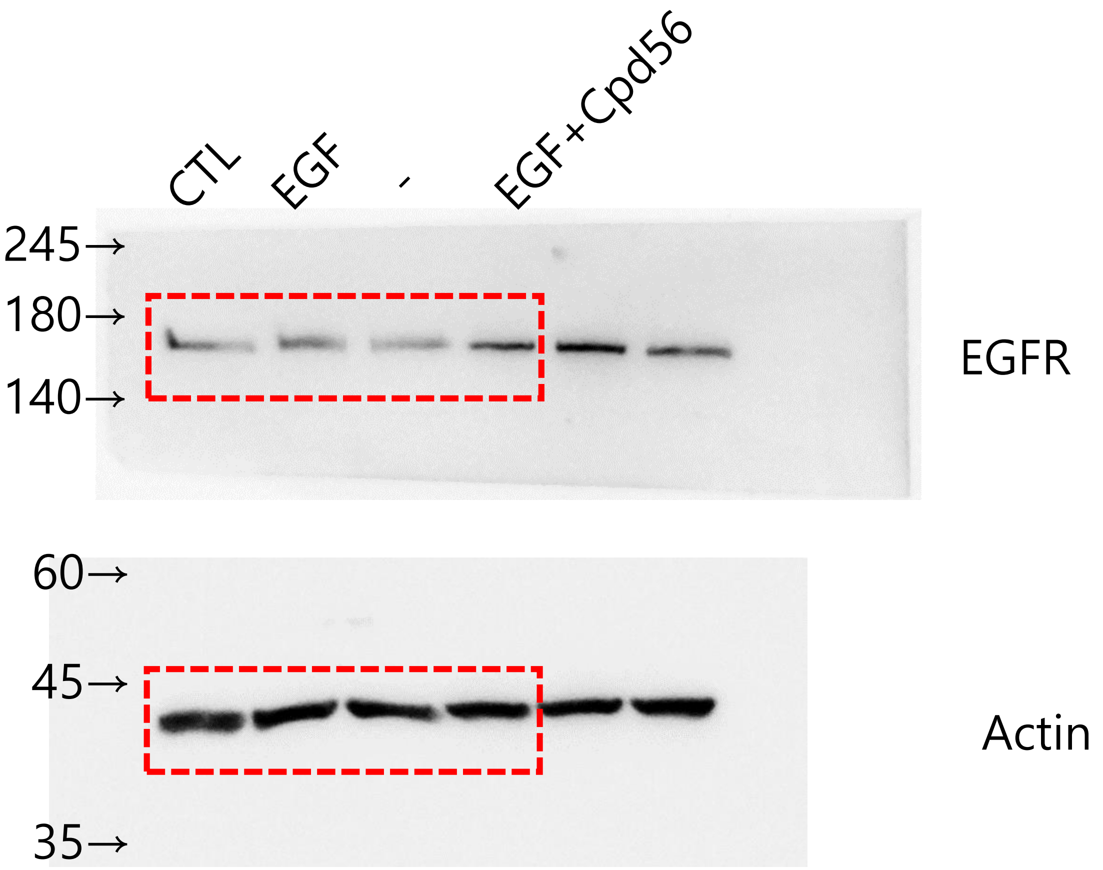

Figure 4B-EGFR_Cpd56_#4

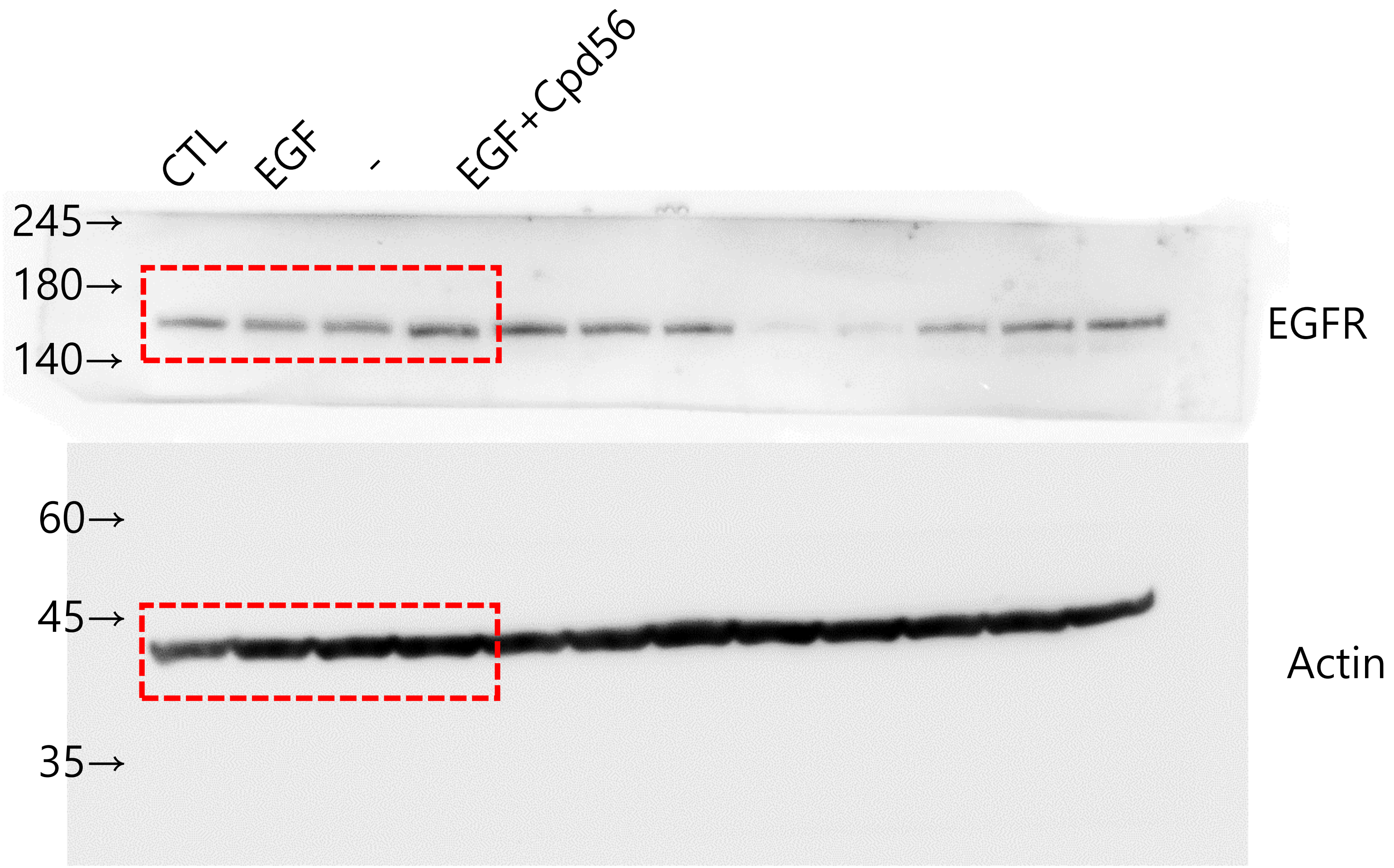

Figure 4B-EGFR_Cpd56_#5

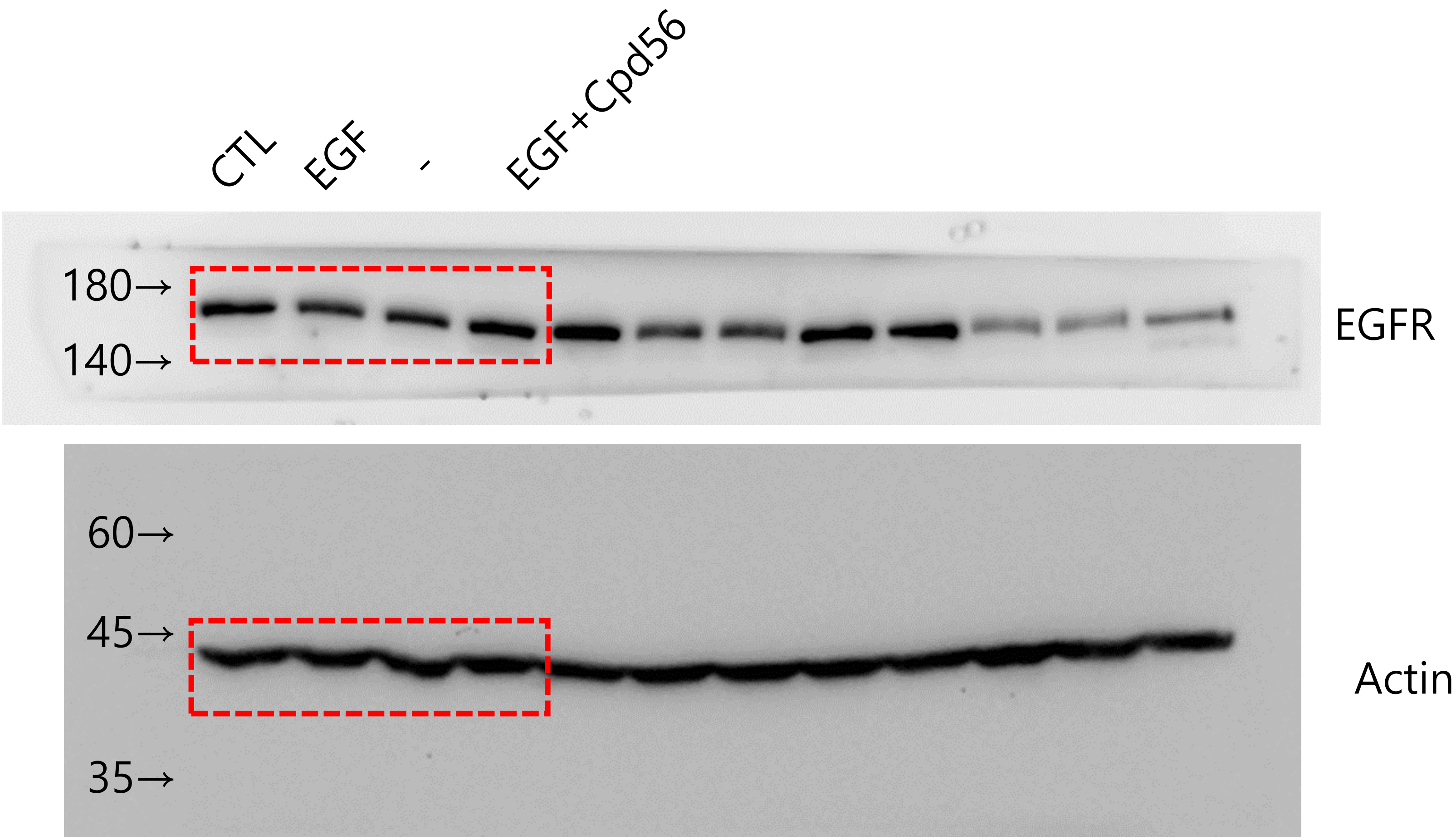

Figure 4B-EGFR_Cpd56_#6

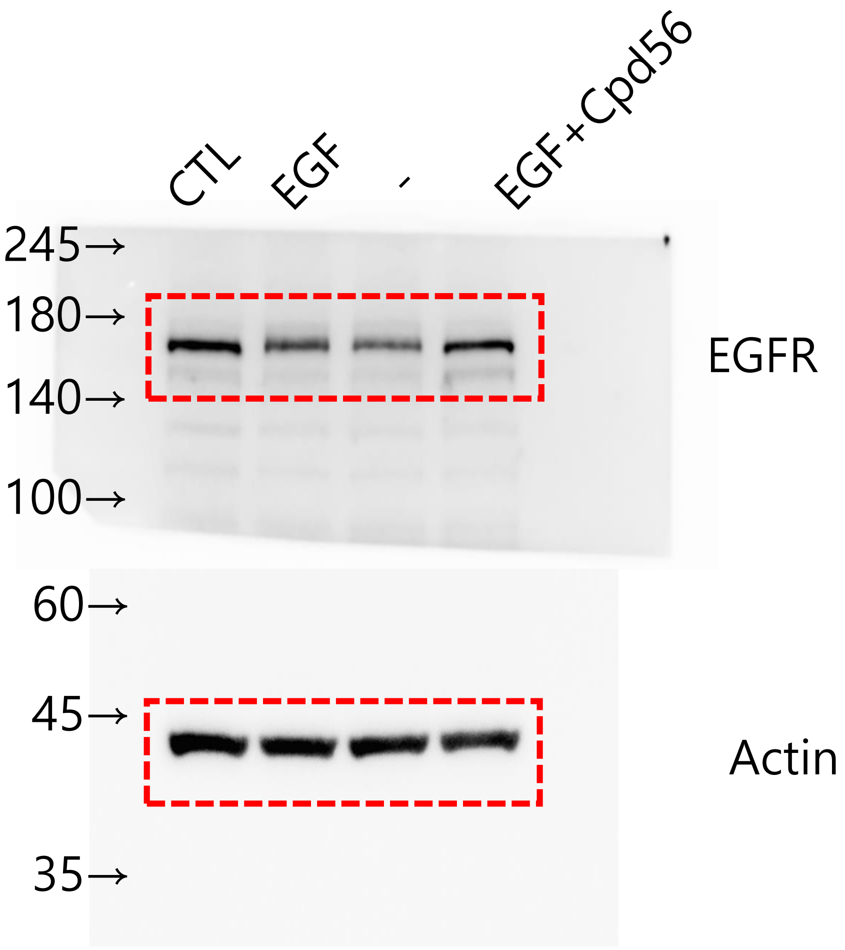

Figure 4B-EGFR_Cpd56_#7

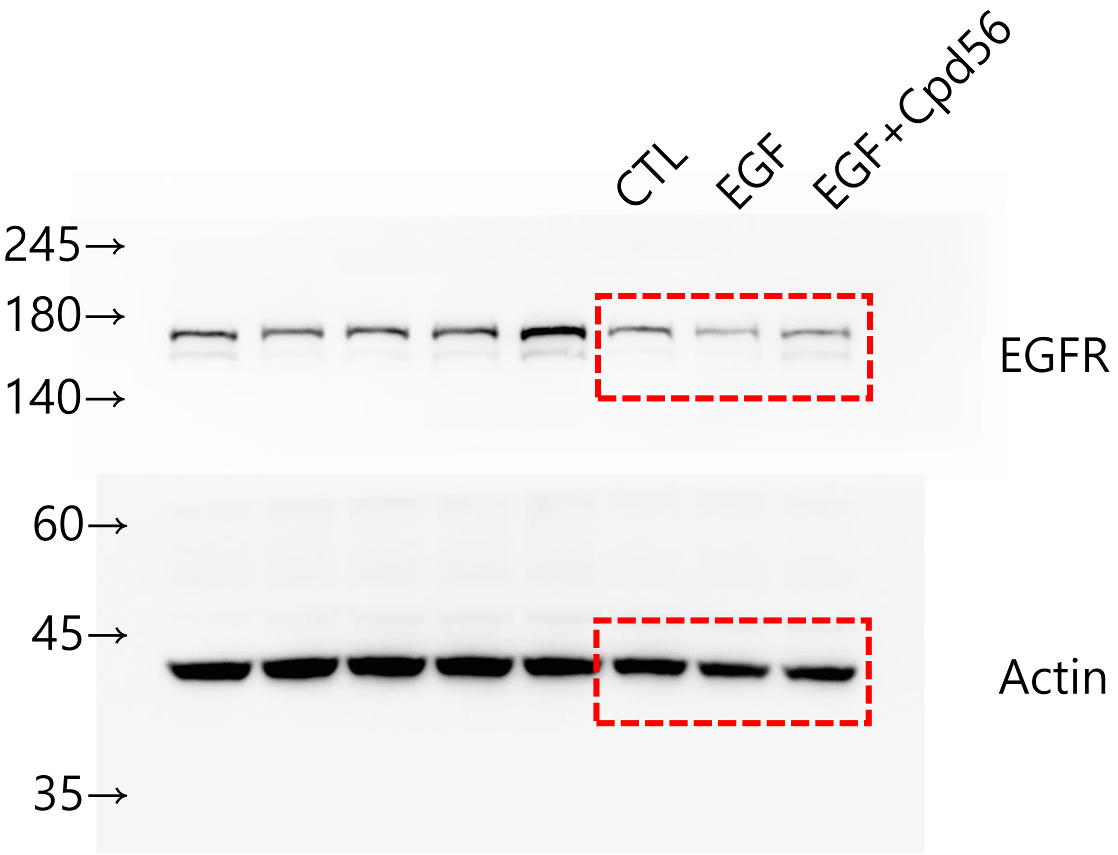

Figure 4B-LAMP-1_Cpd56_#1

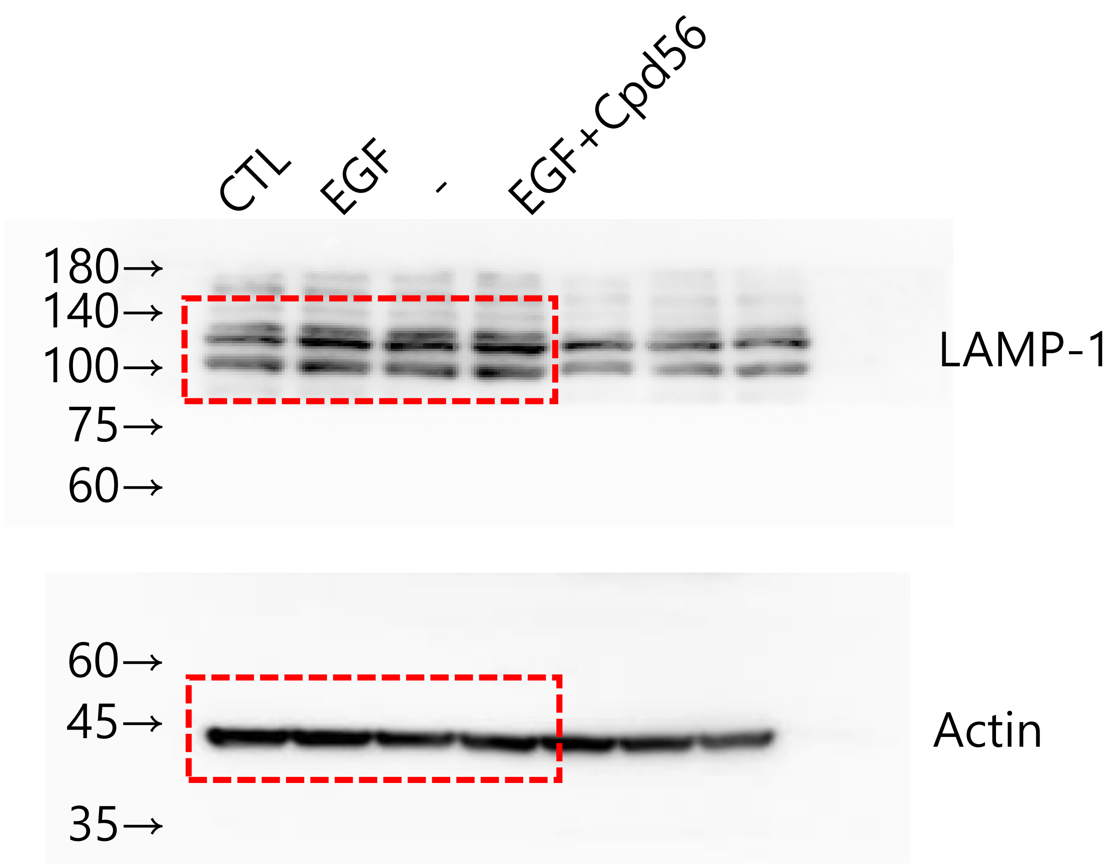

Figure 4B-LAMP-1_Cpd56_#2

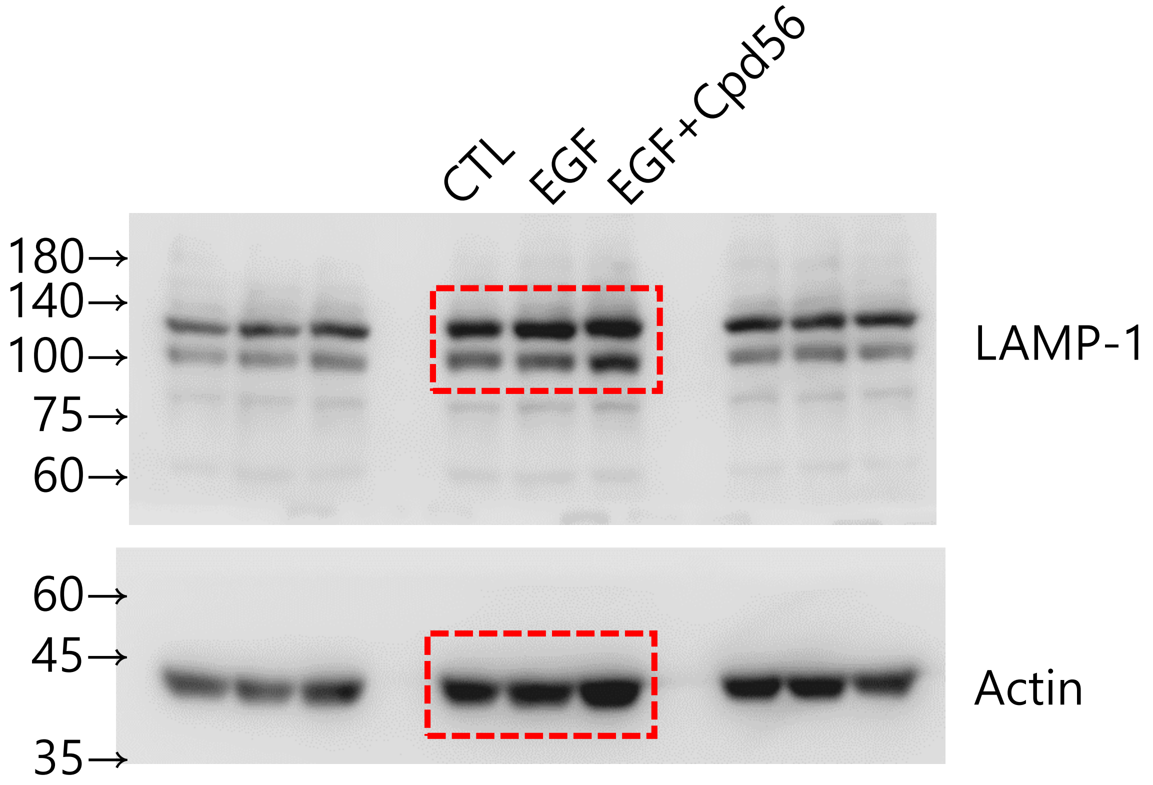

Figure 4B-LAMP-1_Cpd56_#3,4

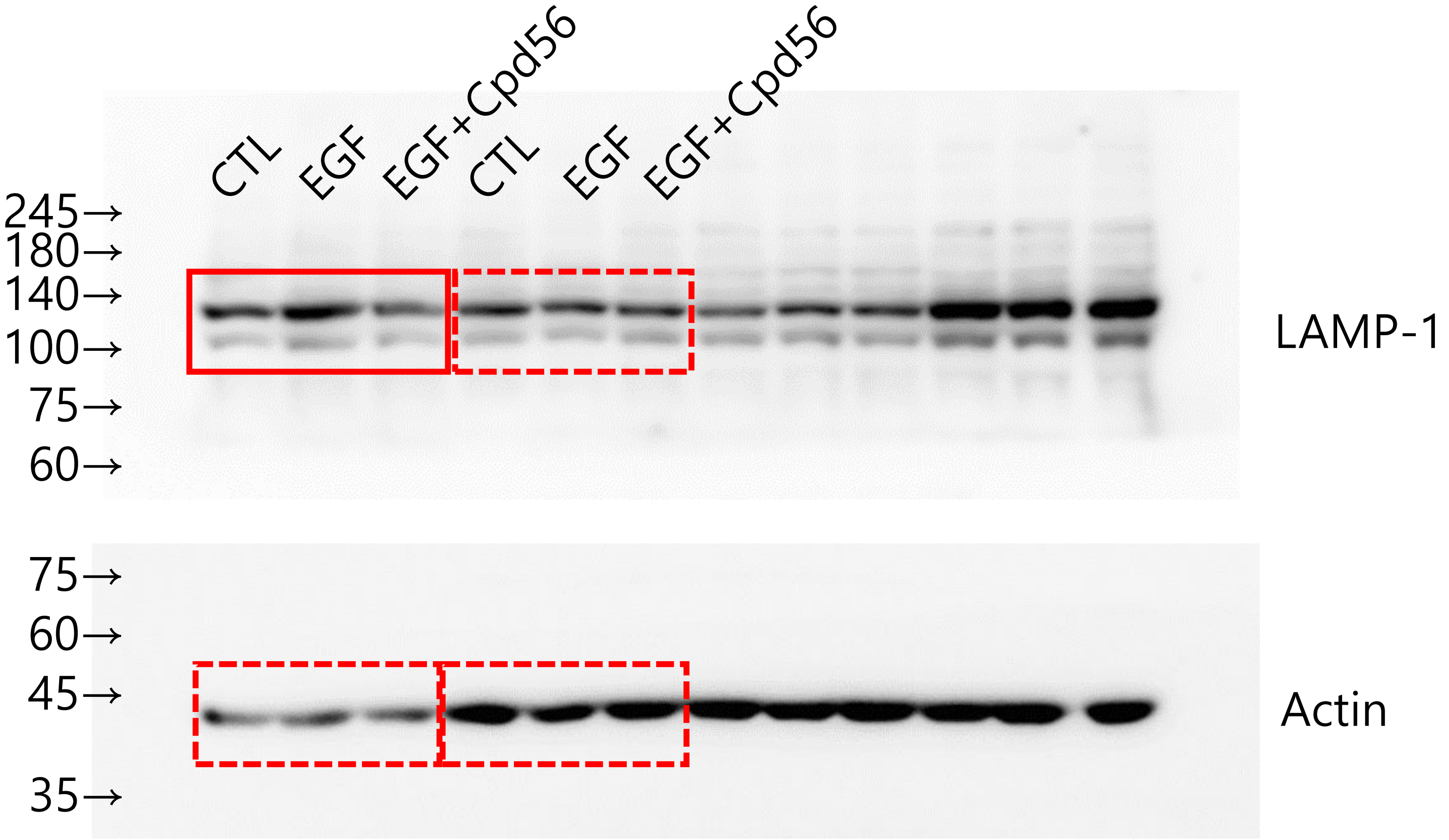

Figure 4B-LAMP-1_Cpd56_#5

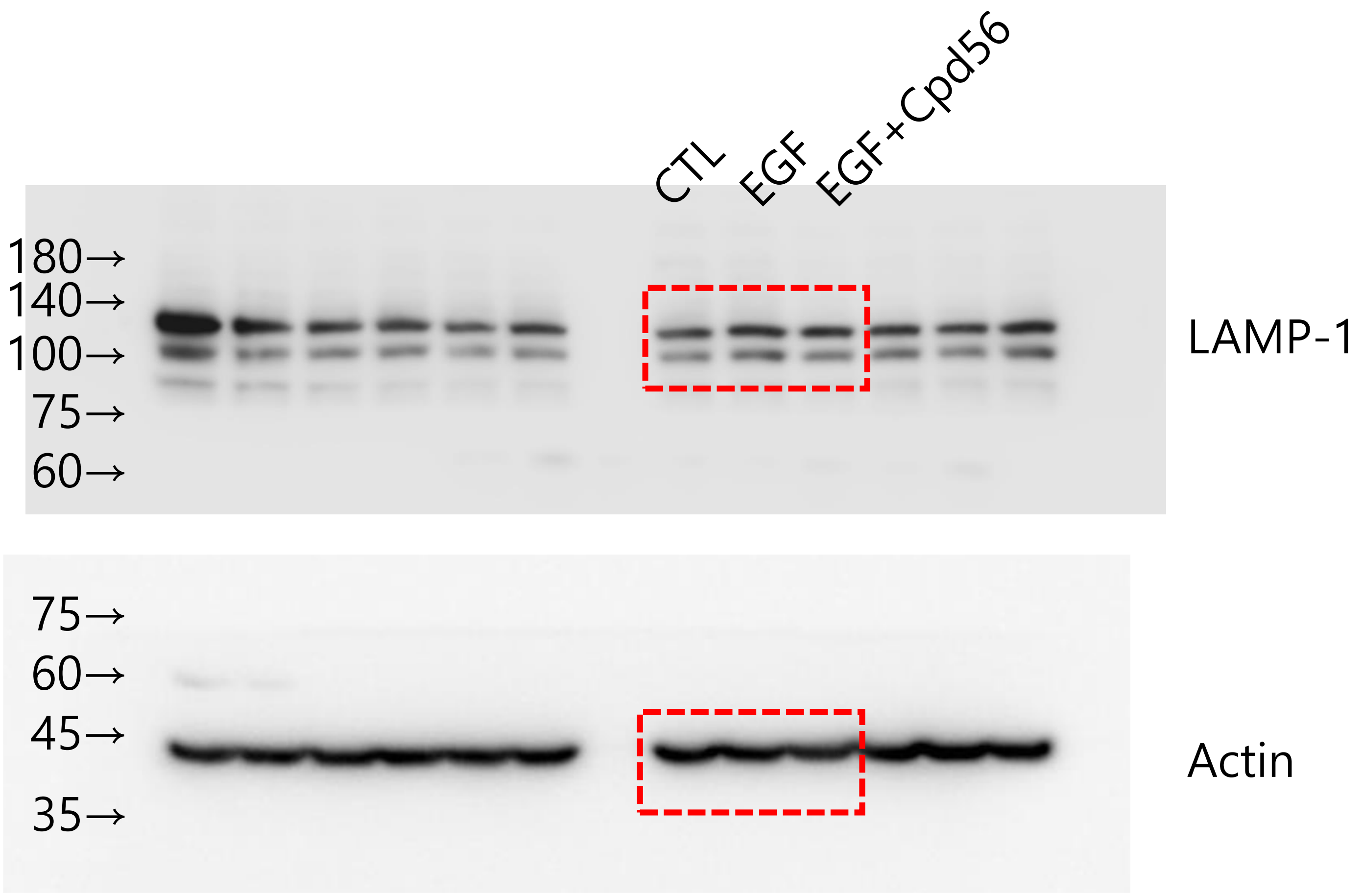

Figure 4B-EGFR_Ciliob_#1

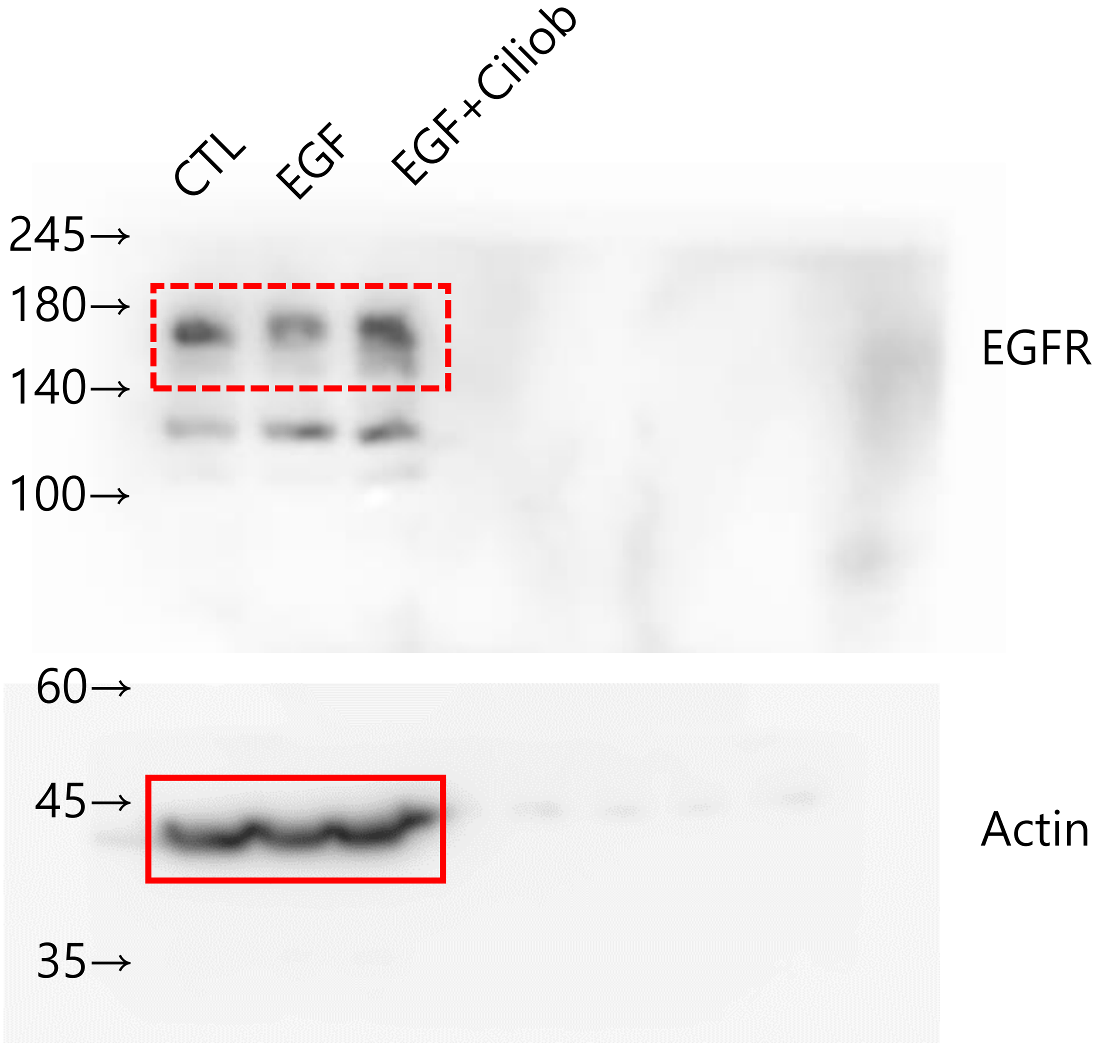

Figure 4B-EGFR_Ciliob_#2

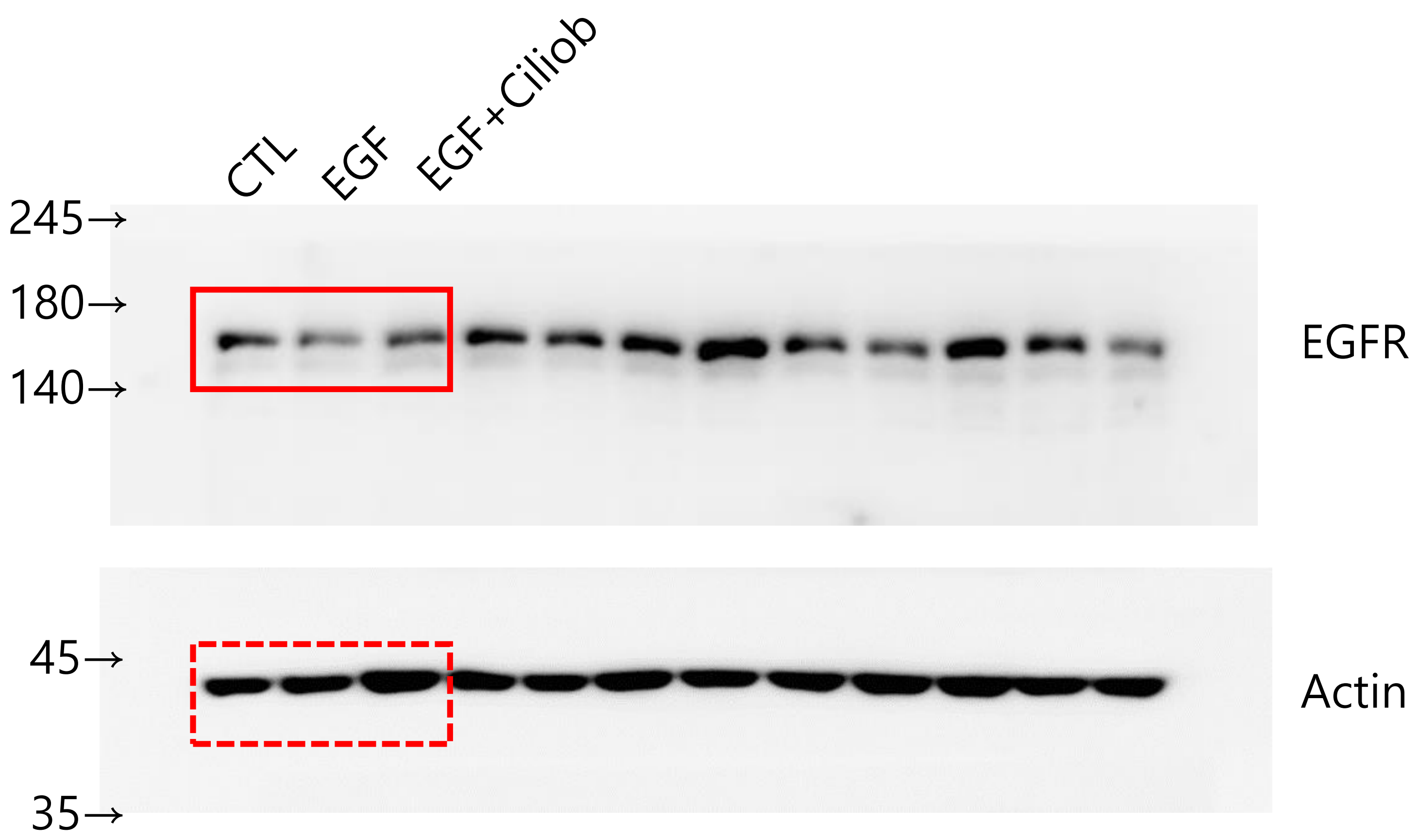

Figure 4B-EGFR_Ciliob_#3

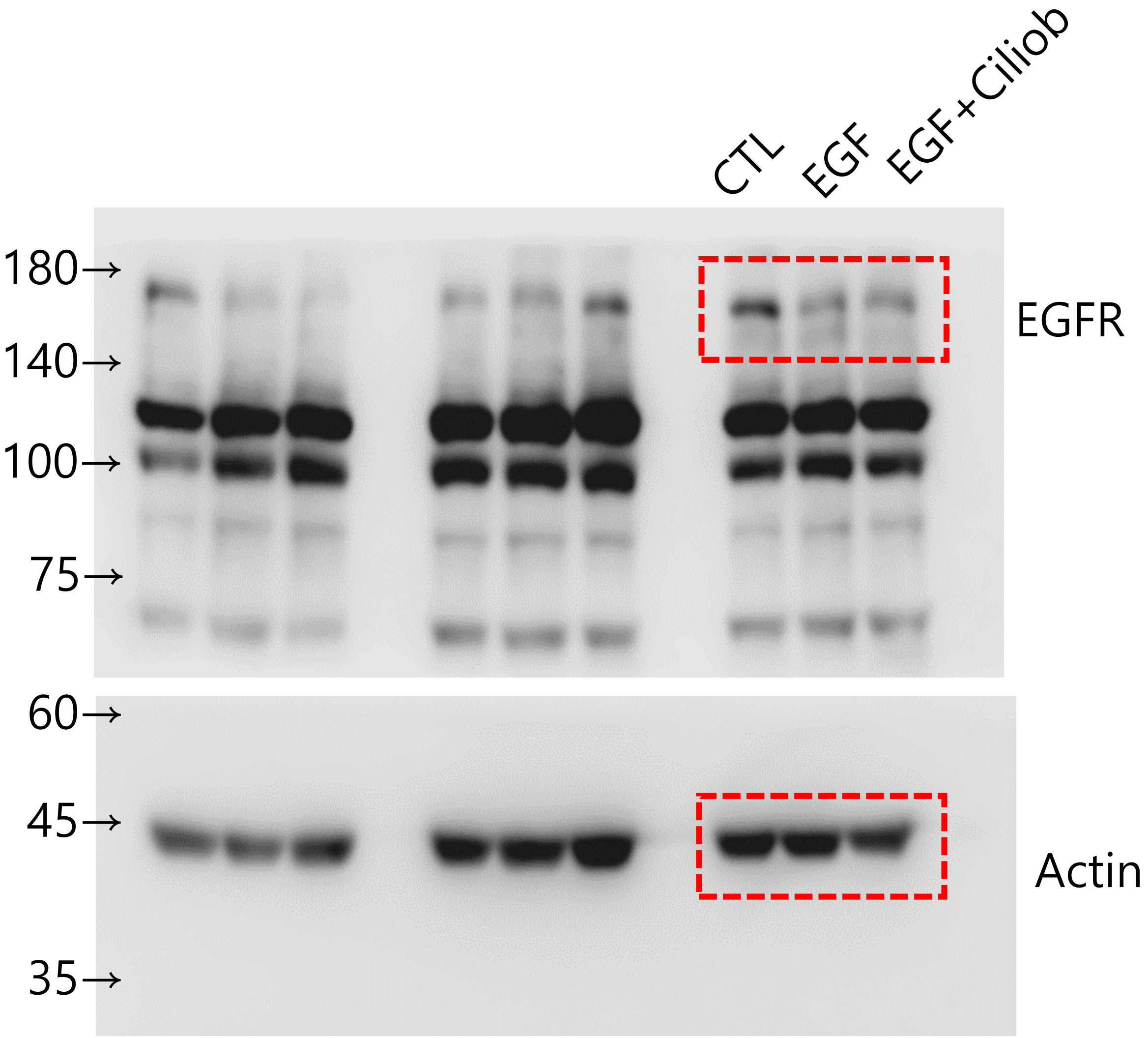

Figure 4B-EGFR_Ciliob_#4

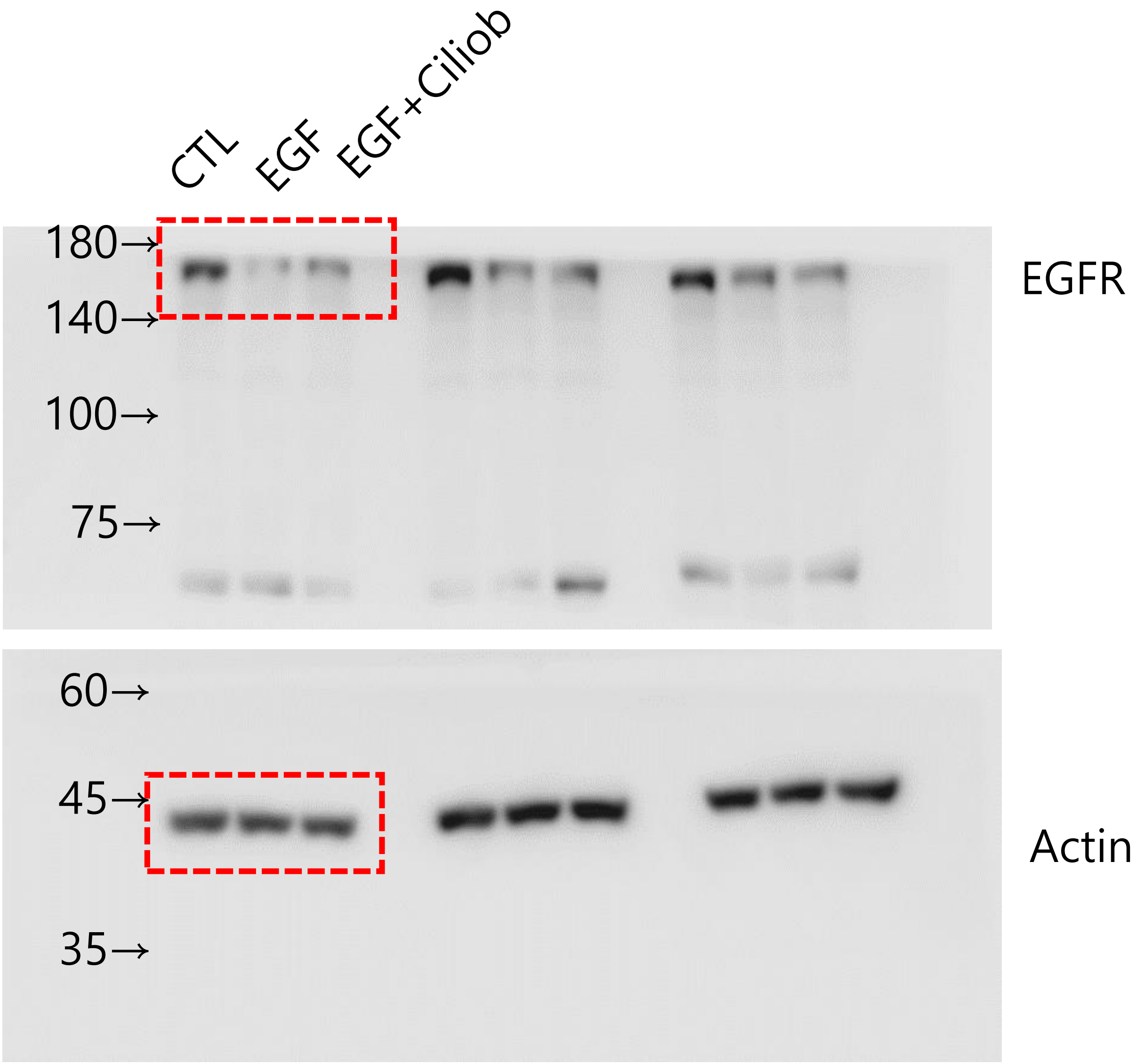

Figure 4B_LAMP-1_Ciliob_#1,2

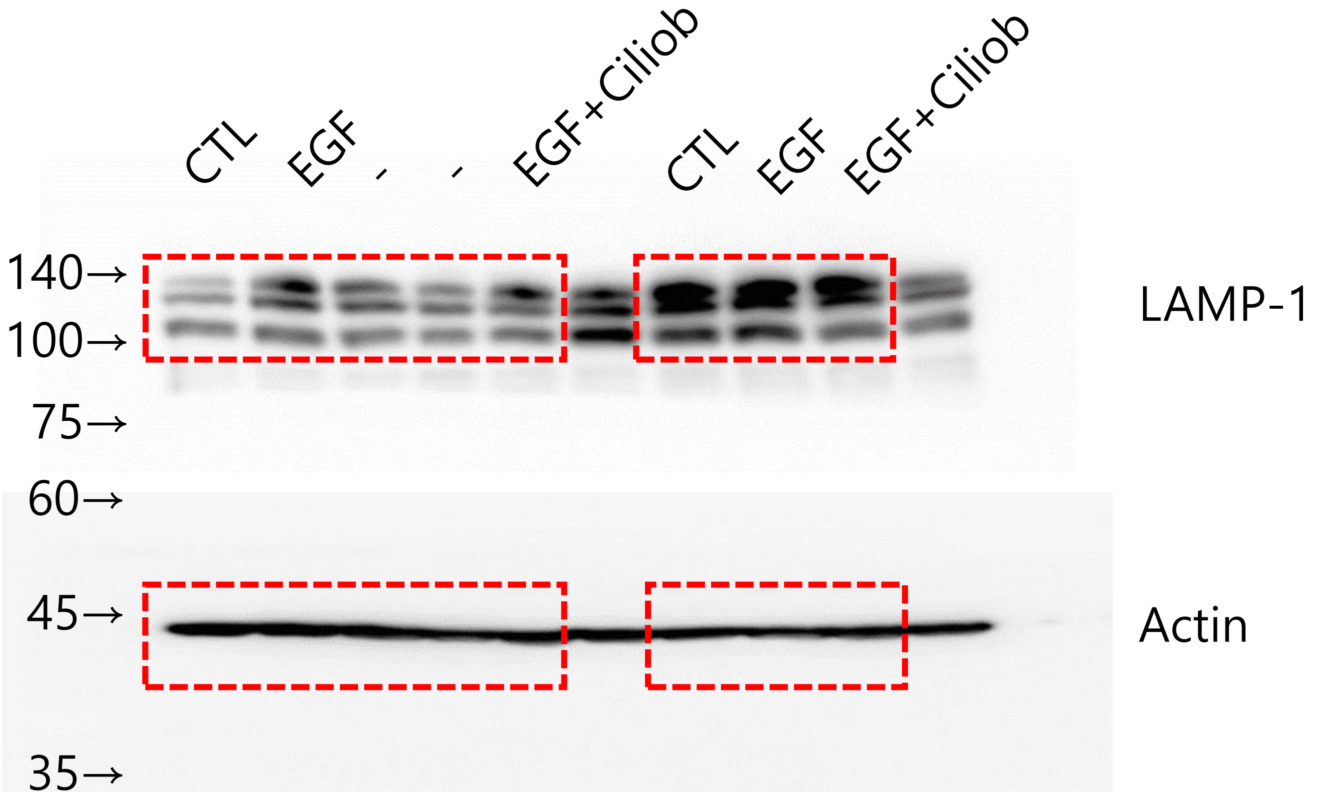

Figure 4B_LAMP-1_Ciliob_#3

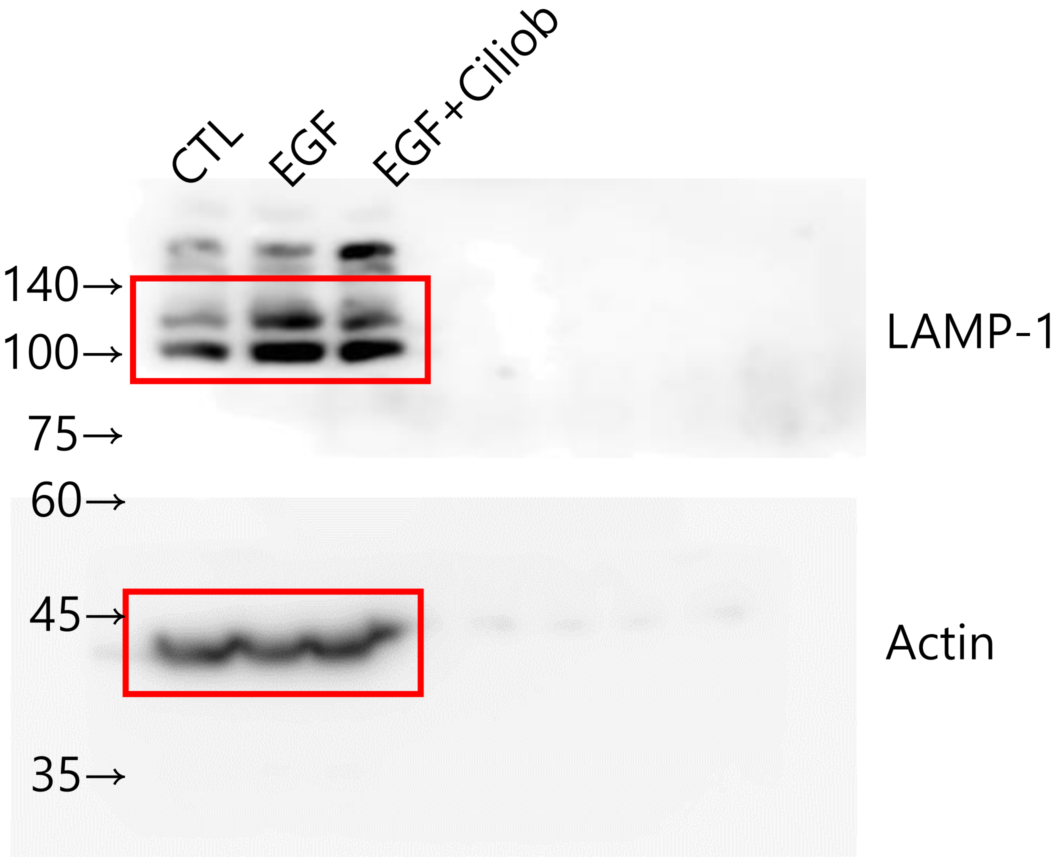

Figure 4B_LAMP-1_Ciliob_#4,5

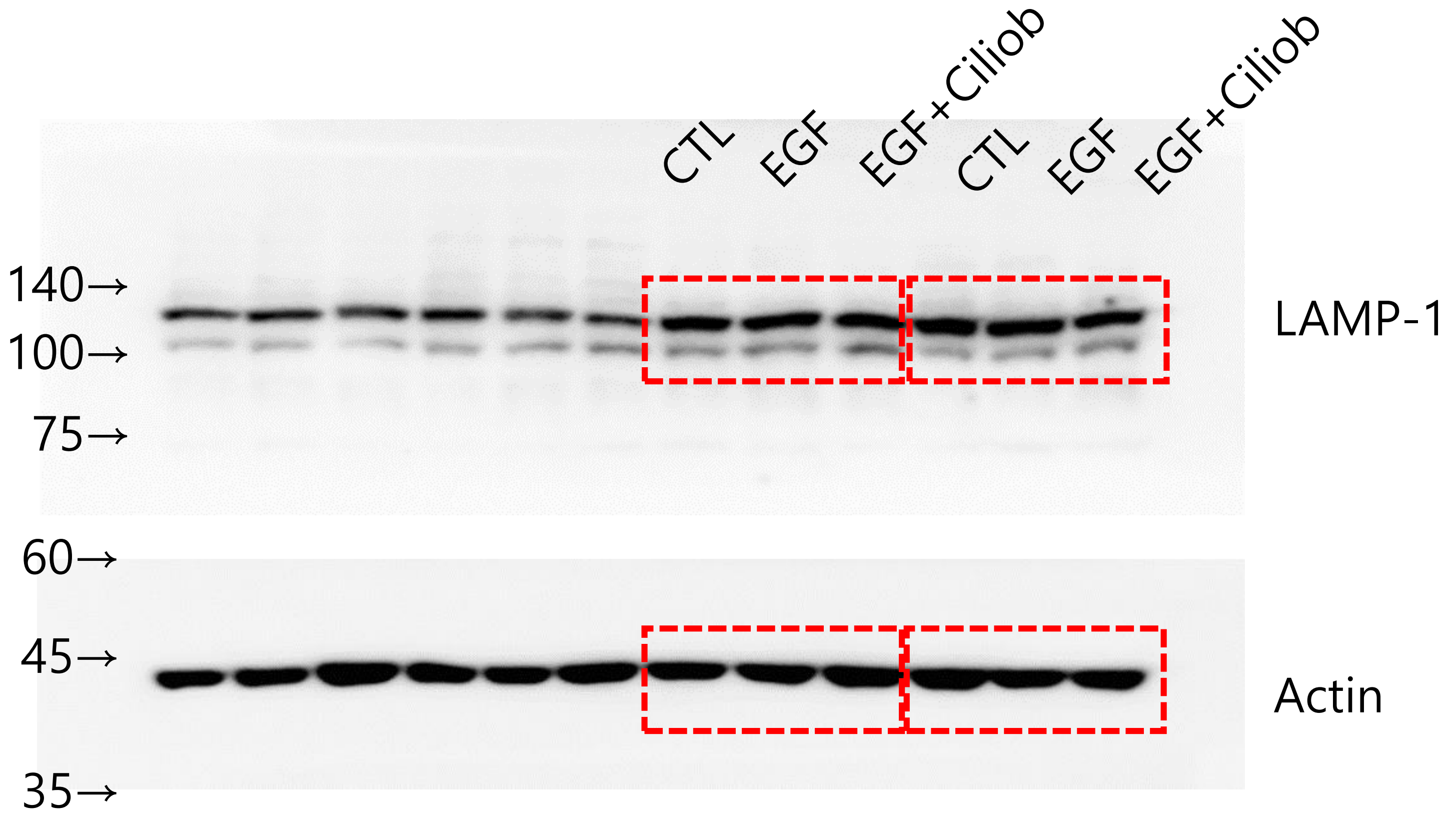

Figure 4D_Mature cathepsin B_MβCD,CP/Cpd56_#1

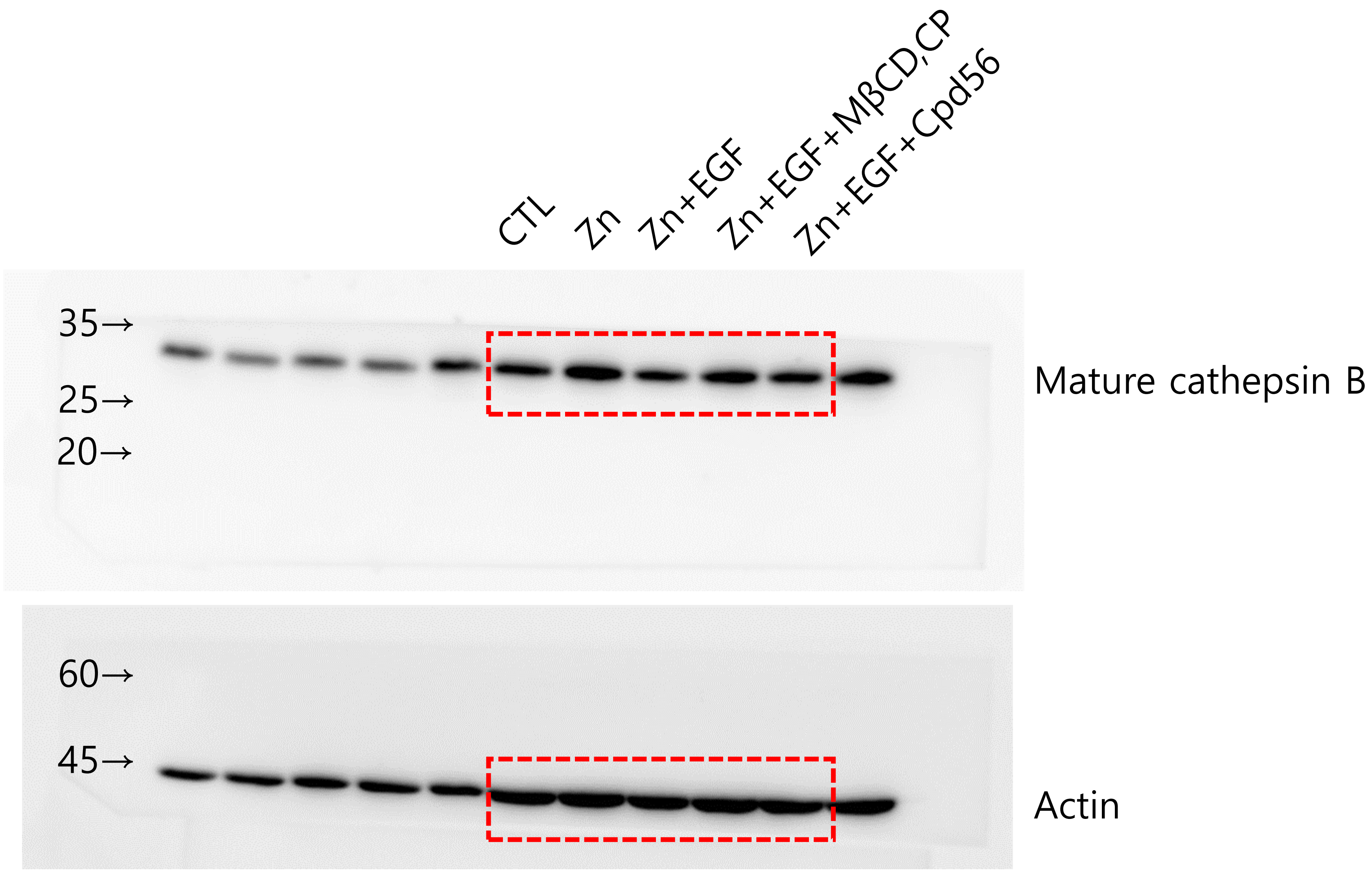

Figure 4D_Mature cathepsin B_MβCD,CP/Cpd56_#2

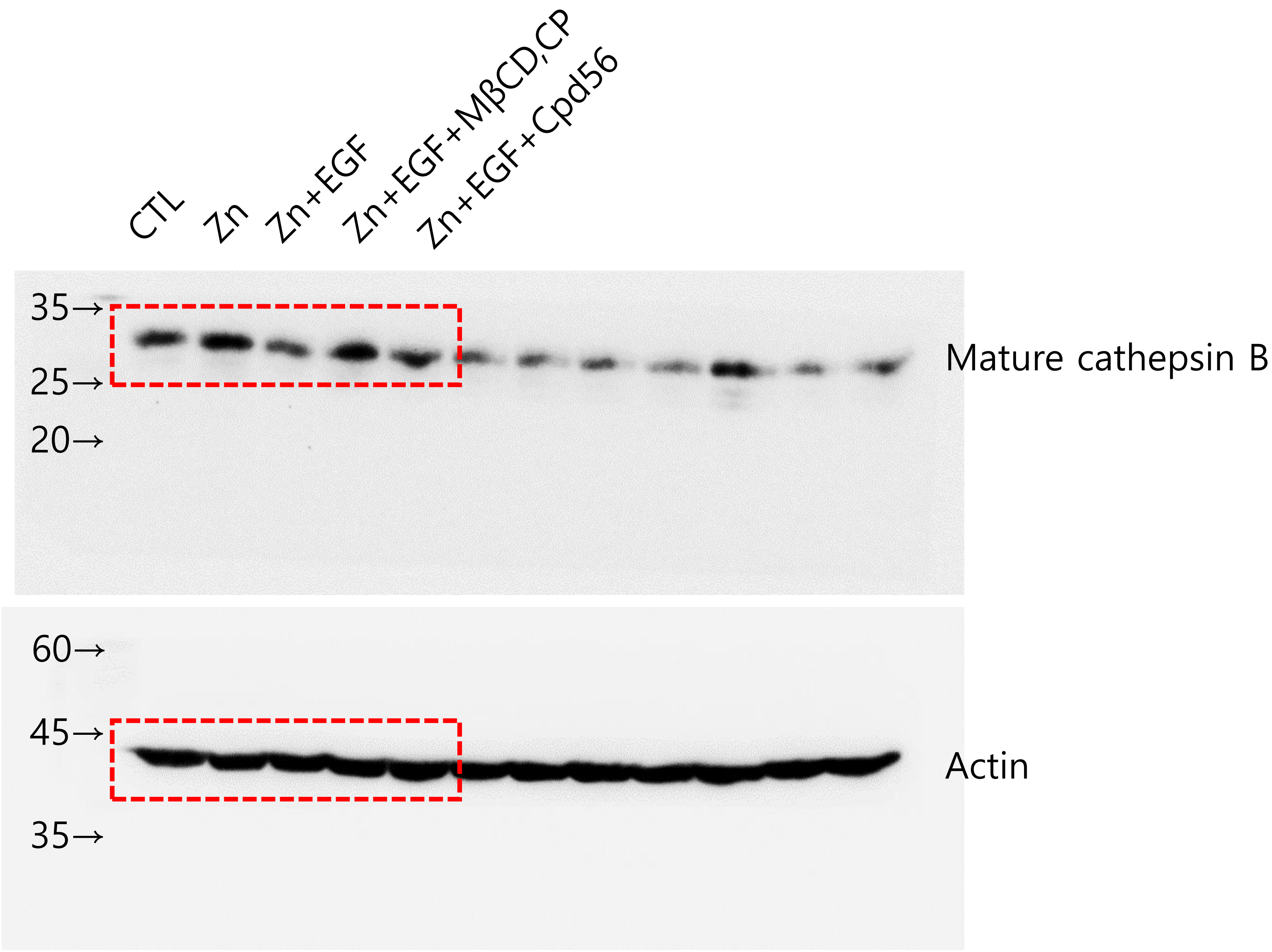

Figure 4D_Mature cathepsin B_MβCD,CP/Cpd56_#3

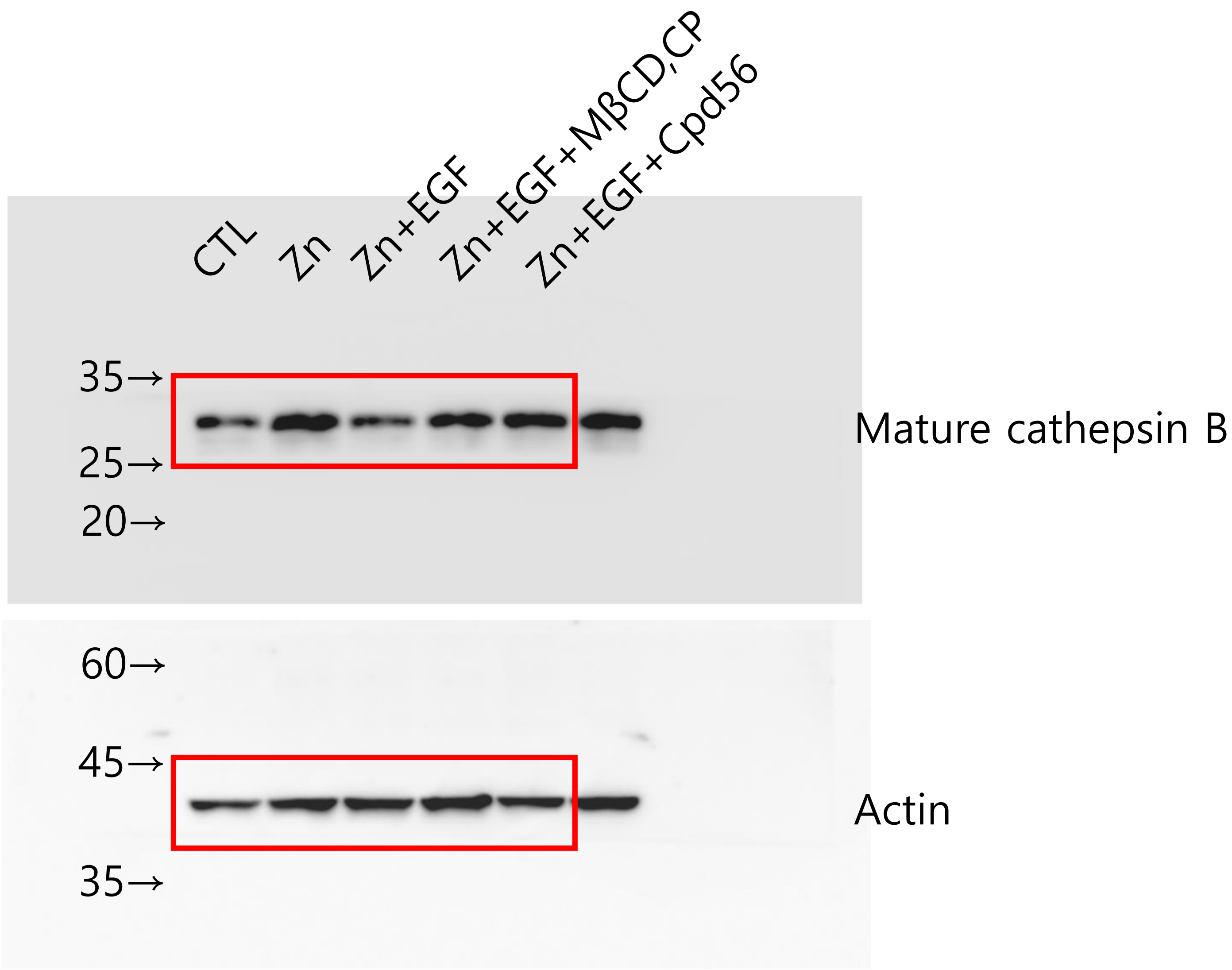

Figure 4D_Mature cathepsin B_MβCD,CP/Cpd56_#4

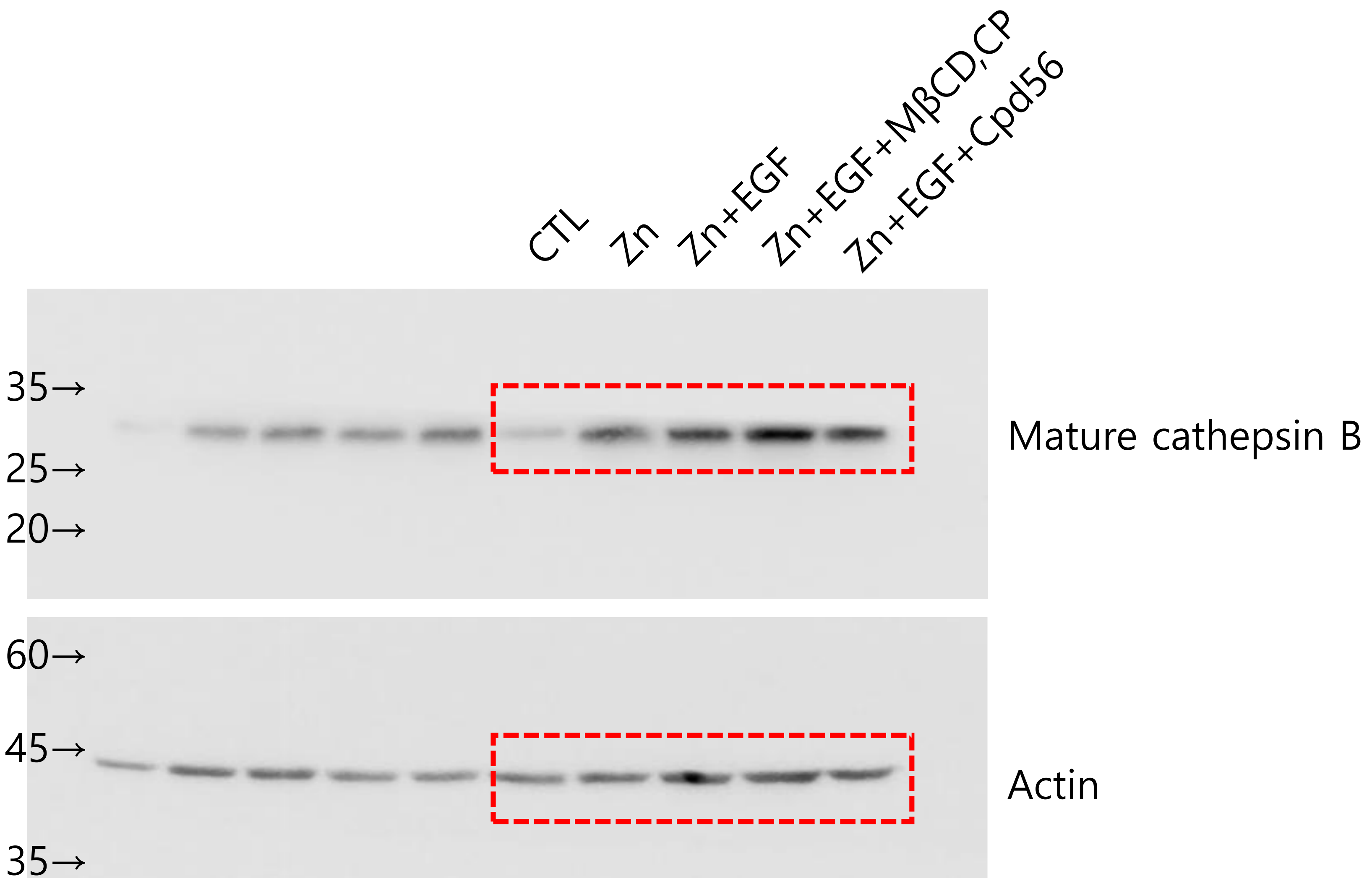

Figure 4D_Mature cathepsin B_MβCD,CP/Cpd56_#5

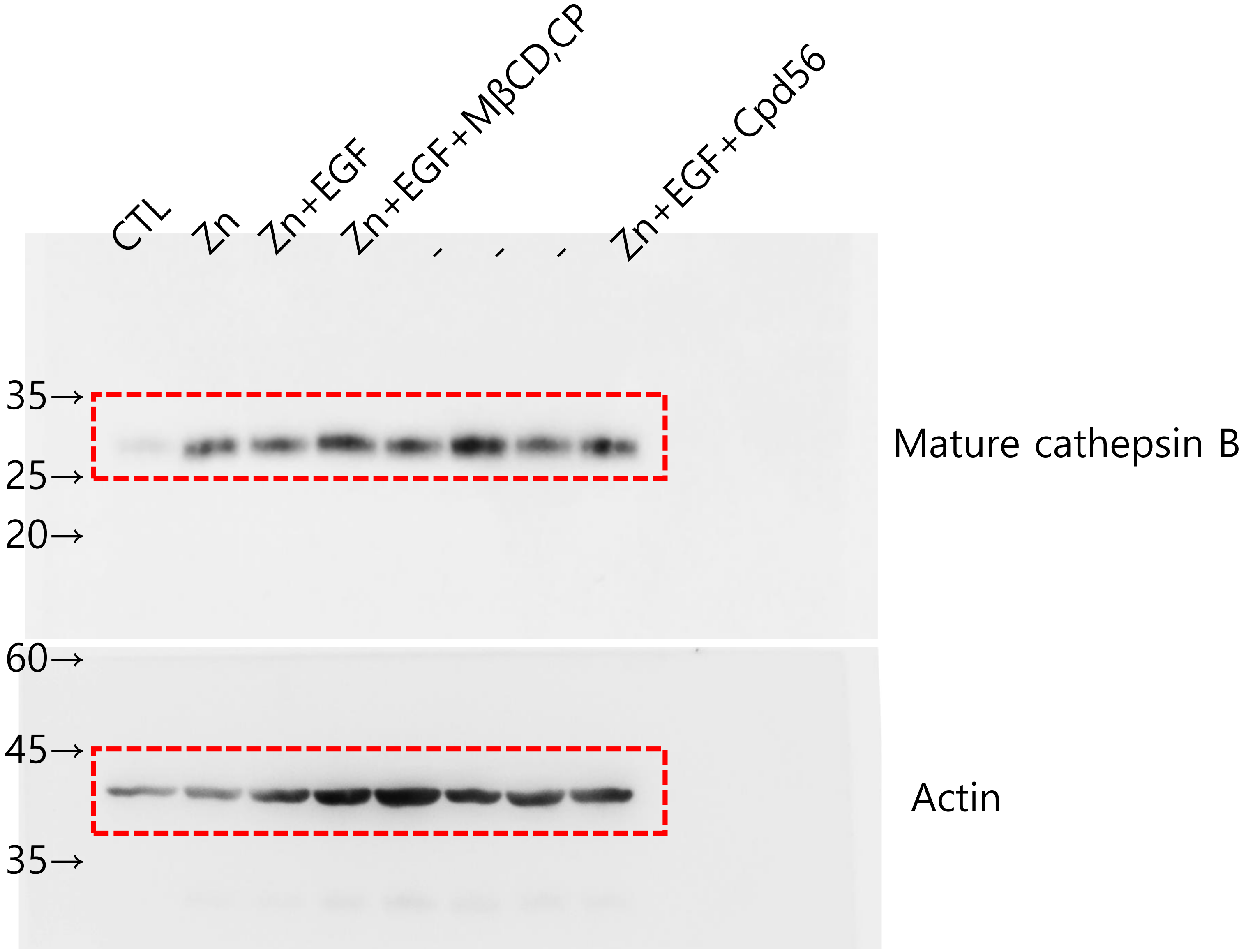

Figure 4D_Mature cathepsin B_MβCD,CP/Cpd56_#6

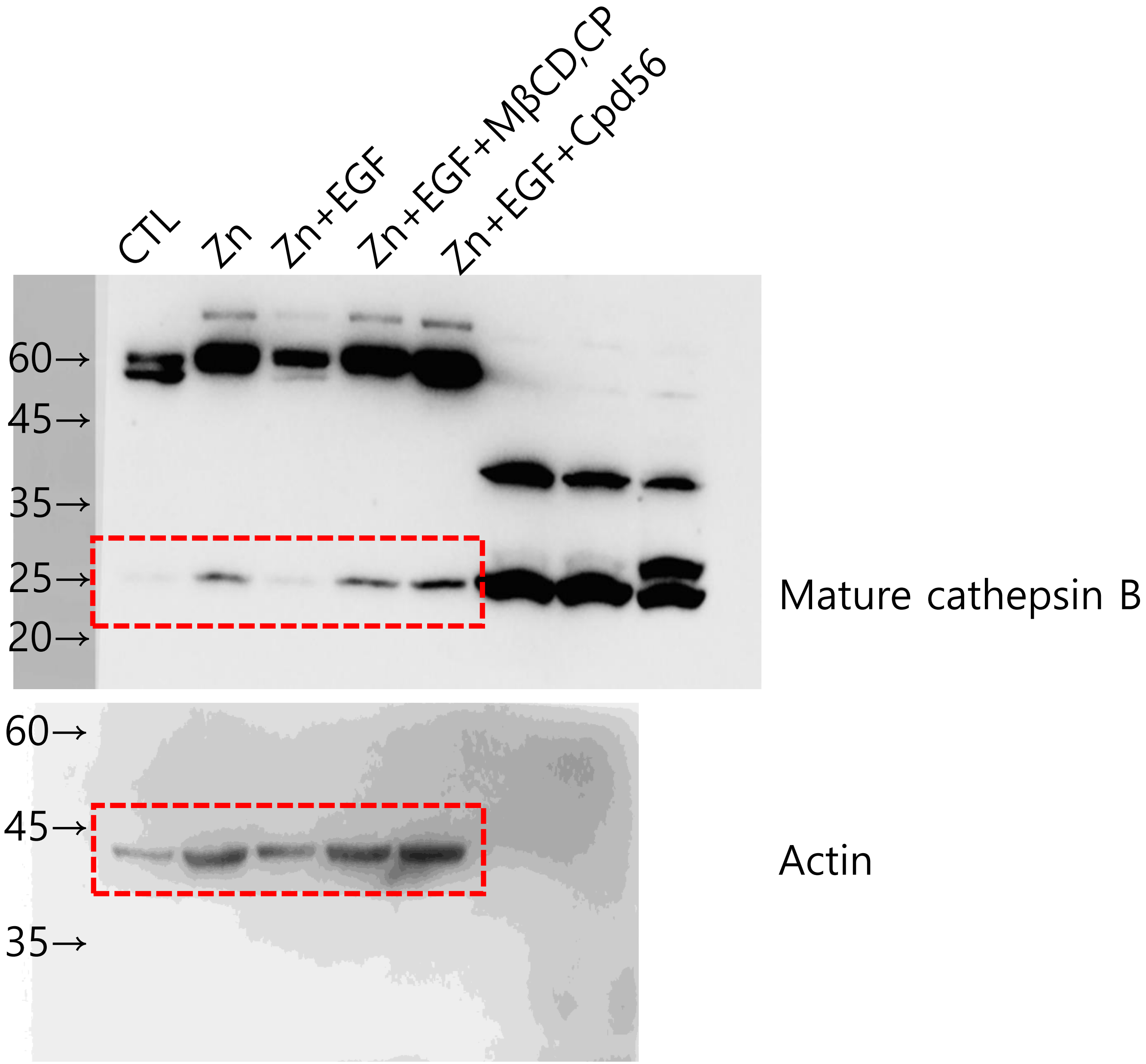

Figure 4D_Mature cathepsin B_Ciliobrevin A__#1

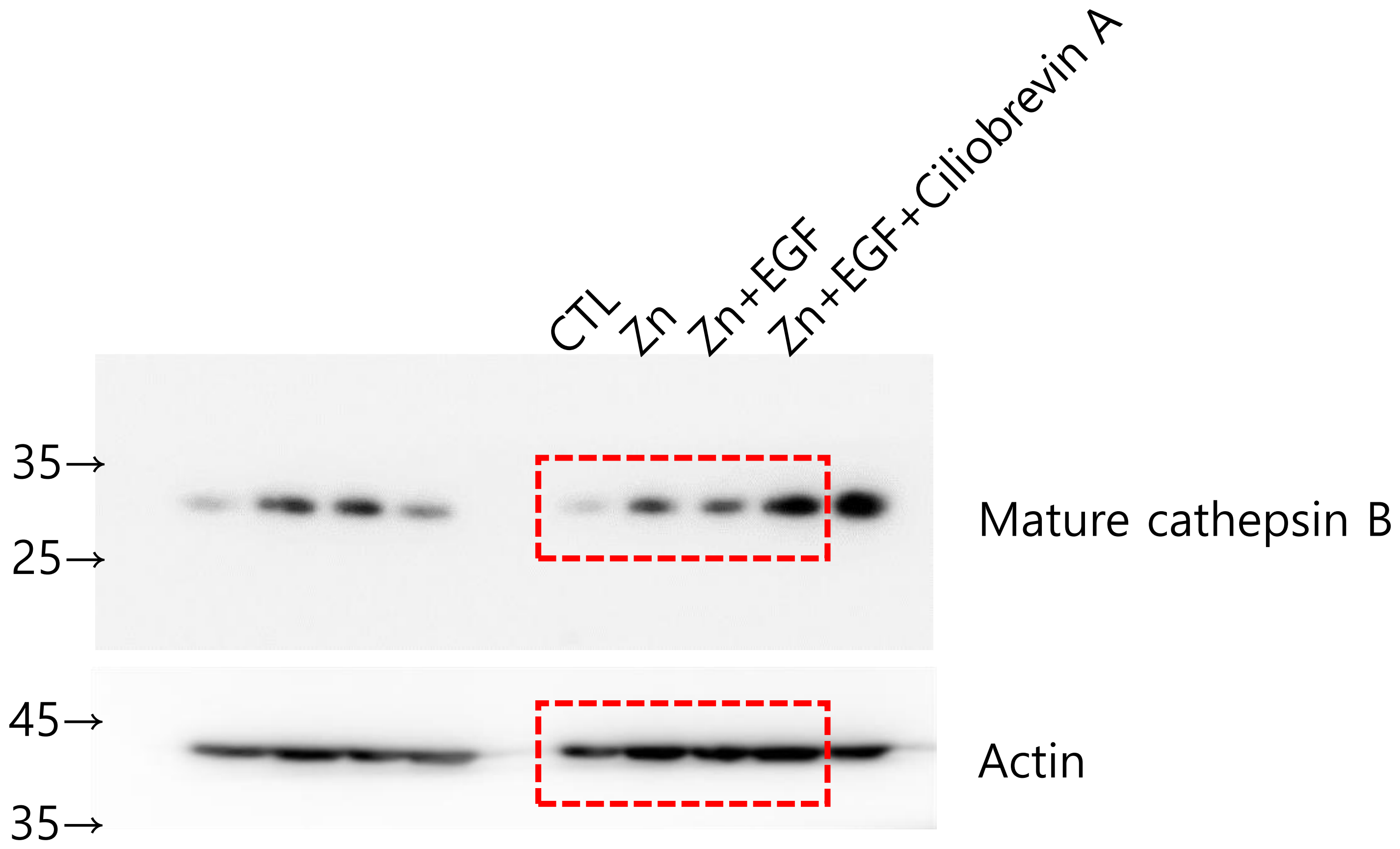

Figure 4D_Mature cathepsin B_Ciliobrevin A__#2

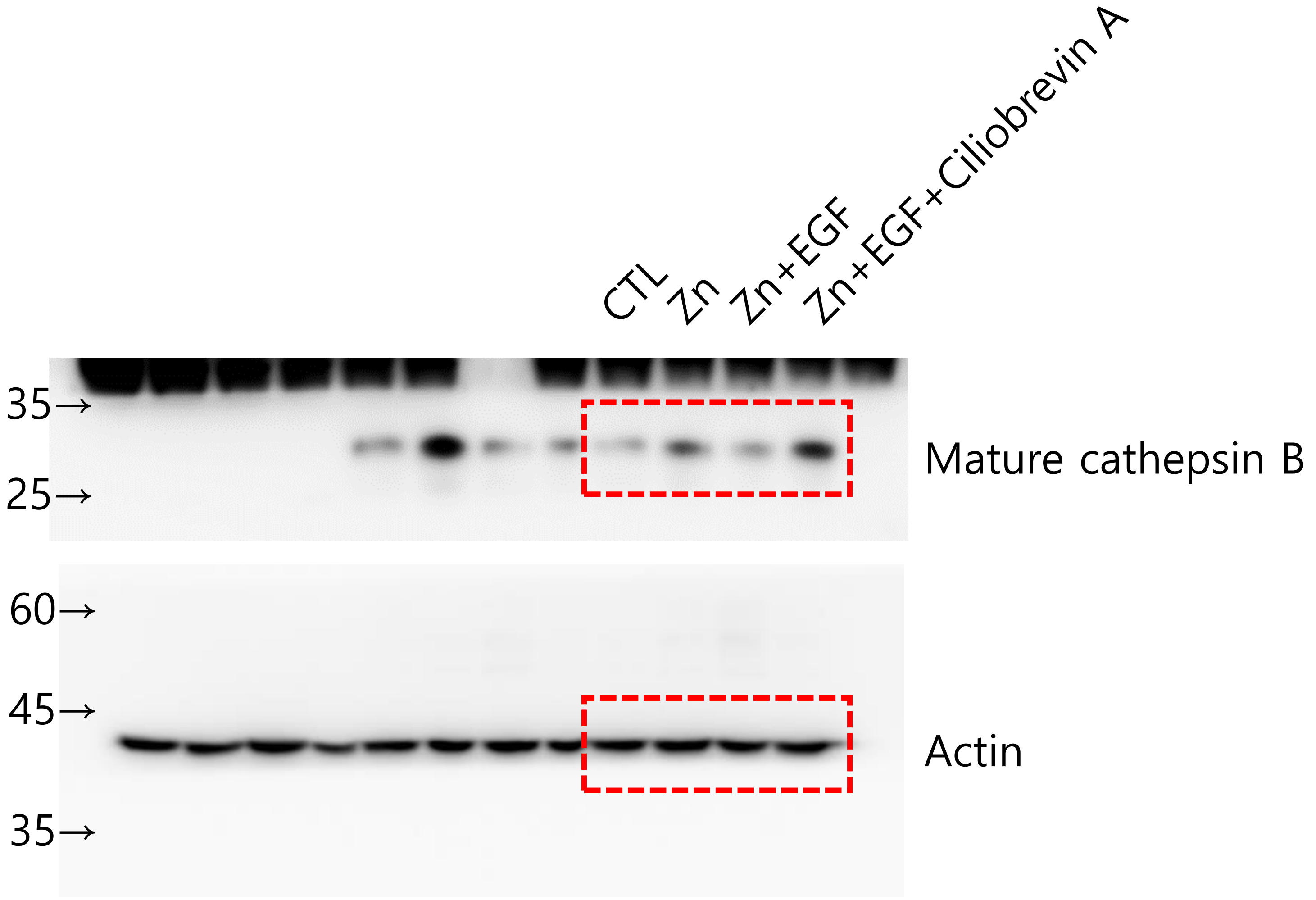

Figure 4D_Mature cathepsin B_Ciliobrevin A__#3

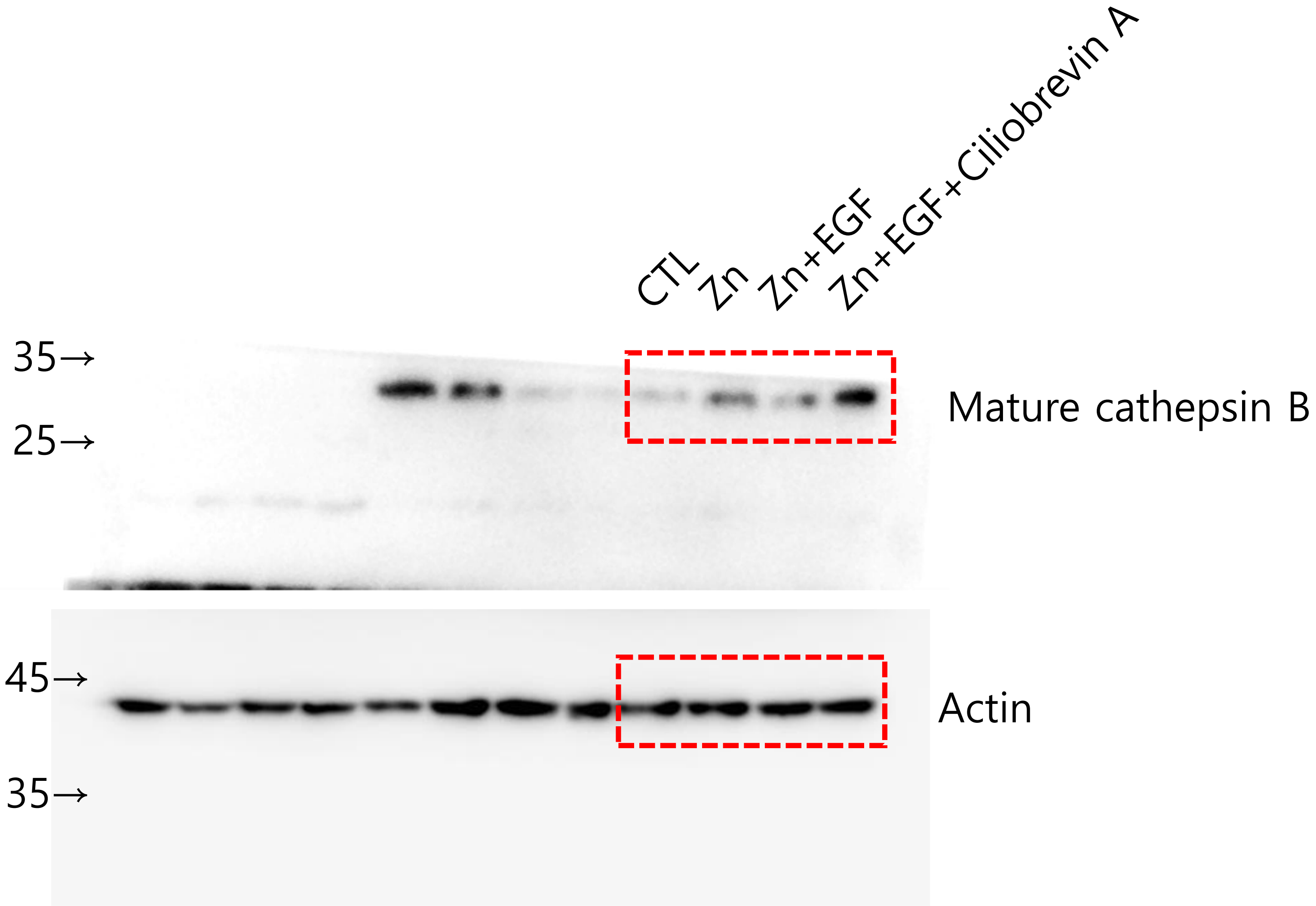

Figure 5E_Mature cathepsin B_#1

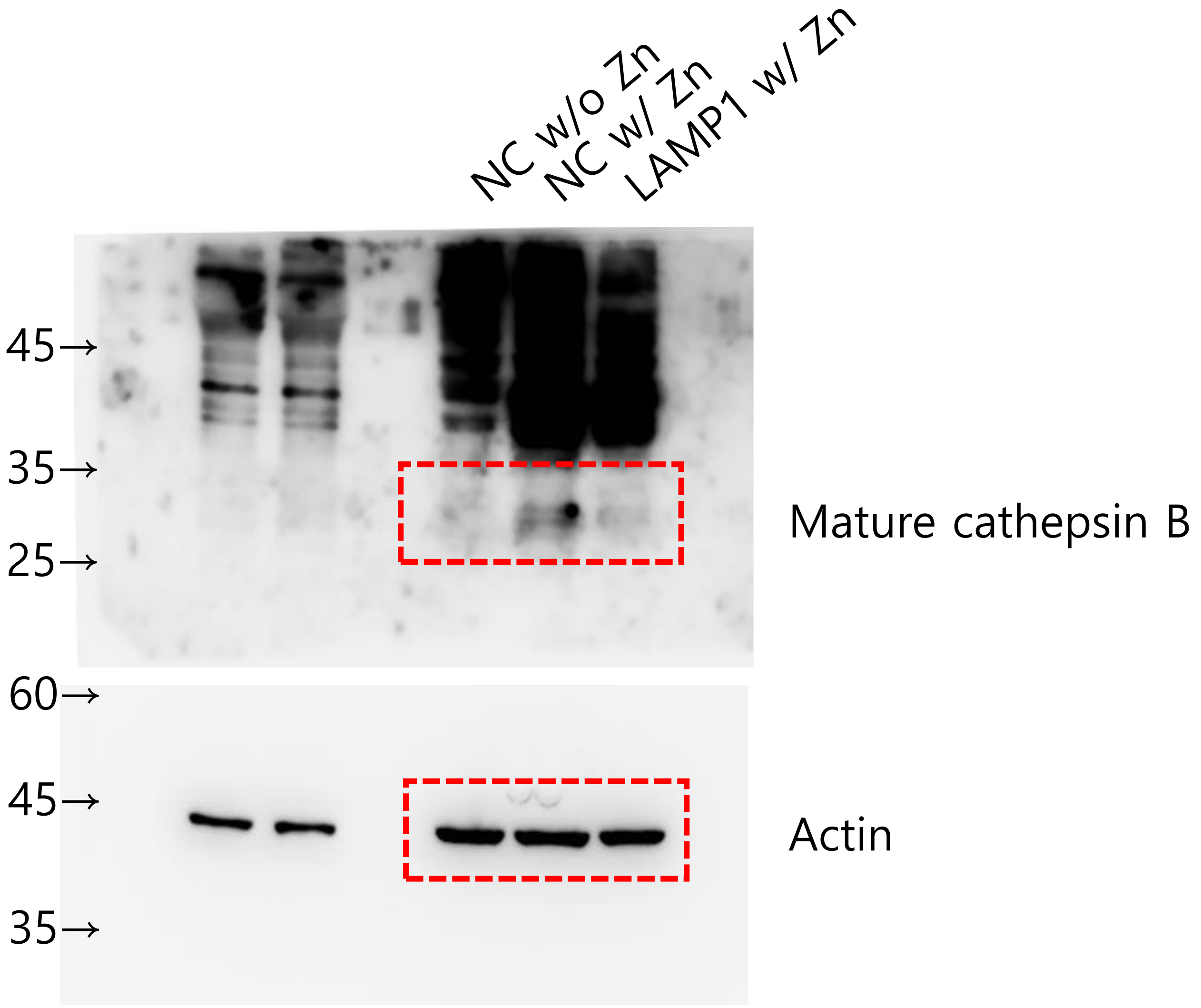

Figure 5E_Mature cathepsin B_#2

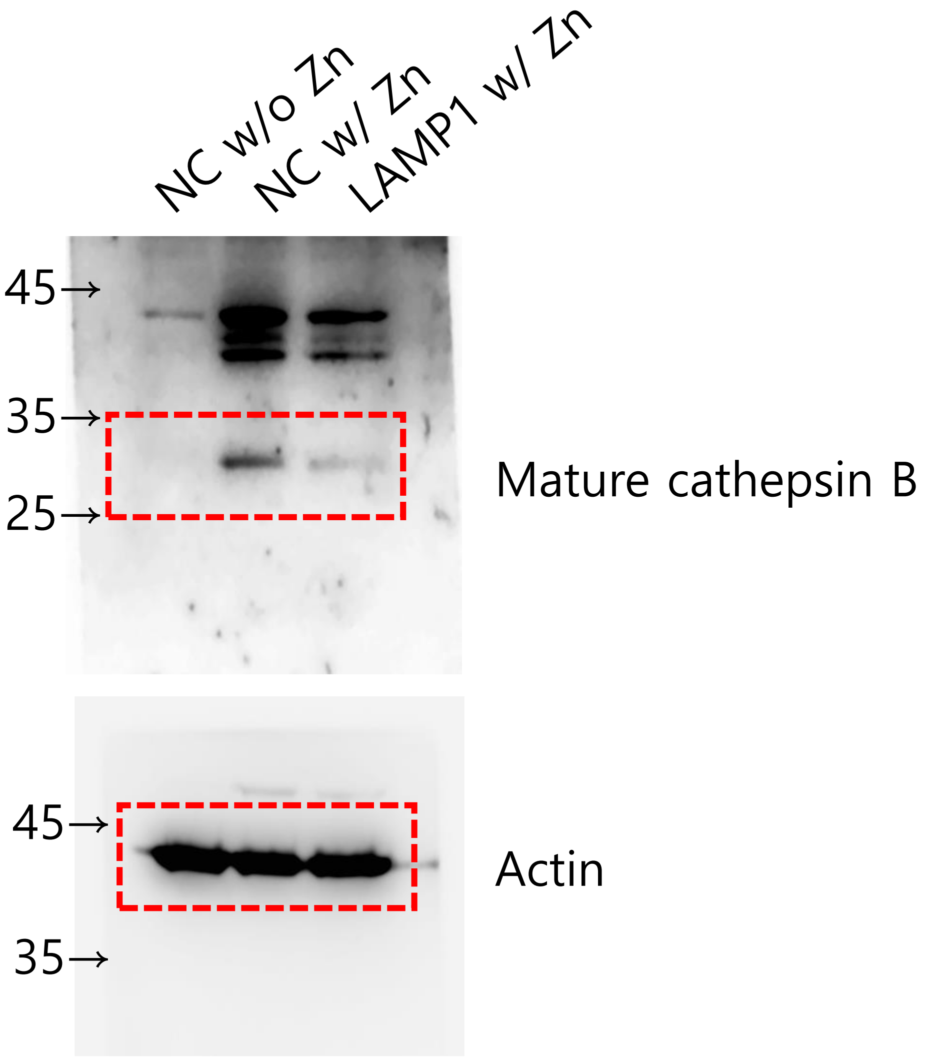

Figure 5E_Mature cathepsin B_#3

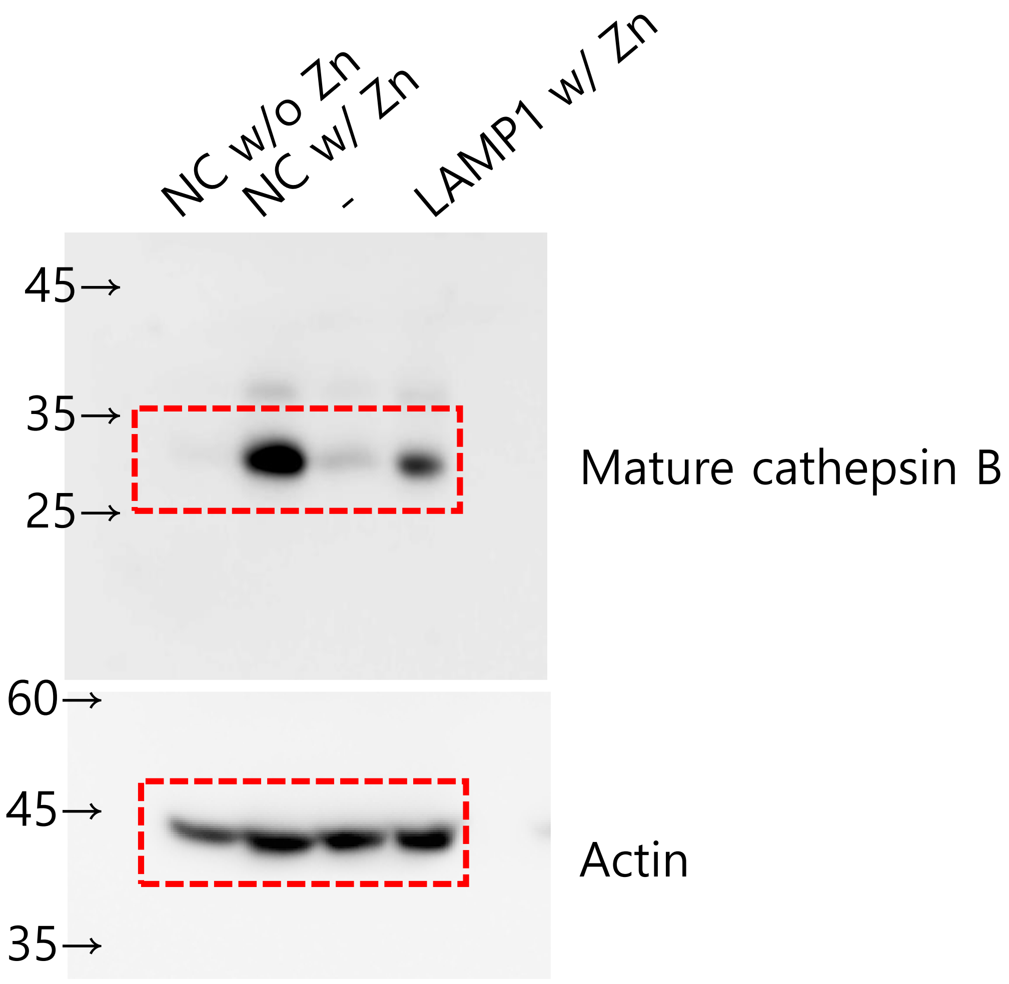

Figure 5E_Mature cathepsin B_#4

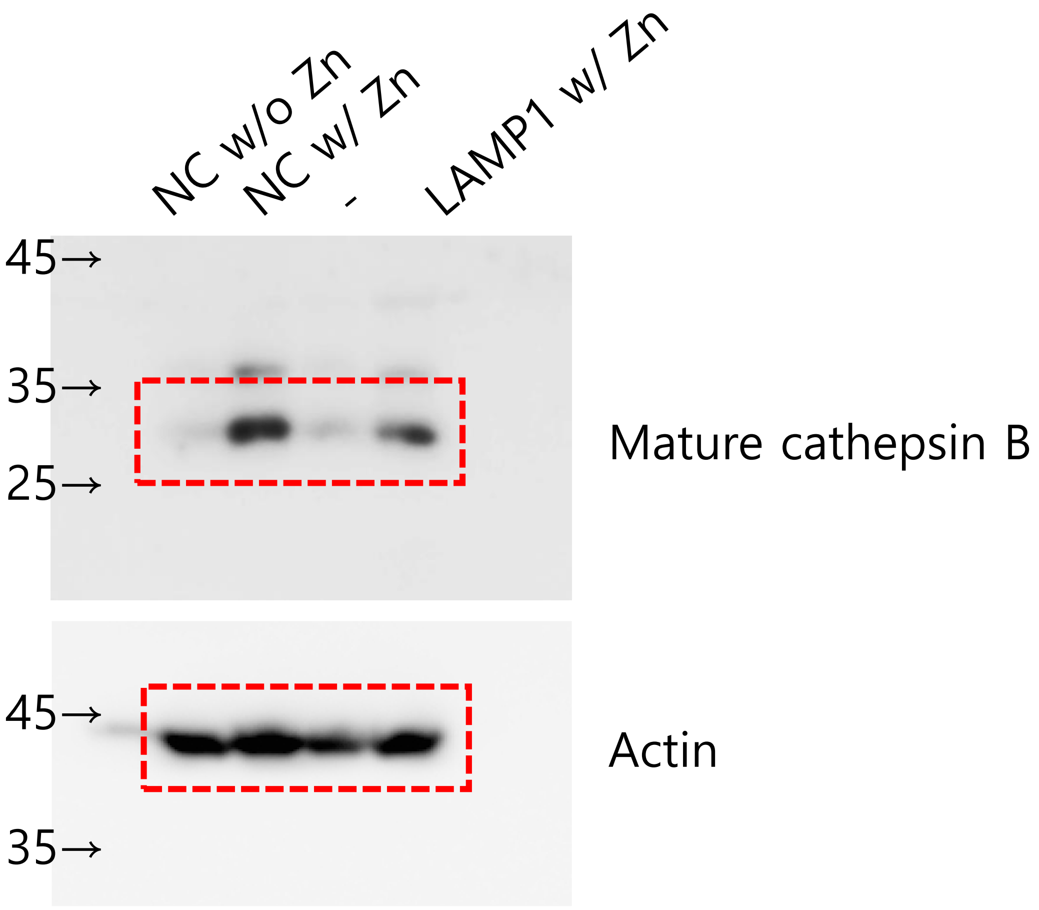

Supplement: Supplementary file 3 — Western Blot source data [file 41419_2024_7192_MOESM3_ESM.pdf]
